# Dual activation of NLRP3 and pyrin inflammasomes mediates host responses to the human fungal pathogen *Coccidioides*

**DOI:** 10.1038/s41467-026-75022-8

**Published:** 2026-07-01

**Authors:** Ka Pui Sharon Yau, Jonathan Rodrigo Erlich, Priscila Rodriguez, Sara McArdle, Cameron J. Nowell, Matthew M. Tate, Janset Onyuru, Ahalya Ratnavel, Aanika Desai, Jane Symington, Guillaume Castillon, Zbigniew Mikulski, Simon Goldstein, Aaron F. Carlin, Joshua Fierer, Theo N. Kirkland, Hal M. Hoffman, Sinem Beyhan, Ben A. Croker

**Affiliations:** 1 Division of Allergy, Immunology, and Rheumatology, Department of Pediatrics, University of California, San Diego, La Jolla, CA USA; 2 Microscopy and Histology Core Facility, La Jolla Institute for Immunology, La Jolla, CA USA; 3 Monash Institute of Pharmaceutical Sciences, Melbourne, VIC Australia; 4 Veterans Affairs San Diego Healthcare System, San Diego, CA USA; 5 Department of Pediatrics, University of California, San Francisco, San Francisco, CA USA; 6 Department of Cellular and Molecular Medicine, University of California, San Diego, La Jolla, CA USA; 7 Division of Infectious Diseases and Global Public Health, Department of Medicine, University of California, San Diego, La Jolla, CA USA; 8 Department of Pathology, University of California, San Diego, La Jolla, CA USA; 9 Rady Children’s Health, San Diego, San Diego, CA USA; 10 Department of Infectious Diseases, J. Craig Venter Institute, La Jolla, CA USA

**Keywords:** Pathogens, Cell death and immune response, Fungal infection, Fungi

## Abstract

*Coccidioides* is a dimorphic fungal pathogen that grows as a mold in soil and produces infectious arthroconidia. Inhaled arthroconidia transition to spherules containing endospores in mammalian hosts. How distinct developmental forms of *Coccidioides* interact with immune cells remains poorly defined. Here, we show that arthroconidia activates TLR2 and induces NLRP3-pyrin inflammasomes in macrophages, while ferroptotic signaling promotes arthroconidia killing. Upon transition to spherules, only ruptured spherules—not intact ones—trigger IL-1β production through NLRP3-pyrin inflammasomes and GSDMD-GSDME pores. A TLR2-NLRP3-pyrin-IL-18 axis in macrophages promotes spherule growth. Endospores activate NLRP3 but not pyrin inflammasomes. Caspase-1-deficient mice show improved disease tolerance to coccidioidomycosis despite equivalent fungal burdens, correlating with enhanced neutrophil extracellular trap formation in lung granulomas. Neutrophils respond to spherules through NLRP3-pyrin inflammasomes, GSDMD-GSDME, and MLKL. Together, these findings reveal that *Coccidioides* morphotypes elicit distinct yet overlapping immune programs, coordinating inflammasome activation and regulated cell death to shape host-pathogen dynamics.

## Abstract (web summary)

*Coccidioides* is a dimorphic fungus, which can transition from arthroconidia to spherules containing endospores in the host. Here the authors characterise the distinct and overlapping innate immune responses to these developmental forms of *Coccidioides*.

## Introduction

C*occidioides* species are soil-dwelling pathogenic fungi that cause coccidioidomycosis, also known as Valley Fever. *Coccidioides* spp. (*C. immitis* and *C. posadasii*) are widely distributed in arid regions of California, Arizona, Texas, Mexico, and parts of Central and South America^1^. Between 1998 and 2023, reported cases of coccidioidomycosis rose from 2271 to over 21,171 cases per year, an increase of more than 900%^2^.

Approximately 60% of human Valley Fever cases are asymptomatic or subclinical. Symptomatic disease most commonly manifests as a self-limited community-acquired pneumonia; however, a subset of patients develops severe or disseminated disease^3^. Extrapulmonary dissemination occurs in fewer than 5% of diagnosed pneumonia cases but carries substantial morbidity, with frequent involvement of the skin, bones, and central nervous system^4–6^. Coccidioidal meningitis is fatal in the absence of lifelong antifungal therapy, underscoring the importance of host immune mechanisms that contain fungal growth and prevent dissemination^7^. Despite effective antifungal drugs, a significant proportion of patients experience persistent or relapsing disease, highlighting gaps in our understanding of protective versus pathological immune responses to *Coccidioides*.

Human genetic and clinical studies point to a central role for innate immune signaling pathways in host resistance to *Coccidioides*. Individuals with mutations affecting C-type lectin receptor signaling, including Dectin-1 gene (*CLEC7A*), *DUOX1*, *DUOXA1*, and *PLCG2*, exhibit increased susceptibility to disseminated coccidioidomycosis, underscoring the importance of Dectin-1 signaling and reactive oxygen species (ROS), such as H_2_O_2_, for host antifungal responses^8^. Individuals with mutations in the interleukin (IL)−12/interferon gamma (IFNγ) pathway and those with low CD4 T cell counts due to human immunodeficiency virus infections are also highly susceptible to disseminated infection, emphasizing the importance of Th1 immunity to mitigate coccidioidomycosis, as is true for many granulomatous infections^9,10^. Together, these observations establish innate immune sensing as a key upstream determinant of disease outcome.

When patients without known immunodeficiencies fail to respond to antifungal therapy, immunomodulators have shown efficacy - most notably recombinant IFN-γ and dupilumab, a neutralizing monoclonal antibody that targets the IL-4 receptor alpha subunit, thereby inhibiting both IL-4 and IL-13 signaling^11^. These successful immunomodulatory therapies underscore the importance of acquiring further knowledge on the immune responses to *Coccidioides*.

*Coccidioides* is a thermally dimorphic fungal pathogen that exists as mold in the environment and transitions into spherules and endospores in mammalian hosts. In the soil, *Coccidioides* form chains of mycelia that segment into individual 3–5 μm spores known as arthroconidia. These spores can be aerosolized during soil-disrupting activities, including farming, construction, and mining. The parasitic life cycle begins with inhalation of arthroconidia. Under the influence of temperature and CO_2,_ arthroconidia undergo a developmental transition to spherules in the host lung that ultimately grow up to 100 μm in diameter, a size that physically limits phagocytosis by macrophages and neutrophils and necessitates alternative immune control strategies^12^. As spherules increase in size, they undergo numerous rounds of internal septation and nuclear division to produce up to 300 endospores per spherule^13,14^. Mature spherules rupture and release endospores that can mature into new spherules, an essential step in the dissemination of *Coccidioides*. Mutants that cannot make endospores are avirulent in mice^13,14^. Thus, *Coccidioides* infection is characterized by dynamic and sequential exposure of the host to fungal morphotypes that differ markedly in size, structure, and cellular accessibility.

Like other fungal pathogens, *Coccidioides* is recognized by host innate immune cells through pattern recognition receptors, including the C-type lectin receptor Dectin-1 (Clec7a) and Toll-like receptor 2 (TLR2)^11^. Signaling through the IL-1 receptor (IL-1R) is also critical for host defense, as it limits fungal dissemination from the lung to the spleen in a mouse model of coccidioidomycosis; however, the cellular origins of IL-1α and IL-1β remain undefined^15^. Consistent with this, mice deficient in caspase recruitment domain-containing protein 9 (CARD9) or myeloid differentiation primary response 88 (MyD88)—key adapter proteins downstream of Dectin-1 and TLR signaling—are more susceptible to subcutaneous *Coccidioides* infection^16^. Furthermore, Dectin-1-deficient (*Clec7a*^*-/-*^) mice exhibit increased fungal burdens in the lungs and spleen, accompanied by marked alterations in bronchoalveolar lavage cytokine profiles, including IL-1β, underscoring the importance of coordinated fungal recognition and inflammatory signaling^17^.

Despite these advances, the cellular mechanisms that link morphotype-specific fungal recognition to inflammasome activation and regulated cell death pathways remain incompletely characterized. Formalin-killed spherules (FKS) induce bone marrow-derived dendritic cells to produce IL-1α, IL-1β, and tumor necrosis factor (TNF) independently of Dectin-1 signaling, whereas Dectin-1 is required for the secretion of IL-6, IL-10, and granulocyte-macrophage colony-stimulating factor (GM-CSF)^17^. Additionally, in peritoneal macrophages stimulated with FKS, both Dectin-1 and MyD88/TIR-domain-containing adapter-inducing interferon-β (TRIF) are required for the production of TNF, IL-6, and macrophage inflammatory protein-2 (MIP-2)^15,17^. Taken together, these studies suggest that Dectin-1 is not solely responsible for the cellular recognition of, and the response to, *Coccidioides*.

Due to its dimorphic lifestyle, *Coccidioides* can interact with host cells in three different forms: arthroconidia, spherules, and endospores. There is a knowledge gap regarding how these different forms of *Coccidioides* interact with host immune cells. In this study, we systematically examined how arthroconidia, spherules, and endospores differentially engage innate immune sensing pathways and downstream effector mechanisms in macrophages and neutrophils. Using genetic and pharmacologic approaches, we dissected the relative contributions of Dectin-1 and TLR2 signaling, as well as inflammasomes, and regulated cell death programs, including pyroptosis, ferroptosis-associated lipid peroxidation, and necroptotic signaling. Our findings reveal a morphology-specific and multifaceted host immune response to *Coccidioides*, characterized by coordinated yet distinct fungal sensing, inflammasome activation, and regulated cell death signaling in macrophages and neutrophils.

## Results

### *Coccidioides* arthroconidia induce macrophage death

**Fig. 1:**
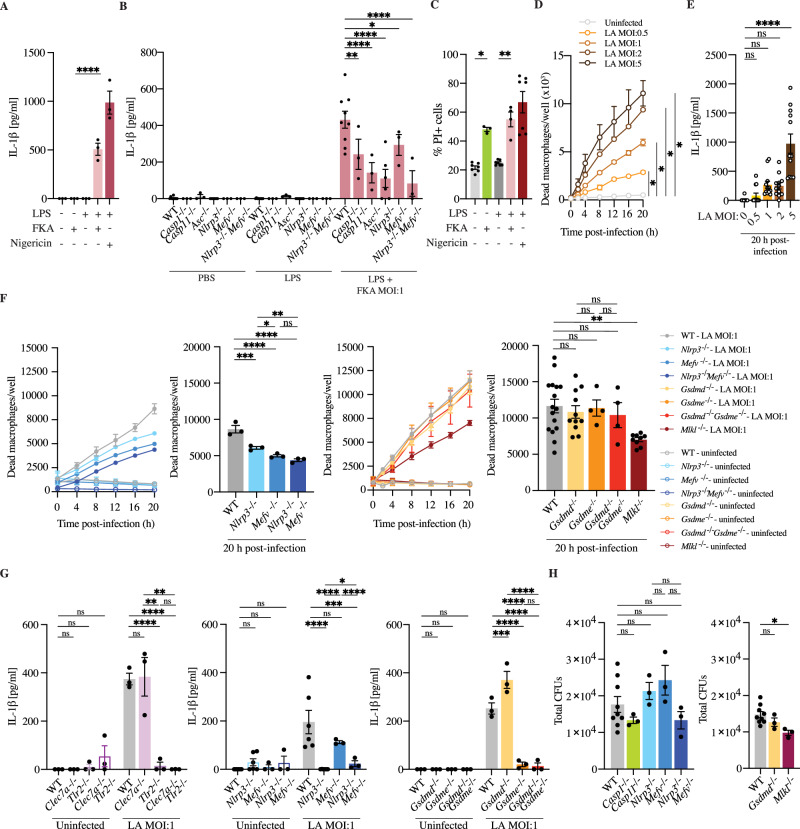
Arthroconidia induce macrophage death. **A** Wild type (WT) (*n* = 3) BMDMs infected with fixed arthroconidia (FKA) at MOI:1, with or without LPS priming. FKA vs LPS + FKA, only significant comparisons shown, one-way ANOVA unpaired. **B** IL-1β release from WT (*n* = 9), *Casp1*^*-/-*^*Casp11*^*-/-*^ (*n* = 3)*, Asc*^*-/-*^ (*n* = 3), *Nlrp3*^*-/-*^ (*n* = 6)*, Mefv*^*-/-*^ (*n* = 3), and *Nlrp3*^*-/-*^*Mefv*^*-/-*^ (*n* = 3) BMDMs infected with FKA at MOI:1, with LPS priming. WT vs knockouts (KOs), single-knockout (SKO) vs double-knockout (DKO) comparisons, only significant comparisons shown, one-way ANOVA unpaired. **C** Cell viability by flow cytometry of WT (*n* = 3–7) BMDMs infected with FKA at MOI:1, with or without LPS priming. LPS vs FKA, only significant comparisons shown, one-way ANOVA unpaired. Gating Strategy is available in Supplementary Fig. 5. Refer to Source data for biological replicates. **D**, **E** WT BMDMs infected with live arthroconidia (LA) at MOI:0.5, 1, 2, and 5. **D** Cell viability by live-cell imaging and automated image analysis (whole well scan) of WT (*n* = 3) BMDMs. Uninfected vs LA, repeated measures (RM) one-way ANOVA unpaired. **E** IL-1β release from WT (*n* = 12) BMDMs 20 h post-infection (hpi). Uninfected vs LA, one-way ANOVA unpaired. **F** Cell viability by live-cell imaging and automated image analysis (whole well scans) of WT (left: *n* = 3, right: *n* = 16)*, Nlrp3*^*-/-*^ (*n* = 3)*, Mefv*^*-/-*^ (*n* = 3)*, Nlrp3*^*-/-*^*Mefv*^*-/-*^ (*n* = 3), *Gsdmd*^*-/-*^ (*n* = 11), *Gsdme*^*-/-*^ (*n* = 4), *Gsdmd*^*-/-*^*Gsdme*^*-/-*^ (*n* = 4), and *Mlkl*^*-/-*^ (*n* = 9) BMDMs infected with LA at MOI:1. WT vs KOs, SKO vs DKO, one-way ANOVA unpaired. **G** IL-1β release from WT (left and right: *n* = 3; middle: *n* = 6)*, Clec7a*^*-/-*^ (*n* = 3)*, Tlr2*^*-/-*^ (*n* = 3)*, Clec7a*^*-/-*^*Tlr2*^*-/-*^ (*n* = 3)*, Nlrp3*^*-/-*^ (*n* = 6)*, Mefv*^*-/-*^ (*n* = 3)*, Nlrp3*^*-/-*^*Mefv*^*-/-*^ (*n* = 3), *Gsdmd*^*-/-*^ (*n* = 3), *Gsdme*^*-/-*^ (*n* = 3), and *Gsdmd*^*-/-*^*Gsdme*^*-/-*^ (*n* = 3) BMDMs infected with LA MOI:1 at 20 hpi. WT vs KOs, two-way ANOVA; SKO vs DKO, one-way ANOVA unpaired. **H** Fungal burden of WT (*n* = 9), *Casp1*^*-/-*^*Casp11*^*-/-*^ (*n* = 3)*, Nlrp3*^*-/-*^ (*n* = 3)*, Mefv*^*-/-*^ (*n* = 3)*, Nlrp3*^*-/-*^*Mefv*^*-/-*^ (*n* = 3), *Gsdmd*^*-/-*^ (*n* = 3), and *Mlkl*^*-/-*^ (*n* = 3) BMDMs infected with LA at MOI:1 20 hpi. WT vs KOs, SKO vs DKO, one-way ANOVA unpaired. Mean ± SEM. Each data point represents a biological replicate. Statistical significance: *P < 0.05, **P < 0.005, ***P < 0.0005, ****P < 0.0001; ns not significant.

To model the initial interaction of the host immune response with *Coccidioides*, we investigated macrophage responses to arthroconidia, specifically IL-1β production and cell death. First, we stimulated murine bone marrow-derived macrophages (BMDMs) with formalin-killed arthroconidia (FKA). FKA failed to induce detectable IL-1β release, suggesting that additional signals are required for IL-1β production (Fig. 1A). Priming BMDMs with lipopolysaccharide (LPS) prior to FKA exposure triggered IL-1β release via the NOD-, LRR-, and pyrin domain-containing protein 3 (NLRP3) inflammasome, but not via the pyrin inflammasome (encoded by *Mefv*) (Fig. 1A, B). Exposure of BMDMs to FKA also resulted in significant macrophage death that occurred independently of LPS priming (Fig. 1C). Live arthroconidia (LA) likewise induced macrophage death and IL-1β release, with the latter more prominent in a dose-dependent manner (Fig. 1D, E).

Insights obtained from research with other fungal pathogens, such as *Candida albicans*, provide a valuable roadmap for understanding how inflammasome pathways might be engaged during *Coccidioides* infection. *C. albicans* activates the NLRP3 inflammasome leading to IL-1β processing and release through plasma membrane pores formed by gasdermin (GSDM)-D^18–21^. Mixed lineage kinase domain-like pseudokinase (MLKL) contributes to IL-1α and IL-1β secretion from myeloid cells in response to *C. albicans* infection by disrupting the plasma membrane through phospholipid binding and oligomerization in the lipid bilayer^22^. *C. albicans*-induced macrophage death involves receptor-interacting protein kinase 3 (RIPK3), MLKL, NLRP3, caspase-1, and the adapter protein ASC^20–23^. These studies suggest that inflammasome activation, gasdermin pore formation, and necroptotic signaling may function in a coordinated manner during antifungal immunity. Indeed, additional priming of BMDMs with Pam_2_CysSerLys_4_ (Pam_2_CSK_4_) prior to FKA challenge was required for IL-1β release, which occurred concomitantly with cleavage of GSDMD, caspase-1, and GSDME (Supplementary Fig. 1). To determine the importance of these pathways to macrophage cell death in the context of *Coccidioides* infections, we used BMDMs deficient in NLRP3, pyrin (*Mefv*), GSDMD, GSDME, or MLKL. Both NLRP3-pyrin inflammasomes and MLKL contributed to macrophage cell death in response to live arthroconidia, whereas loss of GSDMD and GSDME had no detectable impact (Fig. 1F). Thus, arthroconidia-induced macrophage death involves coordinated NLRP3-pyrin inflammasome signaling and MLKL-dependent membrane disruption, but is largely independent of canonical gasdermin-mediated pyroptosis. LA triggered IL-1β release in response to TLR2 sensing but not Dectin-1 (*Clec7a*), and this release was dependent on the NLRP3 inflammasome but not pyrin, as well as on GSDME pore formation (Fig. 1G). Only loss of MLKL reduced arthroconidia viability as measured by colony forming units (CFUs) in LA-infected BMDMs 24 h post-infection, which may be attributable to the increased viability of infected *Mlkl*^*-/-*^ BMDMs (Fig. 1F, H). This finding suggests that MLKL-dependent pathways contribute not only to host cell death but also indirectly to restriction of fungal survival within macrophages. Collectively, these data indicate that arthroconidia engage upstream sensing pathways to drive inflammasome activation, and that cytokine release and cell death can be mechanistically uncoupled during macrophage responses to *Coccidioides*.

### Ferroptotic lipid peroxidation kills arthroconidia without altering macrophage viability

**Fig. 2:**
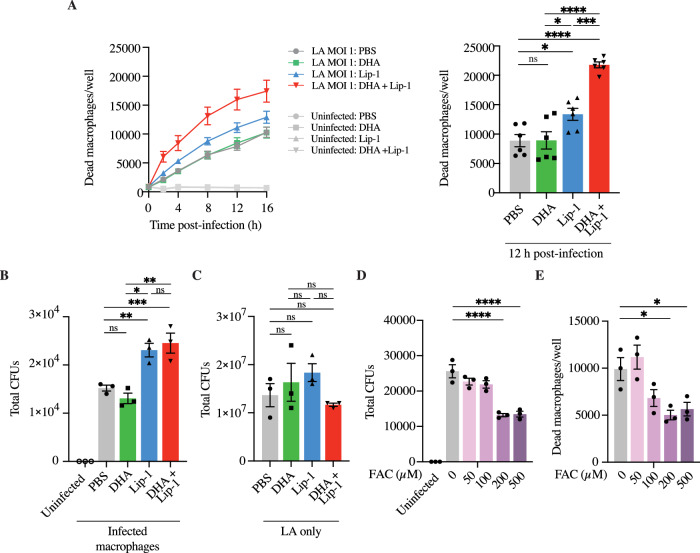
Macrophages activate ferroptotic signaling to counter arthroconidia. **A** Cell viability by live-cell imaging and automated image analysis (whole well scan) of WT (*n* = 13) BMDMs infected with live arthroconidia (LA) at MOI:1, and treated with liproxstatin-1 (Lip-1) and docosahexaenoic acid (DHA). Analysis of WT (*n* = 6) BMDMs at 12 hpi. PBS vs DHA or Lip-1, DHA vs DHA+Lip1, one-way ANOVA unpaired. Viability of **B** LA in WT (*n* = 3) BMDM cultures, and **C** LA (*n* = 3) only on 2X GYE agar plates supplemented with Lip-1 and DHA. PBS vs DHA or Lip-1, DHA vs DHA+Lip1, one-way ANOVA unpaired. **D**, **E** WT (*n* = 3) BMDMs with LA at MOI:1, and treated with ferric citrate (FAC) at 0, 50, 100, 200, and 500 µM. **D** Fungal burden of cells 20 hpi. PBS vs FAC, one-way ANOVA unpaired. **E** Cell viability by live-cell imaging and automated image analysis (whole well scan) of cells 20 hpi. Only significant comparisons shown, PBS vs FAC, one-way ANOVA unpaired. Mean ± SEM. Each data point represents a biological replicate. Statistical significance: *P < 0.05, **P < 0.005, ***P < 0.0005, ****P < 0.0001; ns not significant.

Our findings that LA-infected macrophages died in the absence of GSDMD and GSDME, with only a modest reduction in *Mlkl*^*-/-*^ macrophages, suggested that other regulated cell death pathways beyond pyroptosis and necroptosis might be engaged following infection with *Coccidioides* arthroconidia (Fig. 1). We next considered that ferroptosis, an iron-dependent form of regulated cell death characterized by the accumulation of lipid hydroperoxides and oxidative damage to cellular membranes, is a potential mechanism^24^. To test this, we used liproxstatin-1 (Lip-1), a small-molecule ferroptosis inhibitor. Unexpectedly, Lip-1 did not rescue macrophage death but instead modestly increased cytotoxicity following LA infection (Fig. 2A). Another inhibitor of ferroptotic signaling, ferrostatin-1, also increased macrophage death following LA infection (Supplementary Fig. 2). During ferroptosis, polyunsaturated fatty acids in membranes, including docosahexaenoic acid (DHA), can be targeted by iron-dependent ROS, thereby amplifying cytotoxicity^24^. To examine the roles of DHA and lipid peroxidation in LA-induced macrophage death, we combined Lip-1 and DHA. DHA alone did not significantly affect macrophage death compared to the control; however, combined treatment with DHA and Lip-1 further exacerbated LA-induced macrophage death relative to Lip-1 alone (Fig. 2A). These findings suggest that ferroptotic lipid peroxidation is induced during infection and, rather than driving macrophage demise, may support macrophage viability in the setting of live arthroconidia.

To determine whether ferroptotic signaling influences fungal survival, we quantified fungal burden in LA-infected wild-type (WT) BMDMs treated with Lip-1. After 24 h, infected BMDMs treated with Lip-1 showed increased fungal viability, as measured by CFUs, supporting a role for lipid peroxidation in macrophage-mediated killing of arthroconidia (Fig. 2B). In contrast, direct incubation of arthroconidia with Lip-1 or DHA in the absence of BMDMs did not alter fungal viability (Fig. 2C), indicating that macrophage-dependent lipid peroxidation, rather than direct drug effects on *Coccidioides*, underlies fungal restriction.

Given that iron is required and utilized during ferroptotic signaling, we reasoned that a decline in intracellular iron levels during this process might impair antifungal activity. To promote ferroptotic signaling in BMDMs, we supplemented macrophage cultures with ferric citrate (FAC) resulting in a concentration-dependent reduction in fungal viability and increased macrophage viability compared to controls (Fig. 2D, E). These data suggest that macrophage-mediated killing of arthroconidia is, at least in part, rate-limited by iron-dependent lipid peroxidation and that effective fungal damage correlates with preservation of macrophage viability. Collectively, our data suggest that the independent activation, or coordination, of multiple cell death signaling pathways is important for antifungal ferroptotic lipid peroxidation, IL-1β secretion, and cell death in response to live arthroconidia.

### Fixed spherules trigger dual activation of NLRP3 and pyrin inflammasomes and pyroptosis

**Fig. 3:**
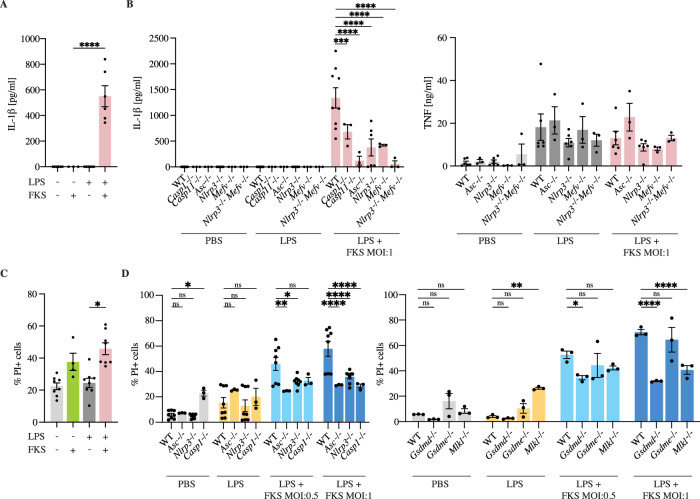
*Coccidioides* spherules induce cytokine release and macrophage death. **A** WT (*n* = 3–6) BMDMs infected with fixed spherules (FKS) at MOI:1, with or without LPS priming. FKS vs LPS + FKS, only significant comparisons shown, two-way ANOVA. Refer to Source data for biological replicates. **B** IL-1β and TNF release from WT (left: *n* = 9, right: *n* = 6), *Casp1*^*-/-*^*Casp11*^*-/-*^ (*n* = 3)*, Asc*^*-/-*^ (*n* = 3), *Nlrp3*^*-/-*^ (*n* = 6)*, Mefv*^*-/-*^ (*n* = 3), and *Nlrp3*^*-/-*^*Mefv*^*-/-*^ (*n* = 3) BMDMs infected with FKS at MOI:1, with LPS priming. WT vs KOs, only significant comparisons shown, two-way ANOVA; SKO vs DKO, only significant comparisons shown, one-way ANOVA unpaired. **C** Cell viability by flow cytometry of WT (*n* = 4–8) BMDMs infected with FKS at MOI:1, with or without LPS priming. LPS vs FKS, one-way ANOVA unpaired. Refer to Source data for biological replicates. **D** Cell viability by flow cytometry of WT (left: *n* = 8, right: *n* = 3), *Asc*^*-/-*^ (*n* = 3), *Nlrp3*^*-/-*^ (*n* = 8), *Casp1*^*-/-*^ (*n* = 3), *Gsdmd*^*-/-*^ (*n* = 3), *Gsdme*^*-/-*^ (*n* = 3), and *Mlkl*^*-/-*^ (*n* = 3) BMDMs infected with FKS at MOI:1, with LPS priming. WT vs KOs, two-way ANOVA. Mean ± SEM. Each data point represents a biological replicate. Statistical significance: *P < 0.05, **P < 0.005, ***P < 0.0005, ****P < 0.0001; ns not significant.

Because arthroconidia induced NLRP3-dependent IL-1β release (Fig. 1B), we next asked whether spherules similarly activate inflammasome pathways in macrophages. WT BMDMs primed with LPS secreted IL-1β following stimulation with formalin-killed spherules (FKS) (Fig. 3A). To determine the role of specific inflammasomes in IL-1β release in response to FKS, we used BMDMs deficient in ASC, NLRP3 or pyrin. Loss of caspase-1/11, ASC, NLRP3, or pyrin each significantly reduced IL-1β production in response to FKS, indicating that spherules activate both NLRP3 and pyrin inflammasomes (Fig. 3B). FKS did not stimulate macrophages to produce more TNF than LPS alone, supporting selective impairment of inflammasome-dependent cytokine release (Fig. 3B). FKS induced BMDM death but only after LPS priming (Fig. 3C). FKS-stimulated macrophage death was reduced in *Asc*^*-/-*^*, Nlrp3*^-/-^, *Casp1*^-/-^, *Gsdmd*^-/-^, and *Mlkl*^-/-^ macrophages (Fig. 3D). Together, these findings indicate that fixed spherules drive dual NLRP3-pyrin inflammasome activation and coordinate ASC-, caspase-1-, GSDMD-, and MLKL-dependent pathways to promote IL-1β secretion and inflammatory cell death.

### Macrophages promote spherule formation in the presence of dead arthroconidia

**Fig. 4:**
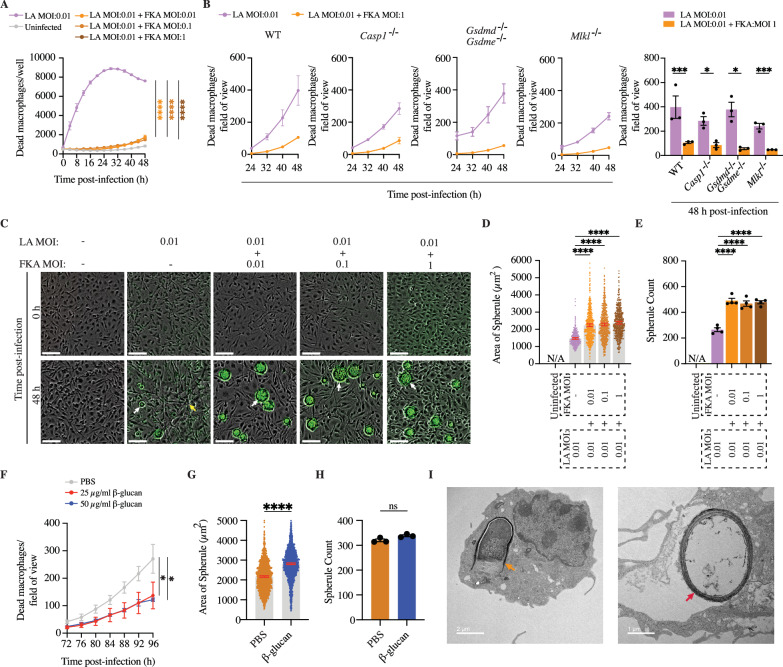
Dead arthroconidia promote spherule formation by live arthroconidia in macrophages. **A** Cell viability by live-cell imaging and automated image analysis (whole well scan) of WT (*n* = 3) BMDMs infected with LA MOI:0.01 and FKA MOI:0.01, 0.1 or 1. LA vs LA + FKA, RM one-way ANOVA unpaired. **B** Cell viability by live-cell imaging and automated image analysis (four fields of view) of WT (*n* = 3), *Casp1*^*-/-*^ (*n* = 3)*, Gsdmd*^*-/-*^*Gsdme*^*-/-*^ (*n* = 3), and *Mlkl*^*-/-*^ (*n* = 3) BMDMs infected with LA MOI:0.01 and FKA MOI:1. Analysis of cells 48 hpi, only significant comparisons shown, LA vs LA + FKA, two-tailed unpaired t-test. **C** WT BMDMs infected with LA MOI:0.01 and FKA MOI:0.01, 0.1, or 1 at 0 and 48 hpi. Representative images from four independent biological replicates are shown. Yellow arrow shows mycelia cluster; white arrows show spherules. Scale bar = 100 µm. **D**, **E** Automated image analysis of WT BMDMs (*n* = 4) in (**C**) by Fungal Finder (FGF) of **D** spherule area and **E** spherule count, mean ± 95% Cl, LA vs LA + FKA, one-way ANOVA unpaired, N/A not applicable. Each data point in (**D**) represents an individual spherule, compiled across independent experiments. **F** Cell viability by live-cell imaging and automated image analysis (four fields of view) of WT (*n* = 3) BMDMs infected with LA MOI:0.01 and FKA MOI:1, and treated with β-glucan at 25, 50 µg/mL. LA vs LA + FKA, RM one-way ANOVA unpaired. **G**, **H** Spherule-infected WT (*n* = 3) BMDMs infected with LA MOI:0.01 and FKA MOI:1, and treated with β-glucan at 25 µg/mL. **G** Spherule area analyzed by FGF, mean with 95% CI, one-way ANOVA unpaired. Data points represent individual spherules. **H** Spherule count analyzed by FGF, one-way ANOVA unpaired. **I** Transmission electron microscopy displaying various stages of the arthroconidia-to-spherule formation within WT BMDMs (LA MOI:0.01 and FKA MOI:0.1) at 48 hpi. Two representative images from sixty fields-of-view are shown. Three independent experiments were performed. Orange arrow shows phagocytosed arthroconidia within macrophages; red arrow shows a developing spherule. Scale bars depicted on image. Mean ± SEM. Each data point represents a biological replicate, unless indicated otherwise. Statistical significance: *P < 0.05, **P < 0.005, ***P < 0.0005, ****P < 0.0001; ns not significant.

Human polymorphonuclear leukocytes and BMDMs are known to support spherule development when infected with arthroconidia^25–27^. We demonstrated that both LA and FKA trigger BMDM death (Fig. 1C, D); however, the fungal recognition and response mechanisms controlling different forms of inflammatory cell death require further examination. To determine the specific effects of LA and FKA fungal recognition, we studied how they affected BMDM cell death signaling. High multiplicity of infection (MOI:1) induced robust early macrophage death and inflammasome activation, whereas low MOI (MOI:0.01) preserved macrophage viability and facilitated spherulation (Figs. 1D and 4). Notably, when WT BMDMs infected with LA were co-exposed to FKA, LA-induced BMDM cell death was attenuated (Fig. 4A). The combination of LA and FKA also reduced the death of *Casp1*^*-/-*^, *Gsdmd*^*-/-*^*Gsdme*^*-/-*^, and *Mlkl*^*-/-*^ BMDMs, with no differences noted between those genotypes, suggesting that FKA-mediated protection from cytotoxicity operates independently of canonical pyroptotic and necroptotic effectors (Fig. 4B). Under low MOI (MOI:0.01), spherules (white arrows) were observed 48 h post-infection in the presence of macrophages, with notable mycelial forms (yellow arrow) (Fig. 4C, Supplementary Movie 1). When LA and FKA were combined at ratios of 1:1, 1:10, and 1:100, live cell imaging showed an increase in the number and size of spherules compared to LA alone, as shown by live-cell imaging (Fig. 4C). To quantify these changes, we developed Fungal Finder (FGF) - a custom-Fiji script that uses an adapted Cellpose model, to identify spherules based on the autofluorescence of their outer walls (Supplementary Fig. 3). FGF analysis confirmed increases in both spherule number and size, even at a 1:1 ratio of LA to FKA (Fig. 4D, E). This approach provides a robust and scalable method to visualize and study the transition of arthroconidia to spherules in vitro.

We hypothesized that FKA addition may provide additional activation signals to macrophages, thereby altering their response to LA and influencing fungal developmental fate. Consistent with this idea, β-glucan supplementation attenuated LA- and FKA-induced macrophage death (Fig. 4F). Consistently, β-glucan treatment promoted spherule development in WT BMDMs without altering the number of spherules (Fig. 4G, H). These data suggest that macrophage activation state influences fungal morphogenesis and that enhanced immune signaling may paradoxically promote spherulation as a potential immune evasion strategy. Electron microscopy images confirmed that BMDMs had phagocytosed arthroconidia (orange arrow), with evidence of transition to spherules (red arrow) occurring by 48 h post-infection (Fig. 4I). Consistent with recent reports demonstrating that phagocytosis facilitates spherule formation in macrophage co-culture systems, our data indicate that macrophage-fungal interactions actively shape *Coccidioides* developmental transitions^25^. These findings open additional avenues for understanding how macrophages and *Coccidioides* interact to influence spherule development.

### Spherule burst triggers IL-1β release

**Fig. 5:**
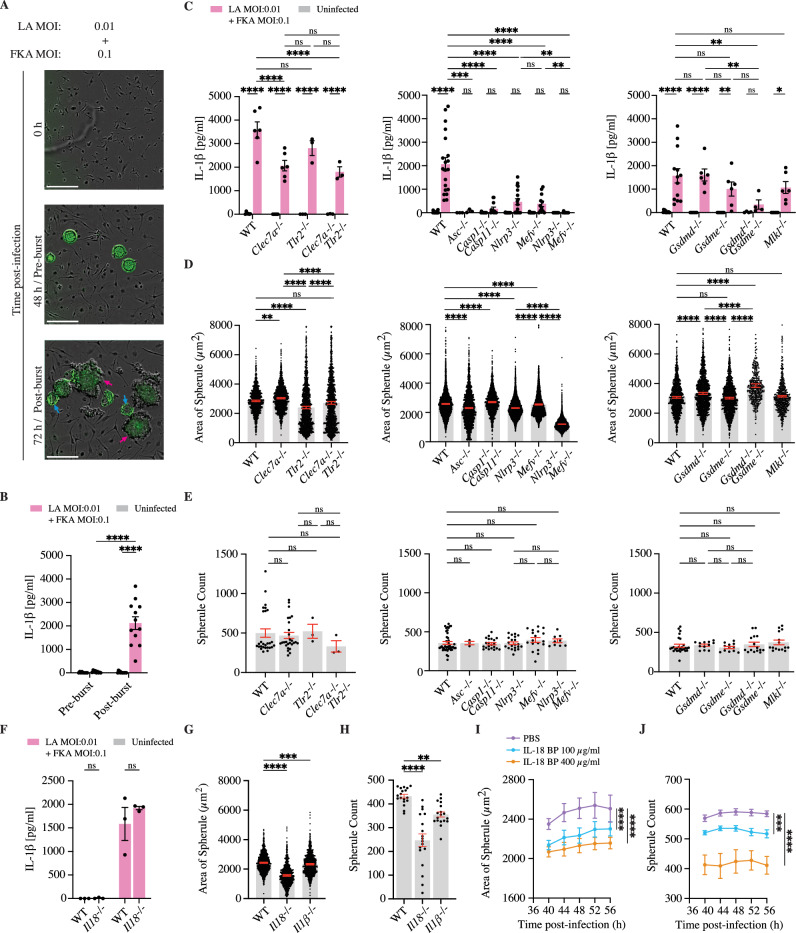
Dual NLRP3/pyrin inflammasomes are activated by rupturing spherules. **A** Representative live-cell images of WT BMDMs infected with LA MOI:0.01 and FKA MOI:0.1 at 0, 48, 72 hpi. Representative images from three independent biological replicates are shown. Pink arrows show endospores released from ruptured spherules; blue arrows show the spherule wall. Scale bar = 200 µm. **B** IL-1β release from spherule-infected WT (*n* = 12) BMDMs, two-tailed unpaired t-test, at “pre-burst” 48 hpi and “post-burst” 72 hpi. **C**–**E** Spherule-infected WT (*n* = 3–21), *Clec7a*^*-/-*^ (*n* = 3–7), *Tlr2*^*-/-*^ (*n* = 3), *Clec7a*^*-/-*^*Tlr2*^*-/-*^ (*n* = 3), *Asc*^*-/-*^ (*n* = 3), *Casp1*^*-/-*^*Casp11*^*-/-*^ (*n* = 4–12), *Nlrp3*^*-/-*^ (*n* = 4–18), *Mefv*^*-/-*^ (*n* = 3–12), *Nlrp3*^*-/-*^*Mefv*^*-/-*^ (*n* = 3–9), *Gsdmd*^*-/-*^ (*n* = 4–7), *Gsdme*^*-/-*^ (*n* = 4–6), *Gsdmd*^*-/-*^*Gsdme*^*-/-*^ (*n* = 3–7), and *Mlkl*^*-/-*^ (*n* = 3–6) BMDMs at post-burst. Refer to Source data for biological replicates. **C** IL-1β release from cells, two-way ANOVA; SKO vs DKO, one-way ANOVA unpaired. **D** Spherule area analyzed by FGF, mean with 95% CI, SKO vs DKO, one-way ANOVA unpaired. Data points represent individual spherules compiled across independent experiments. **E** Spherule count analyzed by FGF (*n* = 3–16), SKO vs DKO, one-way ANOVA unpaired. Data points represent total spherule count per well, compiled across independent experiments. **F** IL-1β release from spherule-infected WT (*n* = 3) and *Il18*^*-/-*^ (*n* = 3) BMDMs at post-burst, two-way ANOVA. **G**, **H** Spherule-infected WT (*n* = 3–6), *Il18*^*-/-*^ (*n* = 3–6), and *Il1b*^*-/-*^ (*n* = 3–6) BMDMs at post-burst, WT vs KOs. Refer to Source data for biological replicates. **G** Spherule area analyzed by FGF, mean with 95% CI, one-way ANOVA unpaired. Data points represent individual spherules, compiled across independent experiments. **H** Spherule count analyzed by FGF, one-way ANOVA unpaired. Data points represent total spherule count per well, compiled across independent experiments. **I**, **J** Time course of spherule-infected WT (*n* = 3) BMDMs supplemented with IL-18 binding protein (IL-18BP) at 100, 400 µg/mL, PBS vs IL-18BP, RM one-way ANOVA unpaired. **I** Spherule area analyzed by FGF, mean with 95% CI. **J** Spherule count analyzed by FGF. Mean ± SEM. Each data point represents a biological replicate, unless indicated otherwise. Statistical significance: *P < 0.05, **P < 0.005, ***P < 0.0005, ****P < 0.0001; ns not significant.

Tracking spherule growth and development with live-cell imaging revealed that spherules consistently rupture at ~72 h post-infection (Fig. 5A). Ruptured spherules were characterized by the release of endospores (pink arrows) and revealed residual fragments of the spherule wall remained (blue arrows) (Fig. 5A, Supplementary Movie 2). Macrophages in “post-burst” cultures at 72 h post-infection produced a robust amount of IL-1β, which was not observed in “pre-burst” cultures at 48 h (Fig. 5B), indicating that spherule rupture, rather than growth alone, is the principal trigger for inflammasome activation. Given the established roles of Dectin-1 and TLR2 in *Coccidioides* sensing^17,28^, we hypothesized that the detection of *Coccidioides* by Dectin-1 and/or TLR2 may alter spherule morphogenesis, so we measured both the cytokine response to spherules and the spherule morphology (Fig. 1G). *Clec7a*^*-/-*^ BMDMs released significantly less IL-1β post-burst, yet still formed larger spherules than WT BMDMs (Fig. 5C, D). In contrast, loss of TLR2 did not significantly alter the amount of IL-1β released under the low-MOI, spherule-inducing conditions; however, the spherules that did form in the presence of *Tlr2*^*-/-*^ BMDMs were smaller and more broadly distributed in size than those grown in the presence of WT or *Clec7a*^-/-^ BMDMs (Fig. 5D). The absence of both Dectin-1 and TLR2 did not further diminish IL-1β, but spherule morphology in *Clec7a*^*-/-*^*Tlr2*^*-/-*^ cultures resembled that observed in *Tlr2*^*-/-*^ BMDMs, highlighting a dominant role for TLR2 signaling in regulating spherule growth dynamics (Fig. 5C, D). Combined deficiency of Dectin-1 and TLR2 did not alter spherule count (Fig. 5E). These data suggest that Dectin-1 signaling is essential for optimal inflammatory cytokine responses to *Coccidioides*, but plays a modest role in restricting spherule growth, whereas TLR2 plays a more prominent role in early responses to arthroconidia and in regulating spherule development (Figs. 1H and 5C–E).

Our earlier results demonstrated that NLRP3 and pyrin inflammasomes are important to detect FKA, LA, and FKS, and for the subsequent release of IL-1β (Figs. 1 and 3). In response to ruptured spherules, IL-1β was reduced in *Nlrp3*^*-/-*^ and *Mefv*^*-/-*^ BMDMs, and abolished in *Asc*^*-/-*^, *Casp1*^*-/-*^*Casp11*^*-/-*^, and *Nlrp3*^*-/-*^*Mefv*^*-/-*^ BMDMs, indicating cooperative activation of both inflammasomes (Fig. 5C). FGF analysis of *Nlrp3*^*-/-*^*Mefv*^*-/-*^ BMDM cultures revealed that spherules were significantly smaller than those in individual *Nlrp3*^*-/-*^ and *Mefv*^*-/-*^ BMDM cultures, suggesting that inflammasome signaling also contributes to optimal spherule maturation (Fig. 5D). These data indicate that both NLRP3 and pyrin inflammasomes are activated by rupturing spherules. Furthermore, individual deficiency of either gasdermin did not alter IL-1β secretion, nor did MLKL deficiency. However, *Gsdmd*^*-/-*^*Gsdme*^*-/-*^ macrophages secreted less IL-1β, supporting a functional redundancy between GSDMD and GSDME downstream of the NLRP3 and pyrin inflammasomes (Fig. 5C). Morphometric analysis of spherule number and size revealed that the combined actions of GSDMD and GSDME restrain spherule size, but not spherule number in BMDM cultures, implicating gasdermin-dependent membrane permeabilization in regulating fungal growth dynamics (Fig. 5D, E).

These findings prompted us to examine the roles of IL-1β and IL-18 in spherule growth. We used BMDMs deficient in IL-1β or IL-18, both of which are processed by caspase-1 via the NLRP3 inflammasome and released through GSDMD pores^29^. Loss of IL-18 did not affect IL-1β release from BMDMs in response to rupturing spherules, but restricted spherule growth and number (Fig. 5F–H). IL-1β deficiency also reduced spherule growth and number, though to a lesser degree than IL-18 deficiency (Fig. 5F–H). The role of IL-18 in spherule growth was further assessed by supplementing BMDM cultures with recombinant human IL-18 binding protein (IL-18BP), which can also neutralize mouse IL-18 to prevent receptor engagement^30^. IL-18BP treatment reduced spherule size and number in a dose-dependent manner, indicating that IL-18 autocrine and/or paracrine signaling is important for spherule maturation (Fig. 5I, J).

### Endospores contribute to IL-1β release

**Fig. 6:**
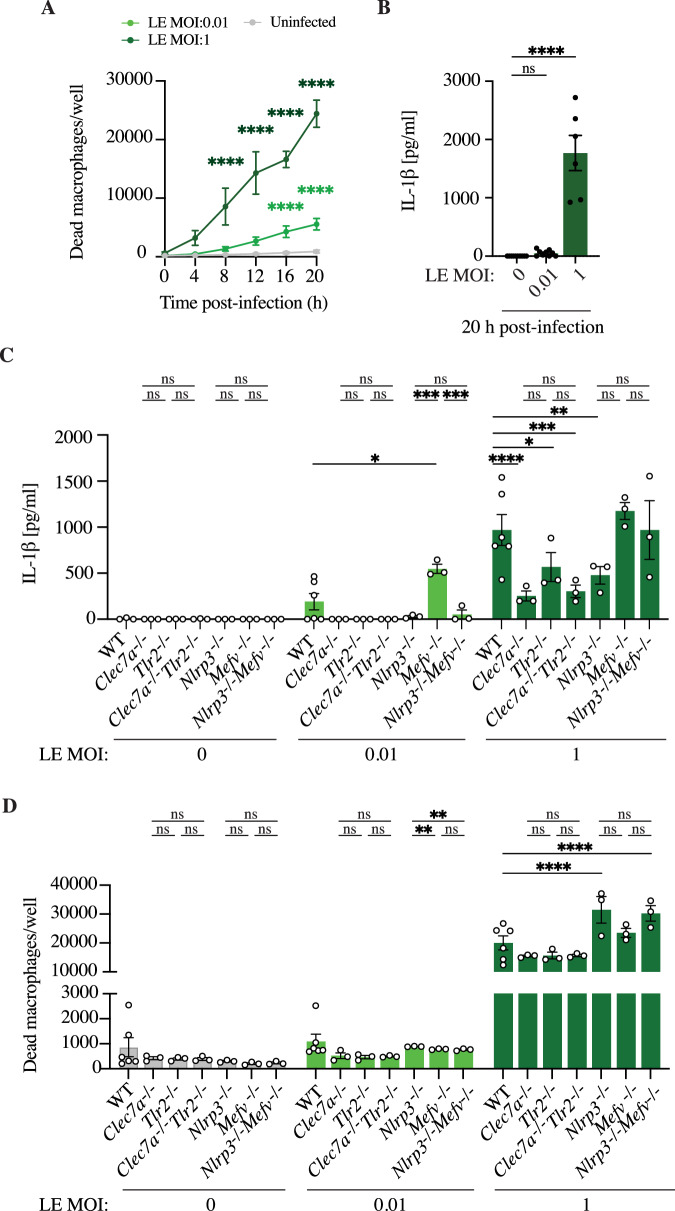
Endospores contribute to IL-1β release from macrophages when spherules rupture. **A**, **B** WT (*n* = 6–9) BMDMs infected with live endospores (LE) at MOI:0.01 and 1. Refer to Source data for biological replicates. **A** Cell viability by live-cell imaging and automated image analysis (whole well scan) of cells. Uninfected vs infected, only significant comparisons shown, two-way ANOVA. **B** IL-1β release from cells at 24 hpi, uninfected vs infected, one-way ANOVA unpaired. **C** IL-1β release from LE-infected WT (*n* = 3–6), *Clec7a*^*-/-*^ (*n* = 3), *Tlr2*^*-/-*^ (*n* = 3), *Clec7a*^*-/-*^*Tlr2*^*-/-*^ (*n* = 3), *Nlrp3*^*-/-*^ (*n* = 3), *Mefv*^*-/-*^ (*n* = 3), and *Nlrp3*^*-/-*^*Mefv*^*-/-*^ (*n* = 3) BMDMs at MOI:0.01 and 1 at 20 hpi. *n* = 3–6, WT vs KOs, SKO vs DKO, all comparisons shown, one-way ANOVA unpaired. Refer to Source data for biological replicates. **D** Cell viability by live-cell imaging and automated image analysis (whole well scan) of WT (*n* = 6), *Clec7a*^*-/-*^ (*n* = 3), *Tlr2*^*-/-*^ (*n* = 3), *Clec7a*^*-/-*^*Tlr2*^*-/-*^ (*n* = 3), *Nlrp3*^*-/-*^ (*n* = 3), *Mefv*^*-/-*^ (*n* = 3), and *Nlrp3*^*-/-*^*Mefv*^*-/-*^ (*n* = 3) BMDMs at MOI:0.01 and 1 at 20 hpi. WT vs KOs, SKO vs DKO, all comparisons shown, one-way ANOVA unpaired. Mean ± SEM. Each data point represents a biological replicate. Statistical significance: *P < 0.05, **P < 0.005, ***P < 0.0005, ****P < 0.0001; ns not significant.

Live-cell imaging of rupturing spherules demonstrated the release of endospores in the presence of macrophages (Fig. 5A, Supplementary Movie 3). We therefore hypothesized that endospores contribute to IL-1β release in post-burst cultures. To test this, endospores were isolated from post-burst BMDM cultures. Infection of naïve BMDMs with the endospore filtrate resulted in rapid spherule formation within 24 h, confirming successful recovery of live endospores (LE) (Supplementary Movie 3). Notably, spherule growth occurred only when LE were cultured in the presence of BMDM (Supplementary Movie 3). In contrast, when cultured alone in macrophage growth medium, LE formed hyphae, highlighting the importance of LE-BMDM interaction for the parasitic life cycle (Supplementary Movie 3). Infection of macrophages with LE at MOI:1 triggered substantial BMDM cell death 8 h post-infection, whereas a lower MOI:0.01 resulted in less cell death (Fig. 6A). Consistent with this dose-dependent effect, IL-1β release was only observed at MOI:1 24 h post-infection, with negligible IL-1β release at MOI:0.01 (Fig. 6B). Dectin-1 deficiency reduced IL-1β secretion, but neither Dectin-1 nor TLR2 significantly influenced BMDM death (Fig. 6C, D). Like the response to ruptured spherules, LE-induced IL-1β release was partially dependent on NLRP3 but not pyrin, suggesting selective inflammasome engagement by endospores (Fig. 6C, D). While post-burst cultures contain LE, they also contain remnants of spherule debris that may serve to activate BMDMs, thereby enhancing the response to LE and the release of IL-1β. Thus, while these data support a direct role for endospores in inflammasome activation, synergistic sensing of co-released spherule components may amplify the inflammasome response in post-burst settings.

### Activation of caspase-1/11 exacerbates coccidioidomycosis in mice

**Fig. 7:**
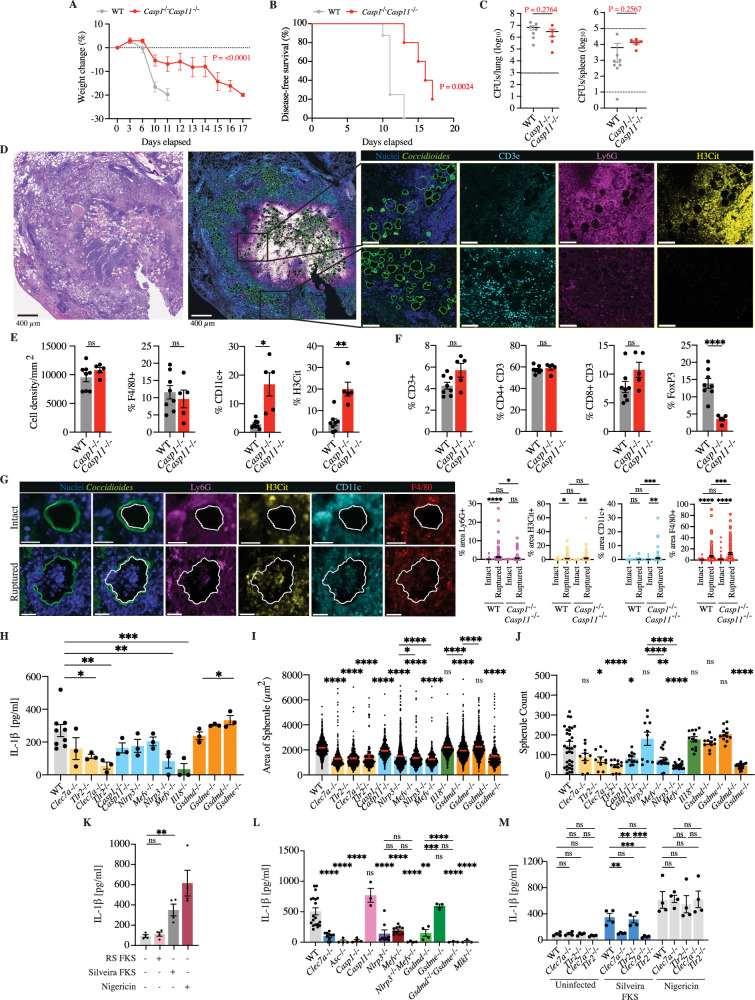
Activation of Caspase-1/11 contributes to lethal coccidioidomycosis. **A**–**G** WT (*n* = 8) and *Casp1*^*-/-*^*Casp11*^*-/-*^ (*n* = 5) mice after intranasal infection with 10^2^ LA. *P* values as shown. **A** Percentage weight change (RM two-way ANOVA) and **B** disease-free survival (log-rank Mantel-Cox). **C** Lung and spleen burden (CFUs) at ethical endpoint (≥20% weight loss). Dotted line indicates the limit of detection. Mixed-effects two-way ANOVA. **D** Immunohistological staining of infected lung samples with Hoechst or marker-specific ArgoFluor-conjugated antibodies. Scale bar = 200 µm, unless indicated otherwise. **E**, **F** Quantification of neutrophil function, resident and infiltrating macrophages, and T cell responses within a lung granuloma. Two-tailed unpaired t-test. **G** Immunohistological staining of immune cells within intact or ruptured spherules. Solid white lines indicate the spherule wall boundary. Scale bar = 20 µm. Marker signal area within the spherule wall relative to total spherule area. *n* (number of spherules annotated) = 61–171, one-way ANOVA unpaired. **H**–**J** Spherule-infected WT (*n* = 9-12), *Clec7a*^*-/-*^ (*n* = 3), *Tlr2*^*-/-*^ (*n* = 3), *Clec7a*^*-/-*^*Tlr2*^*-/-*^ (*n* = 3), *Casp1*^*-/-*^*Casp11*^*-/-*^ (*n* = 3)*, Nlrp3*^*-/-*^ (*n* = 3), *Mefv*^*-/-*^ (*n* = 3), *Nlrp3*^*-/-*^*Mefv*^*-/-*^ (*n* = 3), *Il18*^*-/-*^ (*n* = 3), *Gsdmd*^*-/-*^ (*n* = 3), *Gsdme*^*-/-*^ (*n* = 3), and *Gsdmd*^*-/-*^*Gsdme*^*-/-*^ (*n* = 3) alveolar macrophages (AVMs) at pre-burst, SKO vs DKO, one-way ANOVA unpaired. Refer to Source data for biological replicates. **H** IL-1β release from AVMs. **I** Spherule area analyzed by FGF, mean with 95% CI. Data points represent individual spherules, compiled across independent experiments. **J** Spherule count analyzed by FGF. Data points represent total spherule count per well, compiled across independent experiments. **K** IL-1β release from *C. immitis* RS and *C. posadasii* Silveira FKS-infected WT (*n* = 4) bone marrow neutrophils (BMNs) at MOI:0.2 at 2 hpi, with IFN-γ + LPS priming. Uninfected vs FKS, one-way ANOVA unpaired. **L** IL-1β release from *C. posadasii* Silveira FKS-infected WT (*n* = 19), *Clec7a*^*-/-*^ (*n* = 10), *Asc*^*-/-*^ (*n* = 4), *Casp1*^*-/-*^ (*n* = 4), *Casp11*^*-/-*^ (*n* = 3), *Nlrp3*^*-/-*^ (*n* = 8), *Mefv*^*-/-*^ (*n* = 10)*, Nlrp3*^*-/-*^*Mefv*^*-/-*^ (*n* = 4), *Gsdmd*^*-/-*^ (*n* = 4), *Gsdme*^*-/-*^ (*n* = 3), *Gsdmd*^*-/-*^*Gsdme*^*-/-*^ (*n* = 3), and *Mlkl*^*-/-*^ (*n* = 3) BMNs at MOI:0 or 0.2 at 2 hpi, with IFN-γ + Pam_2_CysSerLys_4_ priming. WT vs KOs, SKO vs DKO, all comparisons shown, one-way ANOVA unpaired. **M** IL-1β release from *C. posadasii* Silveira FKS-infected WT (*n* = 4), *Clec7a*^*-/-*^ (*n* = 4), *Tlr2*^*-/-*^ (*n* = 4), and *Clec7a*^*-/-*^*Tlr2*^*-/-*^ (*n* = 4) BMNs at MOI:0.2 at 2 hpi with IFN-γ + LPS priming. WT vs KOs, two-way ANOVA; SKO vs DKO, all comparisons shown, one-way ANOVA unpaired. Mean ± SEM. Each data point represents a biological replicate, unless indicated otherwise. Statistical significance: *P < 0.05, **P < 0.005, ***P < 0.0005, ****P < 0.0001; ns not significant.

Our findings on *Coccidioides*-macrophage interactions suggest that *Coccidioides* infection triggers overlapping but mechanistically separable inflammatory responses in the host. To further investigate the role of inflammasome-related pathways in disease progression and fungal burden, we monitored weight loss, fungal burden, and survival in WT and *Casp1*^*-/-*^*Casp11*^*-/-*^ mice infected with 10^2^ CFUs of LA. WT mice had the shortest survival, and all exceeded 20% weight loss by day 11 post-infection (Fig. 7A, B). In contrast, *Casp1*^*-/-*^*Casp11*^*-/-*^ mice significantly improved survival and delayed weight loss (Fig. 7A, B). Fungal burden in the lungs and spleen did not differ between WT and *Casp1*^*-/-*^*Casp11*^*-/-*^ mice, indicating that caspase-1/11-deficiency improves disease tolerance rather than enhancing fungal clearance (Fig. 7C). These findings suggest that inflammasome activation contributes to immunopathology during coccidioidomycosis.

Host resistance to *Coccidioides* involves both innate and adaptive immune cells particularly within pulmonary granulomas^31^. To characterize immune cell infiltration, we performed RareCyte Orion whole-slide multiplex immunofluorescence with custom-scripted image analysis of lung sections from WT and *Casp1*^*-/-*^*Casp11*^*-/-*^ mice infected with 10^2^ CFUs of LA. Granulomas from infected lung samples contained a central core of ruptured spherules surrounded by Ly6G⁺ H3Cit⁺ neutrophils, with an outer rim of CD3e⁺ T cells and smaller, intact spherules (Fig. 7D). At higher magnification, intact spherules were identified by the autofluorescence of the outer wall, whereas ruptured spherules displayed a discontinuous outer wall and infiltrating Ly6G⁺ H3Cit⁺ neutrophils (Fig. 7D, E). Quantitative analysis of granulomas showed increased numbers of CD11c⁺ resident macrophages in *Casp1*^*-/-*^*Casp11*^*-/-*^ mice compared with WT (Fig. 7E). *Casp1*^*-/-*^*Casp11*^*-/-*^ mice also had fewer FoxP3⁺ regulatory T cell numbers than WT, suggesting that caspase-1/11 signaling may indirectly promote the development or recruitment of regulatory T cells to the granuloma microenvironment (Fig. 7F).

To further investigate immune cell infiltration into ruptured spherules, we computationally generated a mask outlining individual spherules (solid white boundary). Nuclear staining revealed the presence of immune cells within ruptured spherules (Fig. 7G). These infiltrating cells included Ly6G⁺, H3Cit⁺, CD11c⁺, and F4/80⁺ populations, indicating the presence of neutrophils and neutrophil extracellular traps (NETs) formation, as well as resident macrophages (Fig. 7G). Quantification of the Ly6G⁺ area showed reduced neutrophil infiltration into ruptured spherules in caspase-1/11–deficient lungs; however, neutrophils still formed NETs, as indicated by H3Cit⁺ staining within ruptured spherules (Fig. 7G). In contrast, CD11c and F4/80 staining demonstrated increased accumulation of resident macrophages within ruptured spherules in caspase-1/11–deficient mouse lungs (Fig. 7G).

To further examine the role of alveolar macrophages (AVMs) in *Coccidioides* infections, AVMs were isolated from mouse lungs by bronchoalveolar lavage and infected with LA and FKA under spherule-inducing conditions. AVMs released IL-1β in response to spherule rupture (Fig. 7H). Loss of Dectin-1 and TLR2, NLRP3 and pyrin, or IL-18 significantly reduced IL-1β secretion (Fig. 7H). Spherule formation was markedly attenuated across all knockout genotypes (Fig. 7I). Notably, BMDMs deficient in Dectin-1 and TLR2, caspase-1/11, NLRP3 and pyrin, and GSDMD-GSDME also contained fewer spherules overall, consistent with a role for macrophage activation in promoting spherulation (Fig. 7J).

Multiplex imaging data also suggested that caspase-1/11 regulates neutrophil responses to *Coccidioides* spherules within the granuloma, as shown by an increase in H3Cit+ cells from *Casp1*^*-/-*^*Casp11*^*-/-*^ mice, including an increase in H3Cit⁺ cells within ruptured spherules (Fig. 7E, G). To explore this directly, bone marrow neutrophils (BMNs) were primed with Pam_2_CSK_4_ and challenged with FKS from the *C. immitis* RS and *C. posadasii* Silveira strains. BMNs produced IL-1β in response to *C. posadasii* but not *C. immitis*, indicating strain-specific neutrophil activation (Fig. 7K). Neutrophil IL-1β release was dependent on ASC, caspase-1, and both NLRP3 and pyrin inflammasomes. Combined deficiency of NLRP3 and pyrin, or GSDMD and GSDME, abolished IL-1β release (Fig. 7L). MLKL deficiency also abrogated IL-1β production, indicating nonredundant roles for gasdermins and MLKL in a signaling cascade initiated by the sensing of *Coccidioides* spherules (Fig. 7L). To assess the relative roles of Dectin-1 and TLR2, LPS was used to prime *Clec7a*^*-/-*^ or *Tlr2*^*-/-*^ BMNs before FKS challenge. IL-1β production depended primarily on Dectin-1 signaling (Fig. 7M). Taken together, these findings demonstrate that caspase-1/11-dependent inflammasome activation exacerbates disease severity without altering fungal burden, and that both macrophages and neutrophils require coordinated NLRP3-pyrin inflammasome signaling, gasdermin pore formation, and MLKL activity to generate IL-1β in response to *Coccidioides* spherules.

## Discussion

Coccidioidomycosis can present as either an acute or chronic pulmonary infection, typified by granulomatous lesions containing neutrophils and macrophages interacting with the spherule form of *Coccidioides*^32^. T cells also play critical roles in host defense, and T cell-deficiency or Th2-skewed responses are associated with susceptibility and poor prognosis^11^. All three stages of *Coccidioides* engage the host innate immune response, but each stage interacts with different innate immune cell types in the lung or in other organs following dissemination, potentially triggering stage-specific innate immune pathways due to the molecular and structural diversity of cell surface molecules displayed on *Coccidioides* morphotypes.

This study of the interaction between macrophages and the various developmental forms of *Coccidioides* emphasizes the complexity of the system. All three forms of the organism elicit different macrophage responses, and in turn, macrophages influence the differentiation of the organism. Arthroconidia can kill macrophages and stimulate the secretion of IL-1β via both the NLRP3 and pyrin inflammasome pathways. Macrophages also kill arthroconidia by the MLKL and ferroptotic pathways. Lower numbers of arthroconidia, especially in the presence of dead arthroconidia, are less toxic to macrophages, which then stimulate arthroconidia to differentiate into mature spherules that form endospores, the reproductive spherule form in vivo. These endospores, but not intact spherules, stimulate macrophages to produce IL-1. The macrophages influence spherule growth via mechanisms that include inflammasome and pyrin pathways. The presence of IL-18 appears to encourage spherule growth. *Casp1*^*-/-*^*Casp11*^*-/-*^ mice are less susceptible to infection with *Coccidioides* than controls, although the number of organisms is unchanged, emphasizing the role of the inflammasome in tissue injury.

The specific molecular pathways of the innate immune system required for host resistance to *Coccidioides* remain poorly understood. However, these pathways likely provide sufficient redundancy to counteract each morphotype during infection. Our current data extend the known roles of Dectin-1/CARD9 and TLR2/MyD88 signaling to include nonredundant roles for dual-sensing NLRP3 and pyrin inflammasomes, leading to IL-1β secretion via complementary actions of the pore-forming proteins GSDMD and GSDME (Supplementary Fig. 4). Remarkably, in neutrophils, MLKL appears to function within a linear signaling pathway with GSDMD and GSDME, and loss of either gasdermin or MLKL interferes with IL-1β production. In neutrophils undergoing necroptosis, loss of MLKL disrupts an array of ultrastructural cellular changes consistent with necroptosis, suggesting that MLKL acts as a signaling intermediary rather than solely as an endpoint effector in terminal membrane rupture^33^. These findings build on prior work demonstrating nonredundant activation of GSDMD and GSDME downstream of inflammasome activation^34^. Unexpectedly, gasdermins contribute only modestly to cell death in our system, suggesting the involvement of alternative, redundant pathways including Ninjurin-1 (NINJ1)-mediated membrane rupture^35^.

During initial respiratory exposure to infectious arthroconidia, our data suggest that ferroptotic signaling in macrophages contributes to host-mediated fungal damage and reduces arthroconidial viability. Although ferroptosis has not been linked to *Coccidioides* infection, inhibitors such as Ferrostatin-1 reduce the *Histoplasma capsulatum* burden in macrophages, and β-glucan from *Candida albicans* induces ferroptotic pathways in tumor-associated macrophages^36,37^. Macrophage death after exposure to arthroconidia is partially dependent on MLKL, but largely independent of pyroptosis. NINJ1 can mediate plasma membrane rupture during ferroptosis in macrophages independently of gasdermins and MLKL, and it is possible that NINJ1 contributes to ferroptotic cell death during *Coccidioides* infection^38^.

To dissect pathways responsible for IL-1β secretion and gasdermin-dependent pyroptosis, we compared macrophage responses to live and formalin-fixed “dead” arthroconidia. Although both states triggered macrophage death, co-exposure to live and dead arthroconidia suppressed cell death while strongly promoting differentiation into large, endosporulating spherules. Spherule formation does not require macrophages under certain in vitro conditions, but our experimental setup demonstrated an active bidirectional interaction between macrophages and *Coccidioides* that promotes spherule development^39,40^. We also discovered that dead arthroconidia trigger macrophages in a Dectin-1-independent manner, promoting the differentiation of arthroconidia into spherules rather than hyphae. Dectin-1/TLR2 and pyrin/NLRP3 appear to be required for normal spherule development, with respect to spherule size, and it is clear that Dectin-1-mediated signaling is essential for inflammasome activation and subsequent IL-1β release in response to spherules in macrophages and neutrophils. Our data suggest that the transition from arthroconidia to spherules is influenced by macrophage activation in response to dead arthroconidia, as spherules fail to form efficiently when dead arthroconidia are absent. The requirement for phagocytosis, phagolysosomal maturation, ROS, or enzymatic modifications of the arthroconidial lipid membrane remains unclear. We hypothesize that arthroconidia are phagocytosed before transitioning into spherules, but the molecular and transcriptomic changes during their release from macrophages, whether via exocytosis or cell death involving gasdermins or NINJ1, remain unknown.

The individual and complementary roles for NLRP3 and pyrin highlight multiple macrophage innate immune responses to *Coccidioides* infection. While NLRP3 activation is linked to diverse signals, pyrin is activated by bacterial inhibition of Rho GTPases and associated actin cytoskeletal changes^41^. Disruption of Wdr1 function, a cofilin cofactor required for actin depolymerization, also triggers pyrin activation, implicating actin cytoskeletal integrity in inflammasome signaling^42^. Pyrin inflammasome activation resembles the “guard hypothesis” in plants, in which immune responses are initiated by pathogen virulence factors that interfere with host cellular processes. Pyrin co-localizes with ASC at sites of actin polymerization during cell chemotaxis^43^. Gain-of-function mutations in Rac2, a Rho GTPase, promote NLRP3 inflammasome activation via PAK1 kinase^44^. Other Rho GTPases, including CDC42, play key roles in pyrin inflammasome formation. Both WT CDC42 and catalytically inactive CDC42 are capable of promoting pyrin activation, emphasizing the interplay between actin dynamics and innate immunity^44^. The precise molecular mechanism by which pyrin is activated by arthroconidia or spherules remains unknown; however, extensive cytoskeletal remodeling likely contributes to innate sensing during *Coccidioides* infection, particularly in response to the larger morphologies of *Coccidioides*, i.e., mature spherules.

Optimizing methods for macrophage-based spherule culture systems has enabled a detailed analysis of the roles of host innate immune pathways in fungal morphogenesis. Deficiencies in NLRP3 and pyrin impair spherule growth, but not total number, and the combined loss of pyrin and NLRP3 exacerbates this defect. IL-1β is unlikely to be the major signal promoting spherule differentiation, since it is detected only in supernatants after spherule rupture. Given the asynchronous nature of spherule maturation in culture, it is possible that macrophage autocrine or paracrine signaling by IL-1β or IL-18, mediated by the NLRP3/pyrin inflammasomes, may serve as a macrophage priming step that promotes spherule growth. In this assay system, IL-18 is an important cytokine for spherule development and growth. Others have reported that IL-18 receptor deficiency on the sensitive C57BL/6 J background did not increase susceptibility to *Coccidioides* infection, but infected *IL18r1*^*-/-*^ mice had lower levels of TNF, IL-10, and IFN-γ in the bronchoalveolar lavage fluid ^15^. Additionally, dead arthroconidia may serve as a priming signal for inflammasome activation, resulting in enhanced IL-1β or IL-18 secretion upon exposure to fixed arthroconidia or spherules. Further molecular studies are needed to clarify how dead arthroconidia and IL-1β/IL-18 influence acute inflammation and fungal morphogenesis.

Granulomas are thought to constrain fungal virulence within a localized region of the lung. Understanding the mechanisms behind the coordinated participation of myeloid and T cell populations that make up a coccidioidal granuloma can shed light on the signals that allow chronic inflammation and dissemination, and that dictate patient outcome. Our data further indicate that caspase-1/11 shapes granuloma-associated responses in the lung by increasing neutrophil recruitment to rupturing spherules while moderating the formation of NETs within the necrotizing coccidioidal granuloma core—a feature of neutrophil activation that is also detected in caseating granulomas in tuberculosis^45–47^. Coccidioidal granulomas also contain Tregs, and caspase-1/11-deficiency reduces the numbers of Tregs compared to infected WT controls. Further investigation is needed to understand how innate immune pathways such as NLRP3-pyrin inflammasomes influence the spatial behavior of innate and adaptive immune cells to *Coccidioides* morphotypes, and around the coccidioidal granuloma, to clarify the events underlying fungal migration and dissemination from the granuloma core to the periphery.

## Method

### Mice

All animal experiments complied with and were approved by the UCSD Institutional Animal Care and Use Committee. All mice were on a C57BL/6J genetic background and were between 8 and 20 weeks of age. Both male and female mice were used. The *Clec7a*^*-/-*^ (JAX stock #012337), *Tlr2*^*-/-*^ (JAX stock #004650), *Gsdmd*^*-/-*^ (JAX stock #032410), *Gsdme*^*-/-*^ (JAX stock #032411), *Casp1*^*-/-*^ (JAX stock #016621)*, Casp1*^*-/-*^*Casp11*^*-/-*^ (JAX stock #016621), *Il18*^*-/-*^ (JAX stock #004130) mouse strains were generated on or backcrossed at least 10 generations. *Nlrp3*^*-/-*^ and *Asc*^*-/-*^ mouse strains^48,49^, and *Mlkl*^*-/-*^ mouse strains^50^ were generated as previously described. *Mefv*^*-/-*^ mice were generated by Jae Jin Chae and generously provided by Youssef Aachoui^51^. *Il1b*^*-/-*^ mice were generated and generously provided by David D. Chaplin^52^.

### Macrophage culture

Bone marrow-derived macrophages (BMDMs) were derived from 10^7^ bone marrow cells in Dulbecco’s Modified Eagle’s Medium (DMEM; Gibco #10566024), supplemented with 10% fetal bovine serum (FBS; Sigma-Aldrich #F0926) and penicillin/streptomycin (P/S; Gibco #150122). Cells were differentiated in the presence of 100 ng/mL macrophage colony-stimulating factor (M-CSF; Biolegend #576404) for 5 days at 37 °C with 10% CO_2_. Bone marrow cells were initially cultured on tissue culture (TC)-treated flasks for 16 h, after which non-adherent cells were transferred to non-TC-treated Petri dishes and cultured for an additional 4 days. Following differentiation, BMDM were harvested, counted, and replated onto TC-treated plates in DMEM supplemented with 10% FBS, 100 ng/mL M-CSF, and 20 ng/mL granulocyte-macrophage colony-stimulating factor (GM-CSF; Biolegend #576306). Cells were incubated overnight at 37 °C in 10% CO_2_ prior to downstream assays.

### Fungal strain and arthroconidia harvest

All live *Coccidioides* experiments were performed in a biosafety level 3 (BSL3) facility. The *Coccidioides* fungal strains *C. immitis* RS and *C. posadasii* Silveira were obtained from BEI (RS NR-48942; Silveira NR-48944). Frozen stocks were cultured on 2X Glucose Yeast Extract (2X GYE; 2% glucose Sigma-Aldrich, #G8270-5KG; 1% yeast extract, Gibco #DF210929) agar (Gibco #DF0145-17-0) supplemented with penicillin and streptomycin (2XGYE+P/S) plates and incubated for 4–6 weeks at 30 °C to allow arthroconidia formation. Arthroconidia were harvested as described previously^40^. Harvested arthroconidia stocks were stored at 4 °C, and their viability was assessed periodically by plating serial dilutions on 2X GYE+P/S agar plates. Colony-forming units (CFUs) were enumerated after 4 days of incubation at 30 °C. For spherule preparation in the absence of BMDMs, arthroconidia were inoculated into liquid Converse medium at a density of 10^6^ arthroconidia/mL density and incubated for 4 days at 39 °C with 10% CO_2_ under shaking (150 rpm)^40^. Formalin-killed arthroconidia (FKA) or formalin-killed spherules (FKS) stocks were prepared by resuspending live arthroconidia or spherules in 5% formaldehyde (Sigma-Aldrich #252549-500ML) in PBS (Gibco #10010049) for 20 min. After confirming loss of viability on 2X GYE+P/S agar plates, the suspension was washed 3 times and resuspended in PBS for long-term storage. To assess fungal viability following ferric citrate (FAC, Sigma-Aldrich #F3388) treatment, arthroconidia stocks were diluted accordingly and plated onto 2X GYE+P/S agar plates supplemented with FAC at 50, 100, 200, and 500 µM. CFUs were enumerated after incubation for 4 days at 30 °C.

### Macrophage infections and live-cell imaging

BMDMs were seeded at 5 × 10^4^ cells per well in 96-well TC-treated plates (Corning #3904). Where indicated, BMDMs were primed with 100 ng/mL lipopolysaccharide (LPS; *Escherichia coli* Serotype O55:B5, Enzo Life Sciences #ALX-581-013) for 4 h, followed by 15 µM nigericin (Invivogen #tlrl-nig) for 30 min. *Coccidioides* inoculum at a multiplicity of infection (MOI) of 0.01 to 5 was prepared from fresh arthroconidia stocks or stocks that were formalin-fixed as described above. Viability of BMDMs was assessed with 1 µg/mL propidium iodide (PI; Sigma-Aldrich #P4170) using four-tile standard or whole-well scans using the G/O/NIR Optical Module on the Incucyte SX5. Individual wells were scanned every 4–8 h over a span of 4 days at 37 °C with 5% CO_2_. Analysis was completed with the Basic Analyzer Optical Module software (Sartorius) with a top-hat segmentation radius of 100 µm, and an Orange Mean Intensity threshold of 2. To assess fungal proliferation in co-cultures with BMDMs, supernatants were collected, and the remaining BMDMs were lysed with water. The lysates were then combined with the culture supernatants and plated onto 2X GYE agar plates supplemented with FAC, 20 µM docosahexaenoic acid (DHA, Cayman Chemicals #90310), 10 µM liproxstatin-1 (Lip-1, Cayman Chemicals #17730), or PBS as a control. CFUs were enumerated after 4 days of incubation at 30 °C. β-glucan (Invivogen #tlr-bgp) was added to BMDM cultures at 25 and 50 µg/mL at 48 h post-infection. To assess spherule growth, human recombinant IL-18 binding protein (provided by AB2Bio) was added to BMDM cultures at doses previously shown to block mouse IL-18 signaling and incubated for 1 h at 37 °C with 5% CO_2_.

### Growing spherules and endospores harvest

Spherules were generated by co-culturing BMDMs with live arthroconidia at MOI:0.01 and FKA at MOI:0.1. Cultures were incubated for 4 days at 37 °C with 10% CO_2_ and monitored using the Incucyte SX5 (Sartorius). When mature spherules burst, the culture supernatant containing live endospores (LE) was collected and filtered through a 40 µm cell strainer followed by a 5 µm cell strainer. The resulting endospore suspension was washed twice and resuspended in PBS.

### Flow cytometry and ELISA

BMDMs were stained with anti-mouse CD45 conjugated to APC-Cy7 (Biolegend #103116, 1:400) and 1 µg/mL PI at 4 °C for 15 min. Flow cytometry was performed on an LSR Fortessa (BD Biosciences) and analyzed with FlowJo (v10.9.0). ELISAs for IL-1β (R&D Systems #DY401) and TNF (R&D Systems #DY410) were performed according to the manufacturer’s instructions.

### Quantification of spherules by Fungal Finder

For detailed quantification of spherule area and count in BMDM cultures, Fungal Finder (FGF), a custom macro was developed^53^. The macro leveraged a Cellpose model trained in Fiji from scratch on a diverse range of images representing the various types of spherules and background encountered under experimental conditions^54^. The macro ran the custom-trained model and used the resulting segmentation to measure the area of each spherule in an image. Data were recorded for both the average spherule area per well and individual spherules. An overview of the FGF workflow is provided in Supplementary Fig. 3. All original macro files, example images, and models have been deposited on Figshare (https://figshare.com/s/daa464a27cbaaa811c0b) and are publicly available at 10.26180/28702103 as of the date of publication.

### Electron microscopy

Cells were fixed with 2.5% glutaraldehyde (Electron Microscopy Sciences #16221) in 0.1 M sodium cacodylate buffer (Electron Microscopy Sciences #12310), pH 7.4, for 2 h on ice, then embedded in 10% gelatin (Knox Original Gelatin). After washing with 0.1 M cacodylate buffer, the pellets were post-fixed in 1% OsO_4_ (Electron Microscopy Sciences #19151) in 0.1 M cacodylate buffer for 1 h on ice. The cells were stained with 2% uranyl acetate (Ladd Research #23620) for 1 h on ice, then dehydrated through a graded ethanol series (50–100%; Sigma-Aldrich #E7023), washed in acetone (Fisher Chemical #A949-4), and embedded with Durcupan (Sigma-Aldrich #44610-1EA). Sections were cut at 60 nm on a Leica UCT ultramicrotome and placed on 300 mesh copper grids. Sections were post-stained with 2% uranyl acetate for 5 min and Sato’s lead stain (1% Lead Nitrate, Electron Microscopy Sciences #17900; 1% Lead Citrate, Electron Microscopy Sciences #17800; 1% Lead Acetate, Electron Microscopy Sciences #17600; 2% Sodium Citrate, LabChem LC236501) for 1 min. Images were acquired with a JEOL 1400 plus transmission electron microscope, operated at 80 KeV and equipped with a bottom-mounted 4kx4k camera, Gatan One View.

### Mouse infections and quantification of fungal burden in vivo

All mouse infections were performed in an animal BSL3 facility. Male and female C57BL/6J mice, aged 8–12 weeks, were anesthetized with 3–5% isoflurane (5 L per min) and oxygen (2 L per min). Mice were intranasally infected with 50 μL of PBS containing 10^2^ live and/or 10^4^ dead *C. immitis* arthroconidia. To assess fungal burden, lungs and spleens were harvested post-euthanasia and homogenized in 1 mL of PBS for 10 min at a frequency of 300 per second using 5 mm stainless steel beads (Qiagen #69989) in a TissueLyser II (Qiagen). Serial dilutions of the homogenates were plated onto 2XGYE+P/S. CFUs were enumerated after incubation for 3 days at 30 °C.

### Immunostaining and microscopy

Lungs from infected mice were harvested post-euthanasia and fixed in 10% buffered formalin (Sigma-Aldrich #Z202). Lungs were then embedded in paraffin wax, sectioned at 5 µm, stained with hematoxylin and eosin, or subjected to multiplex immunofluorescence. For immunofluorescence, antigen retrieval was performed in eBioscience High pH Solution (Invitrogen #00-4956-58) using a Biocare Medical Decloaking Chamber™ NxGen (#DC2012) at 110 °C for 15 min, and then cooled to room temperature. Autofluorescence was quenched in PBS with 2.4 mM sodium hydroxide and 1.47 M hydrogen peroxide solution under light-emitting diode or 1 h and ultraviolet light for 30 min. Slides were blocked with Image-iT FX Signal Enhancer (Invitrogen #I36933) for 15 min and stained with a conjugated antibody cocktail containing Cit-Histone3 (EPR20358-13, ArgoFluor660L, Abcam #ab232939), CD8a (D4W2Z, ArgoFluor572), Ly6G (E6Z1T, ArgoFluor602, Cell Signaling Technology #75028SF), CD11c (D1V9Y, ArgoFluor624), F4-80 (D2D9R, ArgoFluor662), CD3e (D4V8L, ArgoFluor686), FOXP3 (D698R, Digoxigenin), CD4 (4SM95, ArgoFluor784, Cell Signaling Technology #27520SF), and CD31 (D8V9E, ArgoFluor874, Cell Signaling Technology #92841SF) in Antibody Stabilizer PBS with 10% mouse/rabbit serum overnight at 4 °C. The next day, slides were stained with Anti Digoxigenin (HY-A.1, ArgoFluor760, RareCyte #52-1072-702) in Antibody Stabilizer PBS with 10% goat serum for 30 min, counterstained with Hoechst at 1:1000 for 5 min, coverslipped with ArgoFluor Mounting Medium, and dried for 48 h before scanning.

Images were acquired with a 20× 0.75 dry objective (0.325μm/pixel) using whole-tissue tiling. The Orion system (RareCyte, Seattle, Washington, USA) captured approximately 10 nm emission bands via angled dichroic mirrors. RareCyte’s algorithm for tile stitching, non-linear channel alignment, and spectral unmixing based on single-color controls was used to process the images. Two autofluorescence channels were used to visualize general tissue structure and *Coccidioides*.

### Orion image analysis

Multiplex images were analyzed in QuPath^55^. First, cells were segmented with InstanSeg^56^. Random-forest object classifiers were trained to classify cells as positive or negative for CD3, CD4, CD8, FoxP3, H3Cit, F4/80, and CD11c, independently. Additionally, a pixel classifier was trained to identify the gross granuloma region using the Hoechst, autofluorescence 1 (tissue structure), autofluorescence 2 (*Coccidioides* spherule wall), F4/80, CD11b, and CD31 channels. The same pixel- and object-classifiers were applied to all images. Within the granulomas, the frequencies of various cell subsets were calculated. Masks were annotated in QuPath by tracing the boundary of the spherule wall.

### Isolation of mouse alveolar macrophages

Alveolar macrophages (AVMs) were isolated from mouse lungs by bronchoalveolar lavage. Briefly, mice were euthanized with ketamine-xylazine, the trachea was cannulated, and the lungs were lavaged with sterile, warm PBS/1% FBS (Sigma-Aldrich #F0926). Lavage fluid was collected and centrifuged at 400 g for 10 min to pellet cells. After counting, AVMs were seeded at a density of 2 × 10^4^ cells per well in 96-well TC-treated plates in DMEM supplemented with 10% FBS, 100 ng/mL M-CSF, and 20 ng/mL granulocyte-macrophage colony-stimulating factor, then incubated for 2 h at 37 °C with 10% CO_2_. Non-adherent cells were then removed. Viability of AVMs was monitored with 1 µg/mL PI in whole-well scans using the G/O/NIR Optical Module on the Incucyte SX5. Individual wells were scanned every 4–8 h over 3 days at 37 °C with 5% CO_2_. Analysis was completed with the Basic Analyzer Optical Module software using a top-hat segmentation radius of 100 μm, and an Orange Mean Intensity threshold of 2.

### Purification of mouse bone marrow neutrophils

Bone marrow neutrophils (BMNs) were isolated from bone marrow cells in Hanks’ balanced salt solution (HBSS; Gibco #14175145) supplemented with 1% FBS and 15 mM ethylenediaminetetraacetic acid (EDTA; Promega #H5032). Cells were overlaid onto 68%, 78%, and 52% Percoll Plus (Cytiva #45001753) layers, and centrifuged at 400 × *g* for 30 min at 4 °C. Neutrophils were isolated from the 68/78% layer and added to TC-treated plates in DMEM supplemented with 10% FBS, 20 ng/mL interferon gamma (IFN-γ; Biolegend #575306), and 100 ng/mL granulocyte colony-stimulating factor (GCSF; Amgen NEUPOGEN®). Plates were incubated for 90 min at 37 °C in 10% CO_2_ before the assay. BMNs were primed for 4 h with 100 ng/mL LPS or 100 ng/mL Pam_2_CysSerLys_4_ (Invivogen #tlrl-pm2s-1) before FKS exposure.

### Statistical analyses and software

Data are presented as mean ± standard error of the mean (SEM). Comparisons were performed using a Student’s t-test or ANOVA with a Tukey multiple comparison test. All statistical analyses were performed using Prism software (GraphPad v10.4.1). All graphs and figures were formatted in Adobe Illustrator (v28.1).

### Reporting summary

Further information on research design is available in the Nature Portfolio Reporting Summary linked to this article.

## Supplementary information

Supplementary Information

Description of Additional Supplementary Files

Movie S1

Movie S2

Movie S3

Reporting Summary

Transparent Peer Review file

## Source data

Source data

## Data Availability

All materials and reagents generated in this study are available upon request from the lead contact. Source data are provided with this paper. A copy of the FGF code, example images, and the Cellpose models have been deposited on Figshare and are accessible at https://figshare.com/s/daa464a27cbaaa811c0b as of the date of publication. Further information is available upon request from the lead contact.
