# Supplementary material for: Dual activation of NLRP3 and pyrin inflammasomes mediates host responses to the human fungal pathogen Coccidioides
Source: Nat Commun. 2026 Jul 1;17:8209. doi: 10.1038/s41467-026-75022-8 (PMC13462806; doi:10.1038/s41467-026-75022-8)
Supplement: Supplementary file 1 [file 41467_2026_75022_MOESM1_ESM.pdf]

Title:

Dual Activation of NLRP3 and Pyrin Inflammasomes Mediates Host Responses to the Human Fungal Pathogen *Coccidioides*

Supplementary Information File

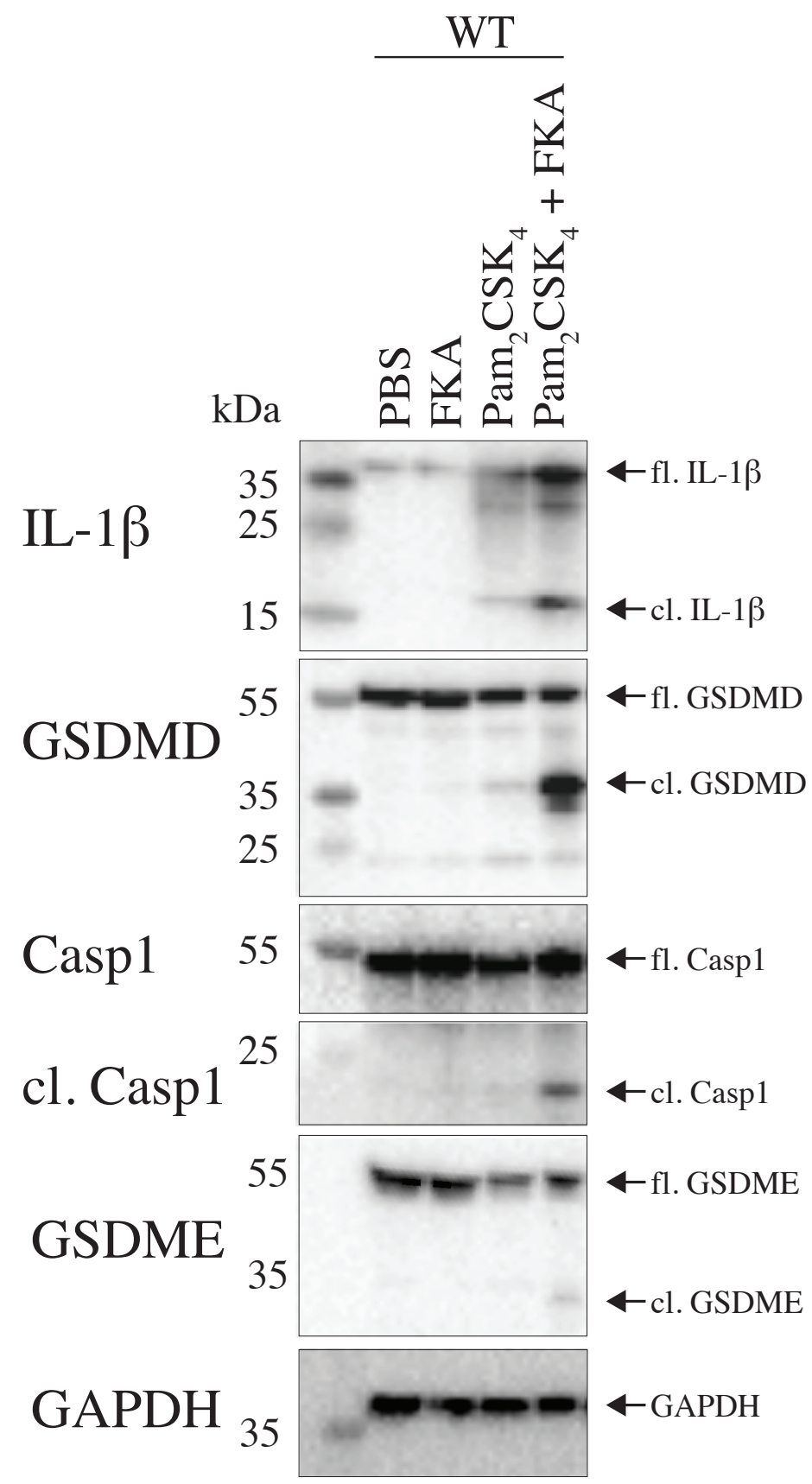

Supplementary Figure 1: Dead arthroconidia-infected macrophages require PAM_2CSK_4 priming for cleavage of IL-1B, Caspase-1, GSDMD and GSDME. Immunoblot analysis of WT bone marrow-derived macrophages (BMDMs) treated with formalin-killed arthroconidia (FKA) at MOI:1, with or without PAM_2CSK_4 priming. Full-length and cleaved IL-1β, GSDMD, Caspase-1, GSDME, and GAPDH of cell lysates were analyzed by immunoblot. Uncropped blots are provided at the end of the Supplementary Information File.

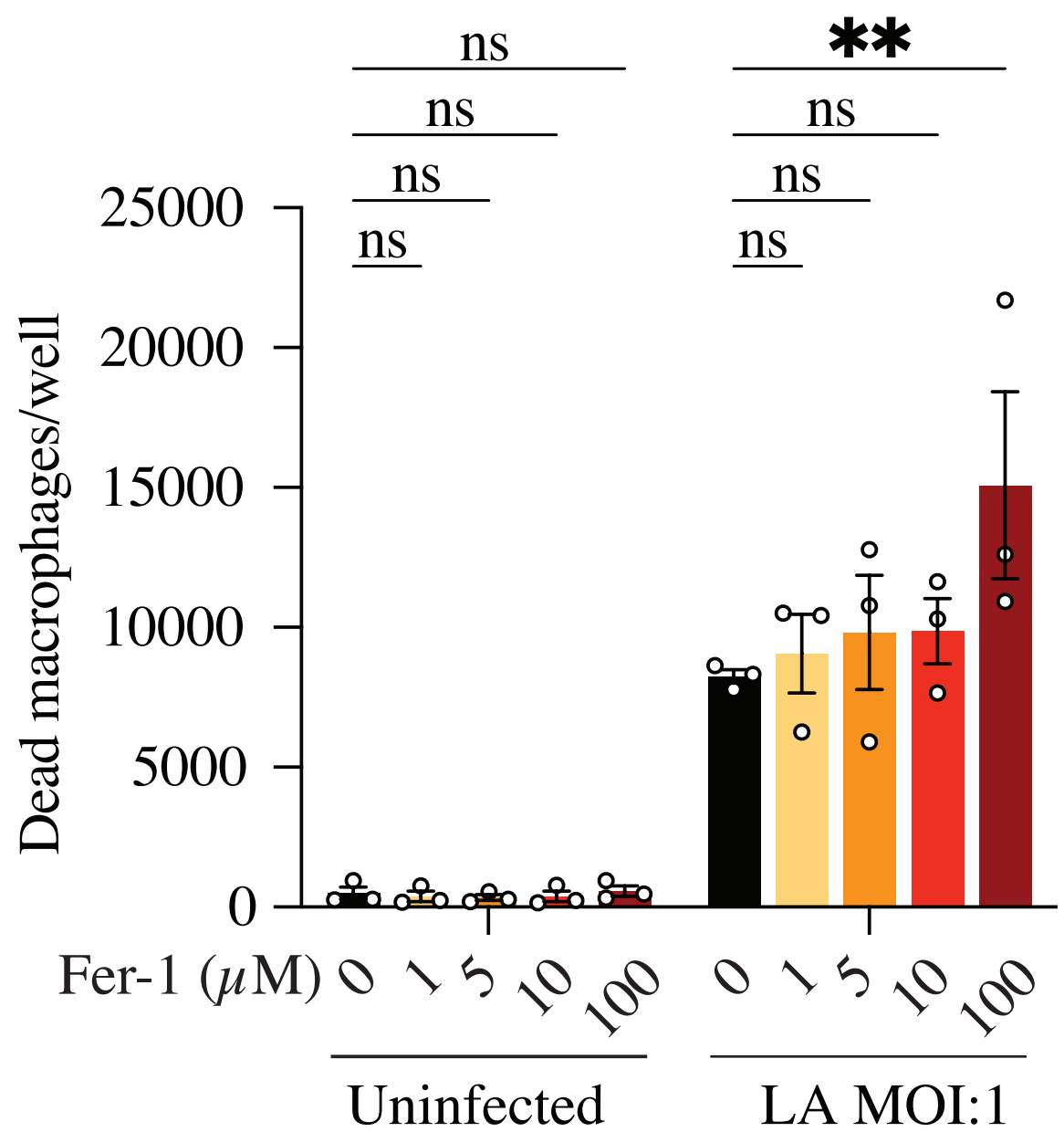

Supplementary Figure 2: Inhibition of ferroptotic signaling by ferrostatin-1 increase live arthroconidia-induced macrophage death. Cell viability by live-cell imaging of live arthroconidia (LA) -infected WT (n = 3) BMDMs at MOI:1, and treated with ferrostatin-1 (Fer-1) at 1, 5, 10, 100 μM. Mean ± SEM. Each data point represents a biological replicate. WT vs knockouts, two-way ANOVA, $^{**}P < 0.005$, ns: not significant.

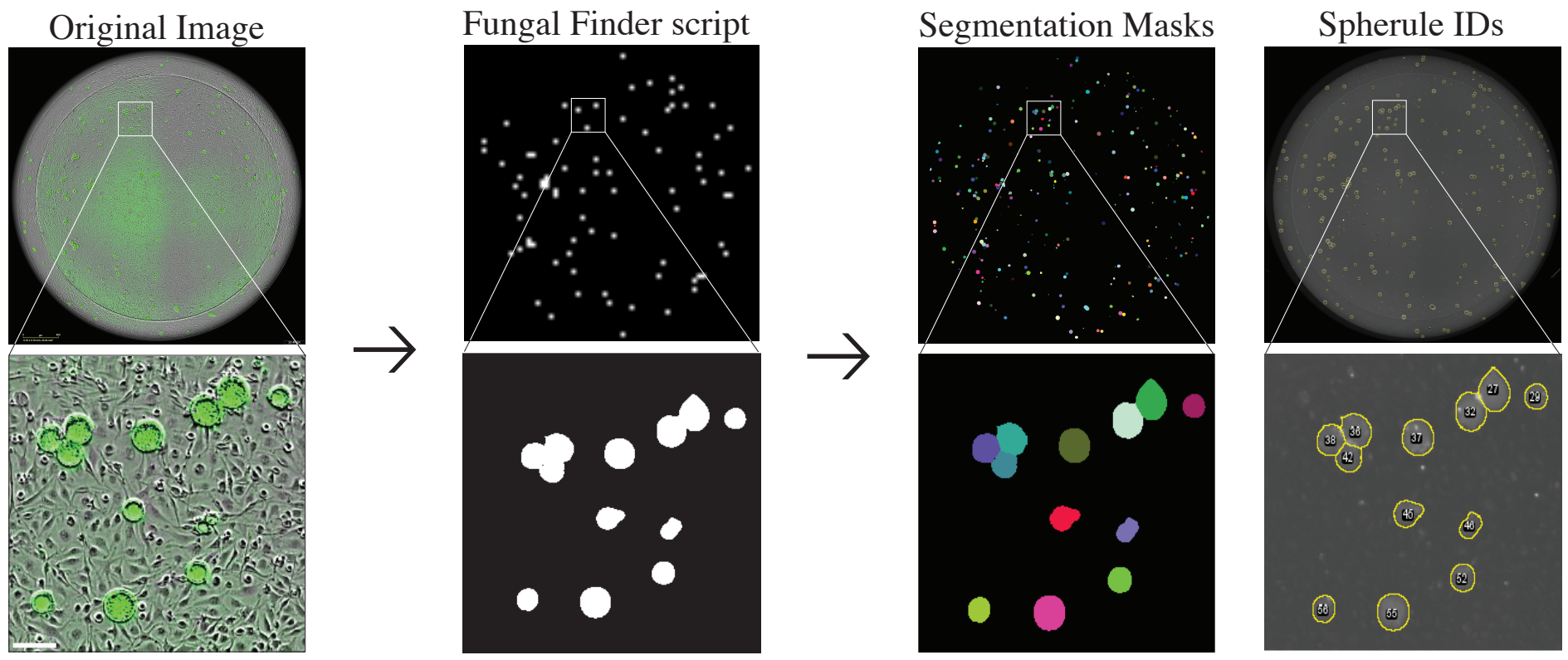

Supplementary Figure 3: Fungal Finder workflow. Whole-well stills from live-cell imaging experiments are captured using the Incucyte SX5. A Cellpose model identifies spherules based on the autofluorescence of their outer walls. Individual spherules are outlined with a segmentation mask and labeled with a numeric ID. Top panel: uncropped still of a whole-well scan. Bottom panel: zoomed-in region of the top panel.

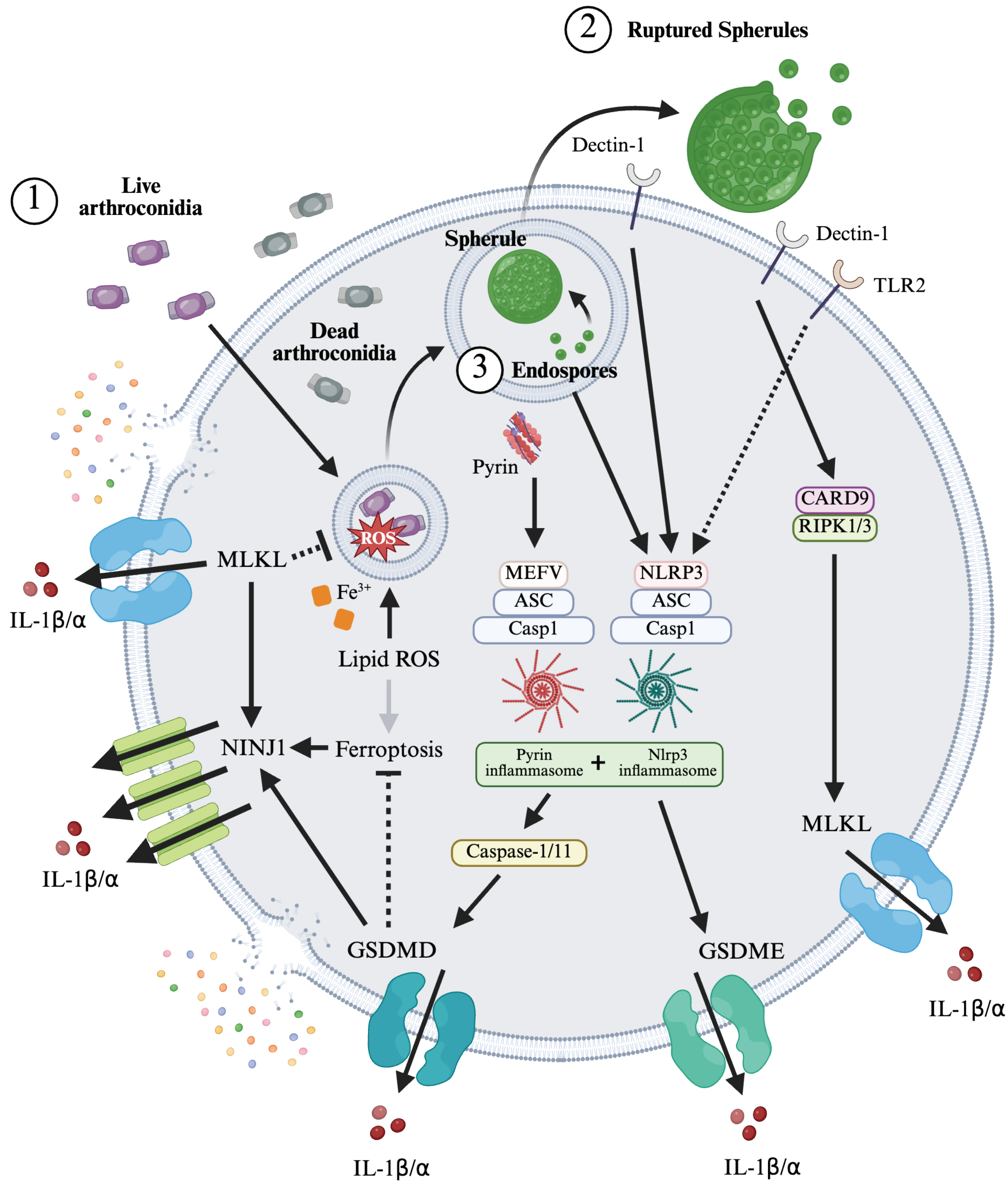

Supplementary Figure 4: Schematic summary of host-*Coccidioides* interactions. The three distinct morphologies of *Coccidioides* **(1)** live arthroconidia, **(2)** ruptured spherules, and **(3)** endospores initiate dual inflammasome activation and regulated cell death. Schematic was created using BioRender.com.

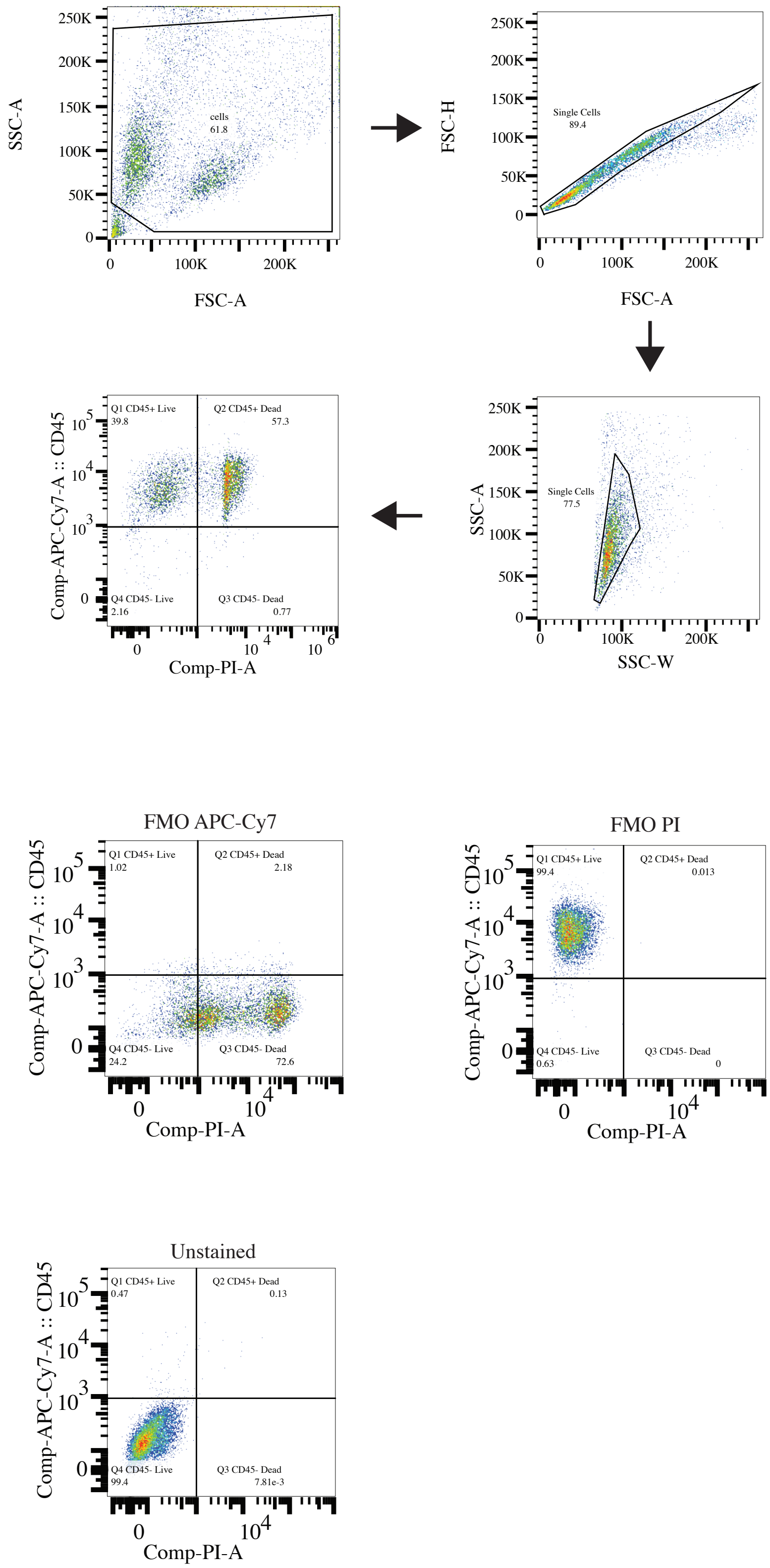

Supplementary Figure 5: Gating strategy used in flow cytometry analysis experiment. BMDMs were first gated based on FSC-A versus SSC-A to identify the main cell population. Doublets were excluded using FSC-A versus FSC-H and SSC-A versus SSC-W. Live BMDMs were defined as CD45+ Dead (PI+) cells. Fluorescence minus one (FMO) and unstained controls were used to establish gating boundaries.

Supplementary Movies

Supplementary Movie 1: Live arthroconidia induces macrophage death in a MOI-dependent manner. A time-lapse live-cell imaging of uninfected and LA-infected WT BMDMs at MOI:0.5, 1, 2, 5. Cell viability was assessed with 1 µg/mL propidium iodide. Images were acquired every 2 h for 20 h. Representative images from three independent biological replicates are shown. Scale bar = 200 µm.

Supplementary Movie 2: Dead arthroconidia enhances spherule formation by live arthroconidia in BMDMs. A time-lapse live-cell imaging of WT BMDMs infected with LA MOI:0.01 and FKA MOI:1. Green autofluorescence outlines the spherule wall. Images were acquired every 8 h for 64 h. Representative images from three independent biological replicates are shown. Scale bar = 200 µm.

Supplementary Movie 3: Endospores transition rapidly to spherules in BMDM cultures. A time-lapse live-cell imaging of WT BMDMs infected with live endospores at MOI:0.01. Green autofluorescence outlines the spherule wall. Images were acquired every 4 h for 40 h. Representative images from three independent biological replicates are shown. Scale bar = 100 µm.

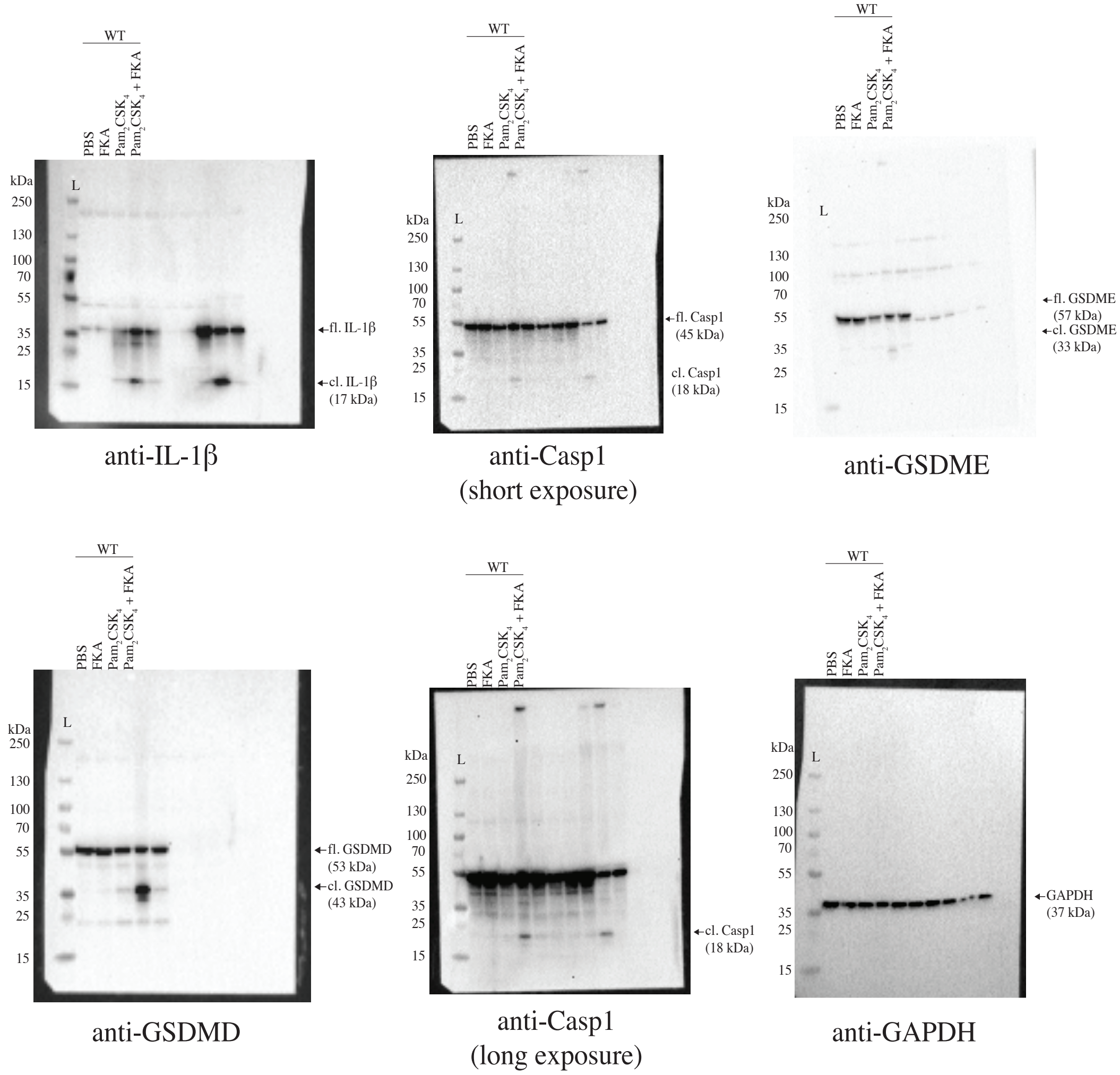

Uncropped blots of Supplementary Figure 1.
