# Supplementary material for: Dual activation of NLRP3 and pyrin inflammasomes mediates host responses to the human fungal pathogen Coccidioides
Source: Nat Commun. 2026 Jul 1;17:8209. doi: 10.1038/s41467-026-75022-8 (PMC13462806; doi:10.1038/s41467-026-75022-8)
Supplement: Supplementary file 2 [file 41467_2026_75022_MOESM2_ESM.pdf]

Title: Supplementary Movie 1

Description: Live arthroconidia induces macrophage death in a MOI-dependent manner. A time-lapse live-cell imaging of uninfected and LA-infected WT BMDMs at MOI:0.5, 1, 2, 5. Cell viability was assessed with 1 μg/mL propidium iodide. Images were acquired every 2 h for 20 h. Representative images from three independent biological replicates are shown. Scale bar = 200 μm.

Title: Supplementary Movie 2

Description: Dead arthroconidia enhances spherule formation by live arthroconidia in BMDMs. A time-lapse live-cell imaging of WT BMDMs infected with LA MOI:0.01 and FKA MOI:1. Green autofluorescence outlines the spherule wall. Images were acquired every 8 h for 64 h. Representative images from three independent biological replicates are shown. Scale bar = 200 μm.

Title: Supplementary Movie 3

Description: Endospores transition rapidly to spherules in BMDM cultures. A time-lapse live-cell imaging of WT BMDMs infected with live endospores at MOI:0.01. Green autofluorescence outlines the spherule wall. Images were acquired every 4 h for 40 h. Representative images from three independent biological replicates are shown. Scale bar = 100 μm
