# Supplementary material for: Dual activation of NLRP3 and pyrin inflammasomes mediates host responses to the human fungal pathogen Coccidioides
Source: Nat Commun. 2026 Jul 1;17:8209. doi: 10.1038/s41467-026-75022-8 (PMC13462806; doi:10.1038/s41467-026-75022-8)
Supplement: Supplementary file 3 [file 41467_2026_75022_MOESM6_ESM.pdf]

nature portfolio

Corresponding author(s): Sinem Beyhan

Last updated by author(s): 05/06/2026

Reporting Summary

Nature Portfolio wishes to improve the reproducibility of the work that we publish. This form provides structure for consistency and transparency in reporting. For further information on Nature Portfolio policies, see our Editorial Policies and the Editorial Policy Checklist.

Statistics

For all statistical analyses, confirm that the following items are present in the figure legend, table legend, main text, or Methods section.

n/a	Confirmed	
☐	☒	The exact sample size (*n*) for each experimental group/condition, given as a discrete number and unit of measurement
☐	☒	A statement on whether measurements were taken from distinct samples or whether the same sample was measured repeatedly
☐	☒	The statistical test(s) used AND whether they are one- or two-sided<br>*Only common tests should be described solely by name; describe more complex techniques in the Methods section.*
☐	☒	A description of all covariates tested
☒	☐	A description of any assumptions or corrections, such as tests of normality and adjustment for multiple comparisons
☐	☒	A full description of the statistical parameters including central tendency (e.g. means) or other basic estimates (e.g. regression coefficient) AND variation (e.g. standard deviation) or associated estimates of uncertainty (e.g. confidence intervals)
☐	☒	For null hypothesis testing, the test statistic (e.g. *F*, *t*, *r*) with confidence intervals, effect sizes, degrees of freedom and *P* value noted<br>*Give P values as exact values whenever suitable.*
☒	☐	For Bayesian analysis, information on the choice of priors and Markov chain Monte Carlo settings
☒	☐	For hierarchical and complex designs, identification of the appropriate level for tests and full reporting of outcomes
☒	☐	Estimates of effect sizes (e.g. Cohen's *d*, Pearson's *r*), indicating how they were calculated

Our web collection on statistics for biologists contains articles on many of the points above.

Software and code

Policy information about availability of computer code

Data collection	N/A
Data analysis	For detailed quantification of spherule area and count in BMDM cultures, Fungal Finder (FGF), a custom macro was developed. The macro leveraged a Cellpose model trained in Fiji from scratch on a diverse range of images representing the various types of spherules and background encountered under experimental conditions. The macro ran the custom-trained model and used the resulting segmentation to measure the area of each spherule in an image. Data were recorded for both the average spherule area per well and individual spherules. An overview of the FGF workflow is provided in Supplementary Figure 1. All original macro files, example images, and models have been deposited on Figshare (https://figshare.com/s/daa464a27cbaaa811c0b), and are publicly available at https://www.doi.org/10.26180/28702103 as of the date of publication.

For manuscripts utilizing custom algorithms or software that are central to the research but not yet described in published literature, software must be made available to editors and reviewers. We strongly encourage code deposition in a community repository (e.g. GitHub). See the Nature Portfolio guidelines for submitting code & software for further information.

Data

Policy information about availability of data

All manuscripts must include a data availability statement. This statement should provide the following information, where applicable:

- Accession codes, unique identifiers, or web links for publicly available datasets
- A description of any restrictions on data availability
- For clinical datasets or third party data, please ensure that the statement adheres to our policy

All data, materials, and reagents generated in this study are available upon request from the lead contact. A copy of the FGF code, example images, and the Cellpose models have been deposited on Figshare and is accessible at https://figshare.com/s/daa464a27cbaaa811c0b as of date of publication. Further information is available upon request from the lead contact.

Research involving human participants, their data, or biological material

Policy information about studies with human participants or human data. See also policy information about sex, gender (identity/presentation), and sexual orientation and race, ethnicity and racism.

Reporting on sex and gender	N/A
Reporting on race, ethnicity, or other socially relevant groupings	N/A
Population characteristics	N/A
Recruitment	N/A
Ethics oversight	N/A

Note that full information on the approval of the study protocol must also be provided in the manuscript.

Field-specific reporting

Please select the one below that is the best fit for your research. If you are not sure, read the appropriate sections before making your selection.

[x] Life sciences [ ] Behavioural & social sciences [ ] Ecological, evolutionary & environmental sciences

For a reference copy of the document with all sections, see nature.com/documents/nr-reporting-summary-flat.pdf

Life sciences study design

All studies must disclose on these points even when the disclosure is negative.

Sample size	N/A
Data exclusions	N/A
Replication	All experiments were repeated with at least three biological replicates, and with at least four technical replicate for each biological replicate. Mouse experiments were performed with at least 5 mice per genotype per group for experiment.
Randomization	N/A
Blinding	N/A

Reporting for specific materials, systems and methods

We require information from authors about some types of materials, experimental systems and methods used in many studies. Here, indicate whether each material, system or method listed is relevant to your study. If you are not sure if a list item applies to your research, read the appropriate section before selecting a response.

Materials & experimental systems

n/a	Involved in the study
☐	☒ Antibodies
☒	☐ Eukaryotic cell lines
☒	☐ Palaeontology and archaeology
☐	☒ Animals and other organisms
☒	☐ Clinical data
☒	☐ Dual use research of concern
☒	☐ Plants

Methods

n/a	Involved in the study
☒	☐ ChIP-seq
☒	☐ Flow cytometry
☒	☐ MRI-based neuroimaging

Antibodies

Antibodies used	anti-CD45 Antibody (Biolegend #103115; 30-F11), anti-Cit-Histone3 (Abcam #ab232939; EPR20358-13), anti-CD8a (RareCyte #53-1003-501; D4W2Z), anti-Ly6G (Cell Signaling Technology #75028SF;E6Z1T), anti-CD11c (RareCyte #53-1004-601; D1V9Y), anti-F4-80 (RareCyte #53-1024-601; D2D9R), anti-CD3e (RareCyte #53-1001-601; D4V8L), anti-FOXP3 (RareCyte #53-1010-101; D698R), anti-CD4 (Cell Signaling Technology #27520SF;4SM95), anti-CD31 (Cell Signaling Technology #92841SF;D8V9E).
Validation	Validated by commercial vendors.

Animals and other research organisms

Policy information about studies involving animals; ARRIVE guidelines recommended for reporting animal research, and Sex and Gender in Research

Laboratory animals	Mouse strains were generated on, or had been backcrossed at least 10 generations with, the C57BL/6J background. Both males and females were utilized for analysis, and were less than 1 year age
Wild animals	N/A
Reporting on sex	N/A
Field-collected samples	N/A
Ethics oversight	All animal experiments complied with the regulatory standards of, and were approved by, the Institutional Animal Care and Use Committees at University of California, San Diego.

Note that full information on the approval of the study protocol must also be provided in the manuscript.

Plants

Seed stocks	N/A
Novel plant genotypes	N/A
Authentication	N/A
