# Supplementary material for: Dual activation of NLRP3 and pyrin inflammasomes mediates host responses to the human fungal pathogen Coccidioides
Source: Nat Commun. 2026 Jul 1;17:8209. doi: 10.1038/s41467-026-75022-8 (PMC13462806; doi:10.1038/s41467-026-75022-8)
Supplement: Supplementary file 4 [file 41467_2026_75022_MOESM7_ESM.pdf]

nature portfolio

Peer Review File

Dual Activation of NLRP3 and Pyrin Inflammasomes Mediates Host Response to the Human Fungal Pathogen

Corresponding Author: Dr Sinem Beyhan

This file contains all reviewer reports in order by version, followed by all author rebuttals in order by version.

Version 0:

Reviewer comments:

Reviewer #1

(Remarks to the Author)
This paper discusses inflammasome activation and cell death pathways during infection by Coccidioides posadasii with a range of fungal morphologies (arthroconidia, spherules, endospores). The authors use knockout macrophages, in vivo murine models, and imaging approaches to dissect the functions of the NLRP3 inflammasome and pyrin inflammasomes in the host response to coccidiomycosis. The data sets are extensive, and the work addresses an important gap in fungal immunology with an endemic and important human fungal pathogen. However, the manuscript is quite dense and at times more descriptive than mechanistic, and so would be improved with better framing and a more integrated connection between the results sections. The paper reads like a random assortment of observations with multiple missed opportunities to drive mechanistically into the crux of the question. The novelty is high, but conclusions are sometimes overstated based on the data, and subsequent mechanistic insights are limited.

Major

1. The introduction is long and reads a lot like a review. The knowledge gap is stated only in generic terms (the role of inflammasome components in controlling Coccidioides infections has not been reported). This undersells the motivation for this research and reframing to specify what is known (IL-1β release in infected lungs) and what remains unknown (the specificity of this to fungal morphologies and inflammasome pathways involved). A sharper statement of the hypothesis or aims at the end of the Introduction would better set up the results.

2. Supplementary Figure 1, the z-VAD-FMK data demonstrate that macrophage death is caspase-independent. However, the effect of caspase inhibition on IL-1β release was not shown. This would be a useful measurement, as it provides important confirmation of inflammasome dependency, which would strengthen the mechanistic conclusions.

3. The authors use the first section of the results to discuss how arthroconidia induce macrophage death, then swing to “ferroptotic lipid peroxidation killing arthroconidia without altering macrophage viability”. This is literary whiplash.

4. Arthroconidia are shown to require priming for IL-1β release, but spherules induce IL-1β without priming, particularly when they rupture. The mechanistic reason for this difference is not explored. Are spherules providing both signals, or do they intrinsically trigger NF-κB signaling? A measurement of pro–IL-1β induction would help strengthen the mechanism.

5. In Figure 2, the conclusion that ferroptosis is the main pathway of arthroconidia-induced macrophage death is overstated. Liproxstatin-1 treatment actually enhanced cell death rather than preventing it. This suggests that ferroptotic lipid peroxidation contributes to fungal control, but not that ferroptosis is the dominant mode of cell death. So, the claims in the abstract and discussion sections should be tempered to reflect this nuance. Here is a good example of a lack of follow-up. There are no experiments to demonstrate the role of ferroptosis in this observation. It would have been nice to see data on glutathione peroxidase 4 (GPX4) and inhibition by ferrostatin-1 (FER-1), at a minimum.

6. In Figure 3, the authors discuss activation of dual inflammasome activation, which is interesting. However, this appears to mostly be inferred from single knockouts. Double knockouts, eg, Nlrp3-/-Mefv-/- Mefv-/-, are needed to make this claim; otherwise, redundancy might be possible.

7. In Figure 7, the interpretation of the survival and weight loss experiments is overstated. WT mice only reached the >20% weight loss endpoint by day 11, which generally indicates a relatively slow disease course. Casp1/11$^{-/-}$ mice showed only modestly delayed weight loss, with no difference in lung fungal burden. So the statement that caspase-1/11 ‘protects against disease severity’ is too strong. A more cautious interpretation is required.

8. For experiments involving spherule stimulation, what is the percentage contamination of arthroconidia and hyphae?

9. The authors use “FunGalFinder” as an analytic tool to detect Cocci spherules. However, the reference cited (53) describes Fiji, not this macro. Has this tool been published before, demonstrating its sensitivity and specificity? If this is the first publication of this tool, then there should be additional supplemental data that demonstrates the performance characteristics of this tool.

Minor

1. Scale bars need to be added to all microscopy panels.

2. At times, the term ‘fixed-spherules’ is used rather than formalin-killed spherules or FKA. How are these different? Abbreviations should be standardized throughout the manuscript, as it is confusing to the reader.

3. Was endotoxin testing conducted on formalin-killed arthroconidia and spherule stocks? Checks for endotoxin presence were not mentioned, but this would be useful information, given the inflammasome assay, as any trace LPS could impact IL-1beta results.

4. Some experiments are lacking positive controls. In the case of ferroptosis experiments, there is no positive control for inhibitor activity that would give a known ferroptosis stimulus (eg: RSL3 and erastin). Again, in the z-VAD-FMK experiments, there is no positive control for use with the caspase inhibitor that would demonstrate induction of apoptosis, such as TNFalpha.

5. MOIs are not justified consistently across experiments throughout the manuscript. A short explanation would enable easier reading.

6. IL-1β release is shown to require higher MOIs is repeated across conditions, but the physiological relevance is not discussed. Do these MOIs approximate what alveolar macrophages encounter in vivo, or are they supraphysiological?

Reviewer #2

(Remarks to the Author)
I co-reviewed this manuscript with one of the reviewers who provided the listed reports. This is part of the Nature Communications initiative to facilitate training in peer review and to provide appropriate recognition for Early Career Researchers who co-review manuscripts.

Reviewer #3

(Remarks to the Author)
The authors devote considerable experimental effort to comprehensively analyze how different forms of the dimorphic fungus Coccidioides – arthroconidia (live and dead), spherules (intact and ruptured) - influence cell death and cytokine release of macrophages. The work is largely done in vitro with bone marrow derived macrophages (BMDM). These BMDM are from C57B/l6 mice and a large number of related transgenic strains (n = about 14 strains) with gene defects in inflammasome activation pathways, cell death pathways, TLRs, CLRs and inflammatory cytokines (IL-1B and IL-18). Additional work is done with sophisticated imaging to address how some of these genes influence the behavior of these fungal forms in the BMDM in vitro. At the end of these extensive in vitro studies, an in vivo experimental infection is performed to look at survival and lung fungal burden with two strains of knockout mice (Caspase 1 and Caspase 11) in an effort to establish a biological link with this disease. Regrettably, the depth of in vivo studies is limited and superficial with no cellular or molecular analysis. Thus, I do not find the in vivo mouse studies to be a compelling translation of in vitro findings or link to the biology of this disease. On balance then, the study offers a comprehensive survey of a large cohort of knockout mice with too little effort at the end of the story to validate in vitro findings at a cellular and molecular level. As one example, the investigators could interrogate alveolar macrophages in wild type and representative knockout mouse strains for important pathways, perhaps harboring different forms/stages of the fungus to validate the key in vitro observations they made using BMDMs.

Reviewer #4

(Remarks to the Author)
In the manuscript “Dual Activation of NLRP3 and Pyrin Inflammasomes Mediates Host Response to a Human Fungal Pathogen,” Yau et al. report the role of inflammasomes in Coccidioides infection. The authors investigated the interactions of Coccidioides at different developmental stages, including arthroconidia, spherules, and endospores, with ferroptosis and inflammasome pathways that control Coccidioides viability, IL-1β production, and macrophage death. They demonstrate the involvement of NLRP3 and pyrin inflammasomes, and inflammatory cell death signaling involving gasdermin D, and MLKL during the host response to Coccidioides. This manuscript has significant weaknesses, such as insufficient data and a lack

of mechanistic insights. Specific comments are included below.

1. A significant weakness of the paper is that the conclusions are not fully supported by data, and in some instances, the data contradict the conclusions. It is concluded that the NLRP3 inflammasome protects from the dissemination of Coccidioides. But Coccidioides infection was examined in NLRP3 KO mice. Caspase-1/11 KO mice data are insufficient to conclude a role for NLRP3.

2. Weight loss and mortality were reduced in caspase-1/11 KO, which contradicts the conclusion that the caspase-1/11 signaling may be important for limiting Coccidioides disease severity.

3. Another weakness is the lack of mechanistic insights into the phenotypes observed. For instance, how do arthroconidia activate pyroptotic, necroptotic, and ferroptotic pathways?

4. The rationale for specifically testing the role of pyrin (among other inflammasomes) in arthroconidia-induced macrophage death and IL-1β release is not clear, and the pyrin inflammasome part seems superficial.

5. Did live arthroconidia (LA) induced macrophage death and IL-1β release require LPS priming? If not, how do live arthroconidia prime macrophages? via dectin-1?

6. Do arthroconidia induce the activation of GSDME/GSDMD/MLKL in macrophages?

7. Whether arthroconidia induced ferroptosis in infected cells remains unclear, as Lip-1 did not block macrophage death but increased it following LA infection. Therefore, more data demonstrating lipid peroxidation and ferroptosis is needed to support the conclusion.

8. Confirmation of cell death with the LDH assay is recommended.

9. Interestingly, killed arthroconidia-induced IL-1b release was Nlrp3 dependent, but live arthroconidia-induced cell death was Nlrp3 independent. This discrepancy should be addressed.

10. How do killed arthroconidia inhibit cell death response to live arthroconidia and promote spherule formation in macrophages?

11. Line 299-700: Figures were cited incorrectly.

Version 1:

Reviewer comments:

Reviewer #1

(Remarks to the Author)
The authors have addressed my comments adequately. I would ask that all data that was shown in the response to reviews be included in the supplementary data and callouts included in the text. these are important data that should be shown to the research community, but does not rise to the importance to be added to the body of the MS.

*Editorial note
Reviewer 3 was unable to provide a response at this stage and we asked reviewer 4 to additionally comment in their place on the authros response to reviewer 3:

I believe the authors have addressed the concerns of Reviewer 3.

(Remarks on code availability)

Reviewer #4

(Remarks to the Author)
The authors have addressed my comments through textual and experimental revisions.

*Editorial note
Reviewer 3 was unable to provide a response at this stage and we asked reviewer 4 to additionally comment in their place on the authros response to reviewer 3:

The authors have adequately addressed Reviewer 3’s comments through in vivo experiments.

(Remarks on code availability)

Open Access This Peer Review File is licensed under a Creative Commons Attribution 4.0 International License, which permits use, sharing, adaptation, distribution and reproduction in any medium or format, as long as you give appropriate credit to the original author(s) and the source, provide a link to the Creative Commons license, and indicate if changes were made.
In cases where reviewers are anonymous, credit should be given to 'Anonymous Referee' and the source.
The images or other third party material in this Peer Review File are included in the article's Creative Commons license, unless indicated otherwise in a credit line to the material. If material is not included in the article's Creative Commons license and your intended use is not permitted by statutory regulation or exceeds the permitted use, you will need to obtain permission directly from the copyright holder.
To view a copy of this license, visit https://creativecommons.org/licenses/by/4.0/

REVIEWER COMMENTS

Reviewer #1 (Remarks to the Author):

This paper discusses inflammasome activation and cell death pathways during infection by Coccidioides posadasii with a range of fungal morphologies (arthroconidia, spherules, endospores). The authors use knockout macrophages, in vivo murine models, and imaging approaches to dissect the functions of the NLRP3 inflammasome and pyrin inflammasomes in the host response to coccidioidomycosis. The data sets are extensive, and the work addresses an important gap in fungal immunology with an endemic and important human fungal pathogen. However, the manuscript is quite dense and at times more descriptive than mechanistic, and so would be improved with better framing and a more integrated connection between the results sections. The paper reads like a random assortment of observations with multiple missed opportunities to drive mechanistically into the crux of the question. The novelty is high, but conclusions are sometimes overstated based on the data, and subsequent mechanistic insights are limited.

Major

1. The introduction is long and reads a lot like a review. The knowledge gap is stated only in generic terms (the role of inflammasome components in controlling Coccidioides infections has not been reported). This undersells the motivation for this research and reframing to specify what is known (IL-1β release in infected lungs) and what remains unknown (the specificity of this to fungal morphologies and inflammasome pathways involved). A sharper statement of the hypothesis or aims at the end of the Introduction would better set up the results.

We thank the reviewer for this perspective and have improved the messaging to better convey the motivation for the study. We have added the following text to the end of the introduction: *"Due to its dimorphic lifestyle, Coccidioides can interact with host cells in three different forms: arthroconidia, spherules, and endospores. There is a knowledge gap regarding how these different forms of Coccidioides interact with host immune cells. In this study, we systematically examined how arthroconidia, spherules, and endospores differentially engage innate immune sensing pathways and downstream effector mechanisms in macrophages and neutrophils. Using genetic and pharmacologic approaches, we dissected the relative contributions of Dectin-1 and TLR2 signaling, as well as inflammasomes, and regulated cell death programs, including pyroptosis, ferroptosis-associated lipid peroxidation, and necroptotic signaling. Our findings reveal a morphology-specific, and multifaceted host immune response to Coccidioides, characterized by coordinated yet distinct fungal sensing, inflammasome activation, and regulated cell death signaling in macrophages and neutrophils."*

2. Supplementary Figure 1, the z-VAD-FMK data demonstrate that macrophage death is caspase-independent. However, the effect of caspase inhibition on IL-1β release was not shown. This would be a useful measurement, as it provides important confirmation of inflammasome dependency, which would strengthen the mechanistic conclusions.

We thank the reviewer for this comment and have performed additional experiments to explore this question. To investigate the effects of caspase inhibition in BMDM, we first obtained new stocks of Z-VAD(OMe)-FMK and performed a dose titration together with a combination of Bcl-2 family antagonists as a positive control (***Response_to_Reviewer_Figure 1, Response_to_Reviewer_Figure 2:*** see below). Additionally, we compared the response of BMDM to Q-VD-OPh. These data revealed more complex effects of caspase inhibition in uninfected BMDM, including notable increases in cell death, likely attributable to effective inhibition of Caspase-8 and subsequent activation of MLKL. This was confirmed using BMDM deficient in MLKL. BMDM deficient in Caspase-1/11 were also partially resistant to Z-VAD(OMe)-FMK.

When examining the viability of the positive control samples treated with Bcl-2 family antagonists (S63845+ABT-199+A-1331852), similar effects could be seen except that BMDM treated with Q-VD-OPh also engaged MLKL to kill BMDM, but to a lesser magnitude compared to BMDM treated with Z-VAD(OMe)-FMK. In these samples undergoing mitochondrial apoptosis, the loss of MLKL abrogated cell death in the presence of caspase inhibitors but not in PBS or DMSO-treated controls. When considering IL-1β production, treatment of BMDM with Z-VAD(OMe)-FMK induced low levels of IL-1β that were dependent on Caspase-1/11, and MLKL.

These observations are not without precedent and have been noted by other investigators in the presence of TNF or IAP antagonists, but typically with priming by LPS (PMID:27194727; 25693118; 28723569; 30485804). Here the GM-CSF priming likely alters basal levels of TNF production or IAP stability to enable activation of MLKL. The caspase inhibitors Z-VAD(OMe)-FMK, Q-VD-OPh, and IDN-6556 also show differential potency in sensitizing AML cells to birinapant-induced cell death, via differences in ability to inhibit Caspase-8/cFLIP$_L$ heterodimers (catalytically active that blocks necroptosis) and Caspase-8 homodimers. IDN-6556 and Z-VAD(OMe)-FMK blocked the Caspase-8/cFLIP$_L$ heterodimers more efficiently in comparison to Q-VD-OPh. These studies also demonstrate that IDN-6556 and Z-VAD(OMe)-FMK were unable to restrain apoptosis via homodimeric Caspase-8 in necroptosis-deficient cells.

These are interesting observations in GM-CSF-primed macrophages that will require further biochemical study but are well beyond the scope and focus of this manuscript. We have removed supplementary Figure 1 referencing Z-VAD-FMK, instead focusing on the use of mouse genetic tools to mechanistically explore the role of inflammasomes and necroptotic machinery in response to *Coccidioides*.

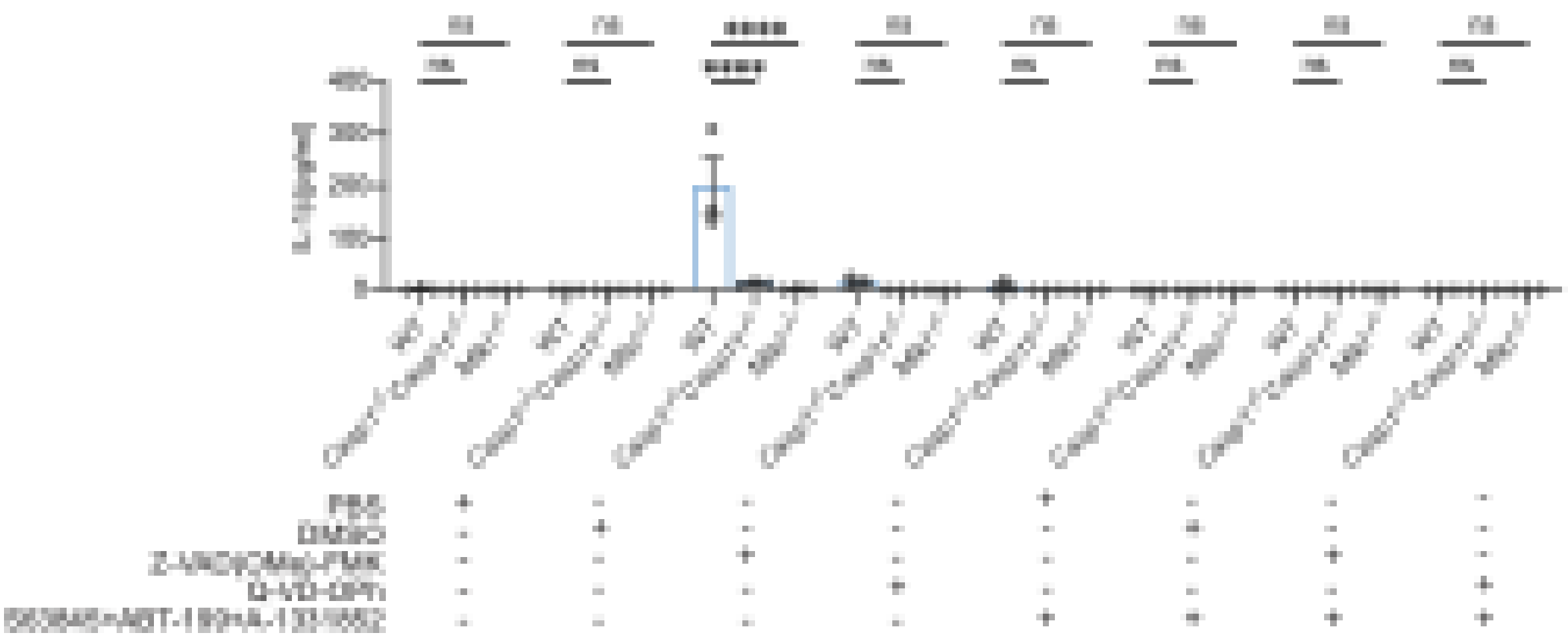

Response_to_Reviewer_Figure 1: IL-1β release from WT, *Casp1*$^{-/-}$*Casp11*$^{-/-}$, and *Mlkl*$^{-/-}$ BMDMs treated with 50 µM Z-VAD(Ome)-FMK, 50 µM Q-VD-OPh, 10 µM S63845, 10 µM ABT-199, 10 µM A-1331852, or DMSO. Mean ± SEM, n = 3, ****P < 0.0001, ns: not significant, WT vs knockouts, two-way ANOVA.

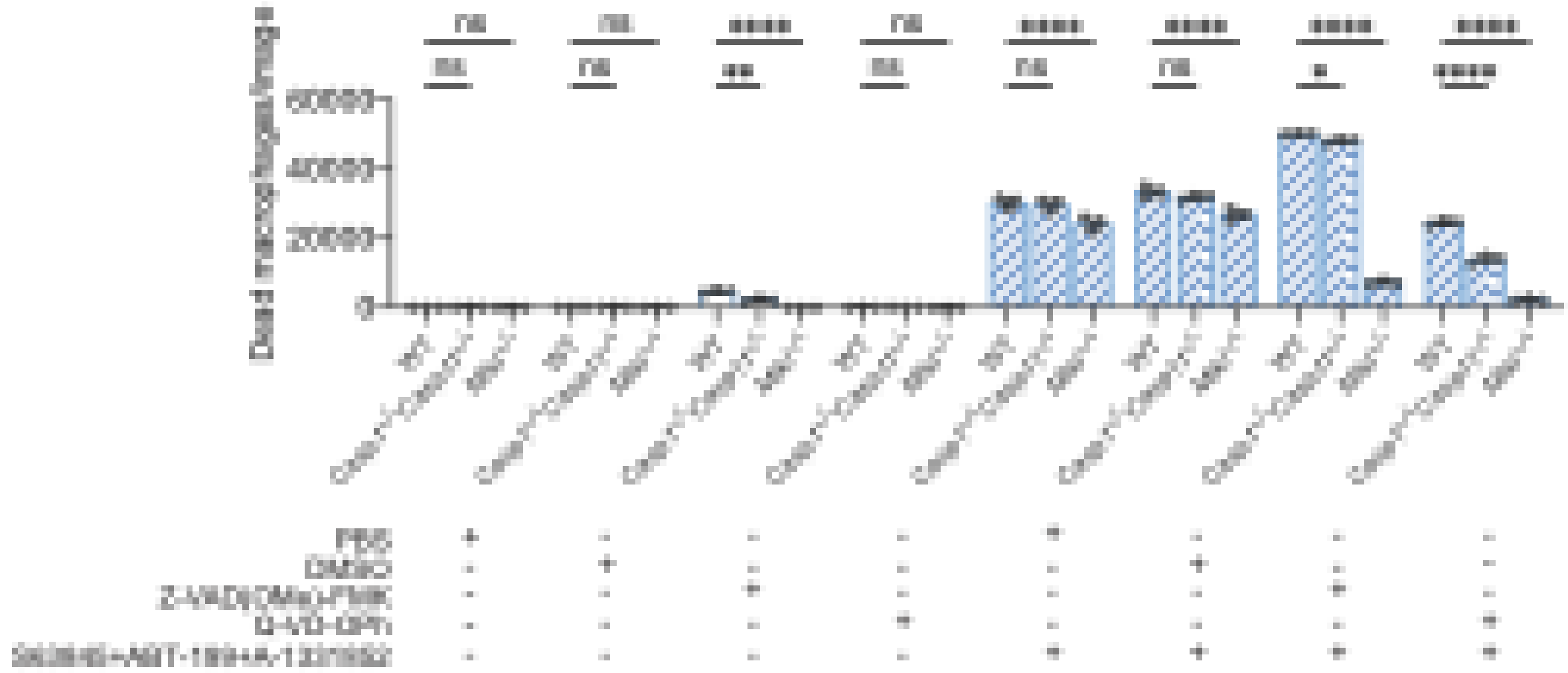

Response_to_Reviewer_Figure 2: Cell viability by live-cell imaging and automated image analysis (whole well scans) of WT, *Casp1*$^{-/-}$*Casp11*$^{-/-}$, and *Mlkl*$^{-/-}$ BMDMs treated with 50 µM Z-VAD(Ome)-FMK, 50 µM Q-VD-OPh, 10 µM S63845, 10 µM ABT-199, 10 µM A-1331852, or DMSO. Mean ± SEM, n = 3, *P < 0.05, **P < 0.005, ****P < 0.0001, ns: not significant, WT vs knockouts, two-way ANOVA.

3. The authors use the first section of the results to discuss how arthroconidia induce macrophage death, then swing to "ferroptotic lipid peroxidation killing arthroconidia without altering macrophage viability". This is literary whiplash.

We thank the reviewer for this comment. We have updated the text and Figures 1 and 2 to improve clarity and simplify messaging.

4. Arthroconidia are shown to require priming for IL-1β release, but spherules induce IL-1B without priming, particularly when they rupture. The mechanistic reason for this difference is not explored. Are spherules providing both signals, or do they intrinsically trigger NF-kB

signaling? A measurement of pro–IL-1B induction would help strengthen the mechanism.

The reviewer notes an interesting point. Our data demonstrates that fixed spherules (FKS) and fixed arthroconidia (FKA) do not induce IL-1β secretion without a priming step (Signal 1). In contrast, live arthroconidia can induce IL-1β secretion in a MOI-dependent manner (Figure 1). To support this claim, we have added ***new data (Figure 1G)*** showcasing IL-1β secretion by BMDMs is dependent on TLR2, NLPR3, and GSDME activation.

Figure 1

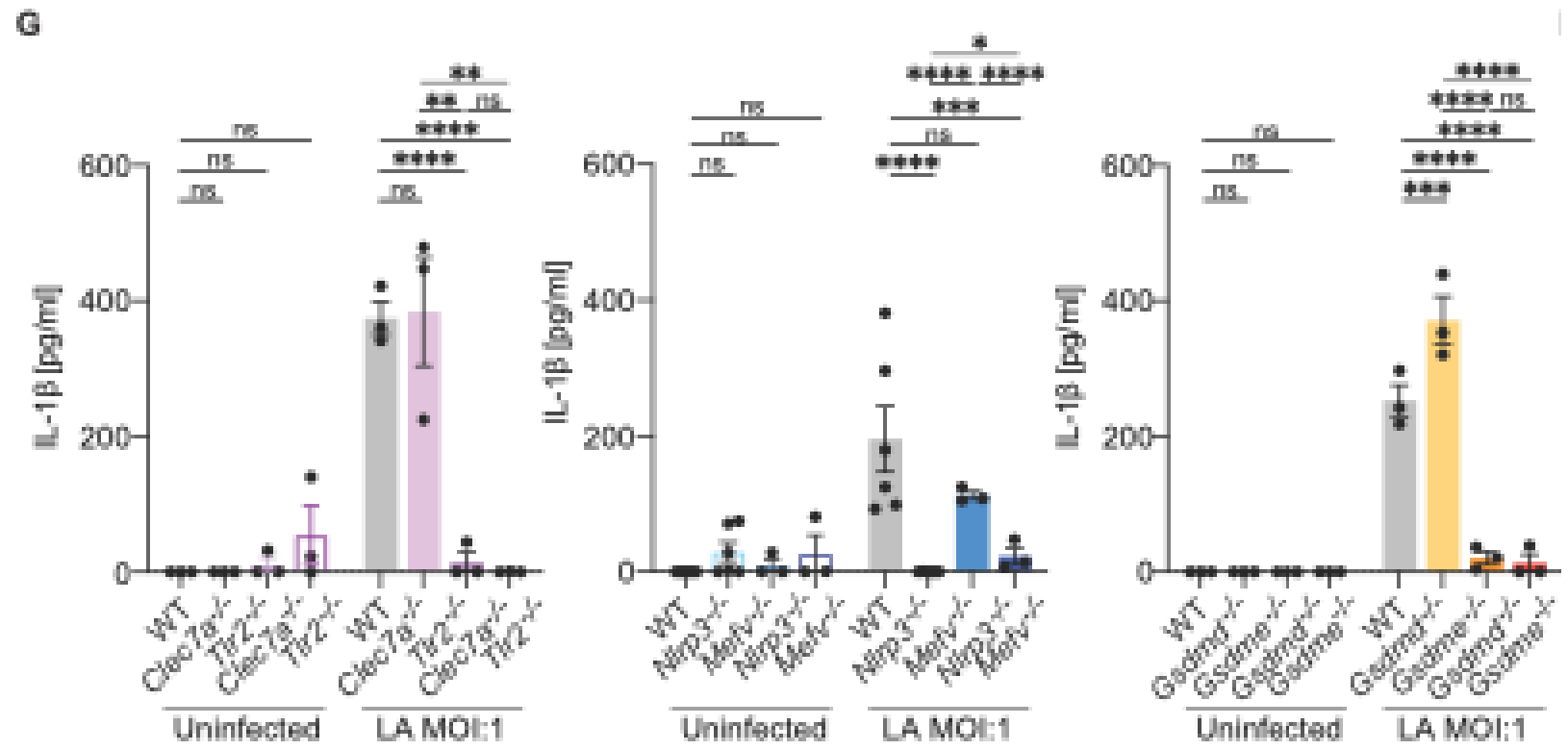

Additionally, live spherules can induce IL-1β secretion but only after spherule rupture (Figures 3 & 5). The generation of spherules from live arthroconidia (usually an MOI 0.01) is a 3-4 day assay and it is likely that the BMDM are exposed to inflammatory or fungal ligands (signal 1), such as SOW-gp and β-glucan, during this period causing pro-IL-1β induction. It is only upon rupture of those intact spherules exposing these ligands, that pyrin/NLRP3 are engaged to enable the processing of pro-IL-1β and release of IL-1β. We added ***new data (Figure 4F–H)*** demonstrating that β-glucan supplementation under spherule-inducing conditions further enhanced spherule formation and growth while reducing BMDM death.

Figure 4

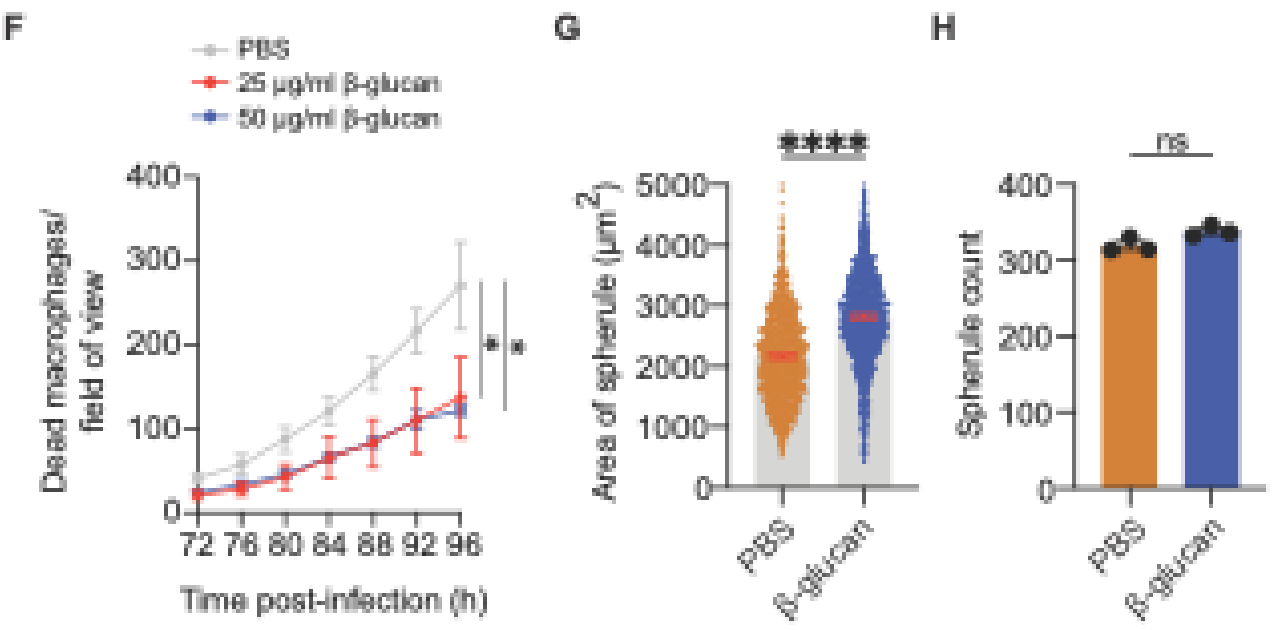

As illustrated in Figure 6, endospores are also released and can activate NLRP3. We have yet to study priming (signal 1) of BMDM at different stages of the arthroconidia-spherule transition over this multi-day assay. Endospores alone do not elicit the same magnitude of IL-1β release, suggesting that the multi-day priming of BMDM, firstly by arthroconidia and secondly by developing spherules, as well as SOW-gp and β-glucan, underlie the higher levels of IL-1β released by rupturing spherules (PMID:12065484). We show that IL-1b production in response to FKA and FKS requires Pam_2CSK_4 priming (Figure 1, Figure 3). We performed additional experiments to show that priming is required for induction of pro-IL-1b allowing

processing to bioactive IL-1b upon exposure to FKA (***Response_to_Reviewer_Figure 3***: see below).

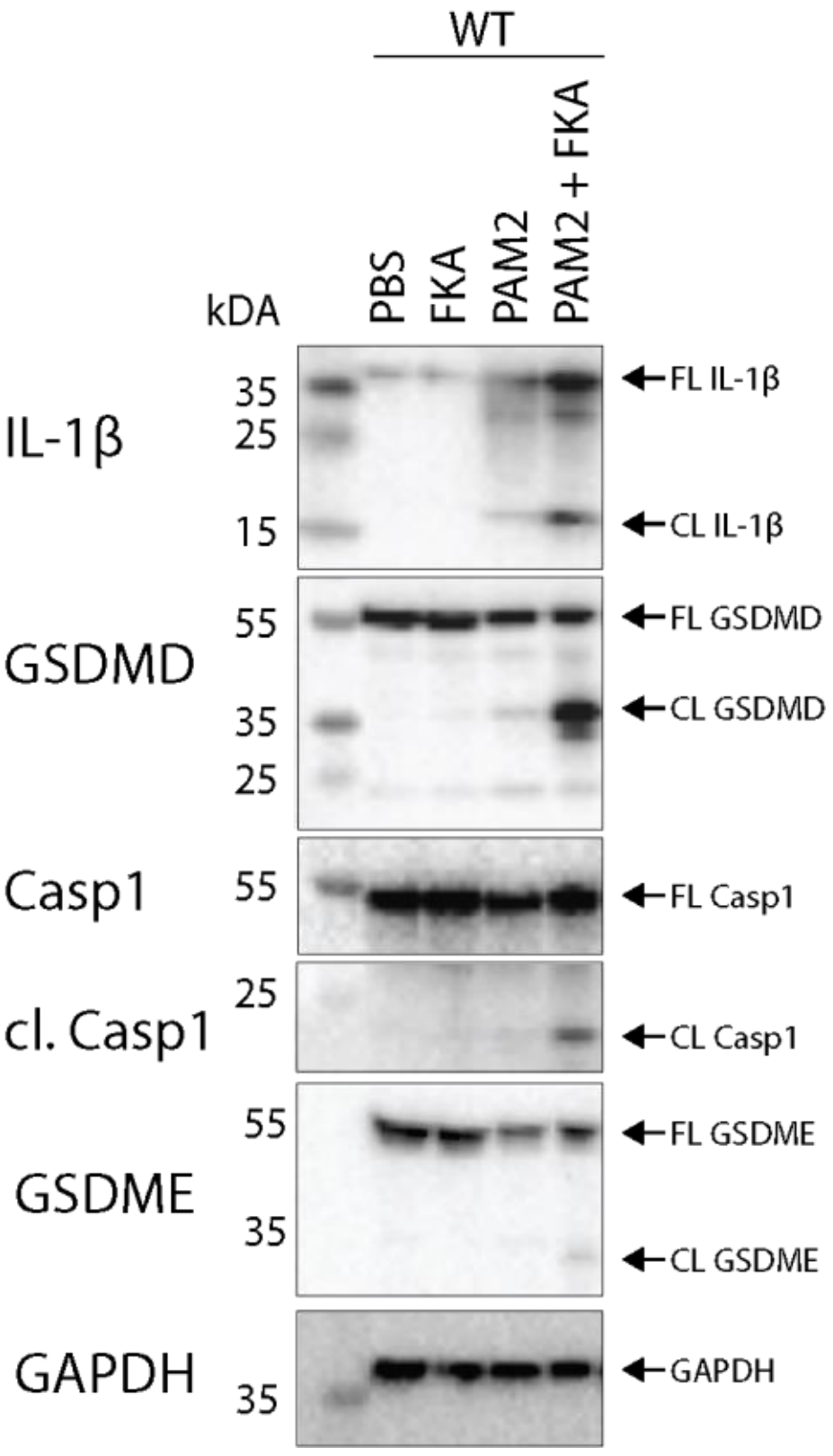

Response_to_Reviewer_Figure 3: Immunoblot analysis of WT BMDMs treated with FKA at MOI:1, with or without PAM_2CSK_4 priming. Full-length and cleaved IL-1β, GSDMD, Caspase-1, GSDME, and GAPDH of cell lysates were analyzed by immunoblot.

5. In Figure 2, the conclusion that ferroptosis is the main pathway of arthroconidia-induced macrophage death is overstated. Liproxstatin-1 treatment actually enhanced cell death rather than preventing it. This suggests that ferroptotic lipid peroxidation contributes to fungal control, but not that ferroptosis is the dominant mode of cell death. So, the claims in the abstract and discussion sections should be tempered to reflect this nuance. Here is a good example of a lack of follow-up. There are no experiments to demonstrate the role of ferroptosis in this observation. It would have been nice to see data on glutathione peroxidase 4 (GPX4) and inhibition by ferrostatin-1 (FER-1), at a minimum.

We thank the reviewer for this comment. This finding was also a surprise to us and was reflected in the subsection title: "*Ferroptotic lipid peroxidation kills arthroconidia without altering macrophage viability*". We also noted that "*Lip-1 did not block macrophage death but increased it*". We went on to say that: "*These data suggest that iron-dependent lipid peroxidation may damage arthroconidia as a consequence of ferroptosis in BMDM*". Here we used 'ferroptosis' as a reference to a signaling pathway rather than a form of cell death – instead we have now edited that text to refer to this effect as ferroptotic lipid peroxidation. Wording in the abstract and the discussion have also now been edited to reflect the role of ferroptotic lipid peroxidation, rather than ferroptosis, in fungal control.

Additionally, to support the role of ferroptotic lipid peroxidation, we show that Fer-1 inhibition of ferroptotic signalling increases macrophage death similar to the other ferroptosis inhibitor Lip-1 (***Response_to_reviewer_Figure 4***).

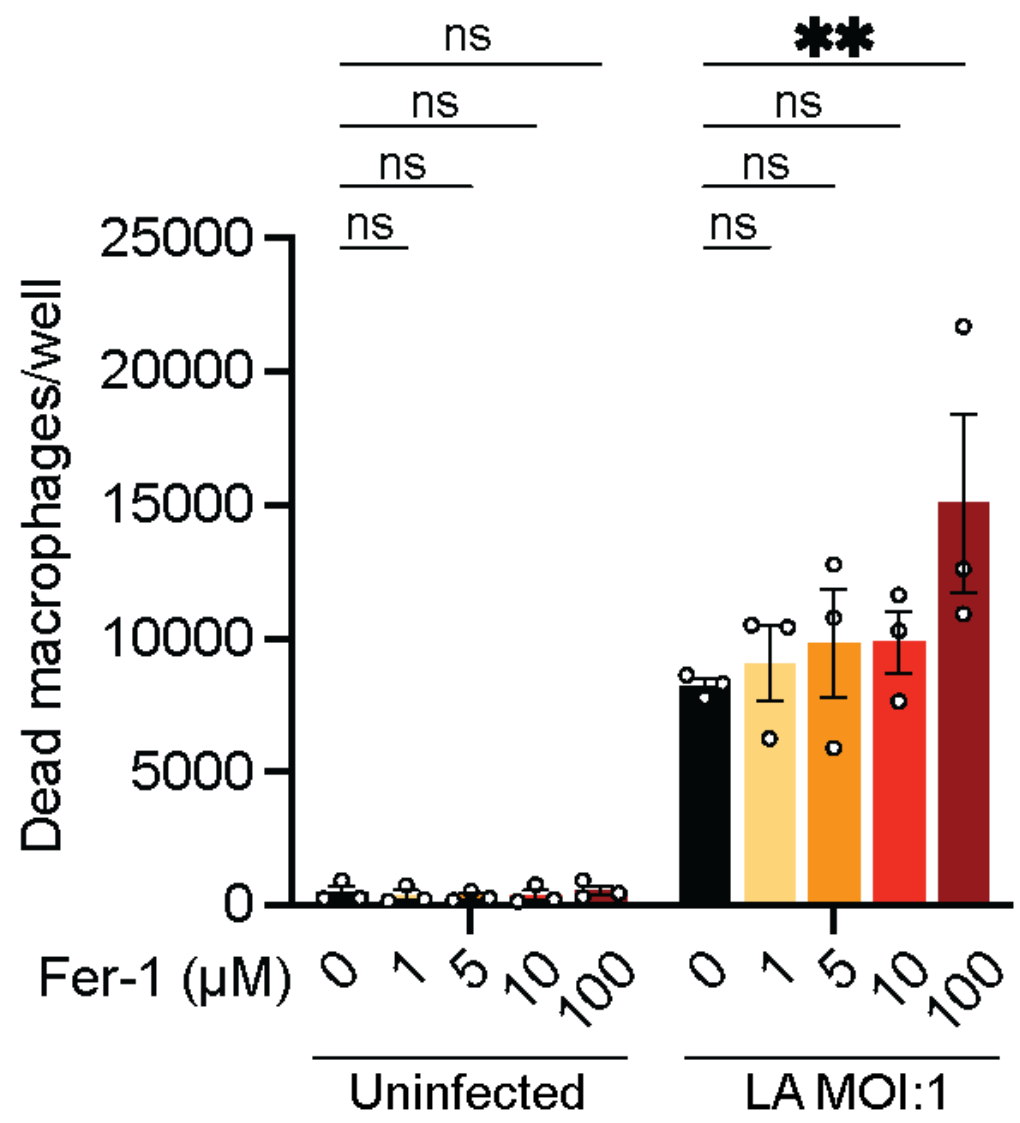

Response_to_Reviewer_Figure 4: Cell viability by live-cell imaging and automated image analysis (whole well scans) of WT BMDMs infected with LA at MOI:1, and treated with ferrostatin-1 (Fer-1) at 1, 5, 10, and 100 µM. Mean ± SEM, n = 3, $**P < 0.005$, ns: not significant, WT vs knockouts, two-way ANOVA.

6. In Figure 3, the authors discuss activation of dual inflammasome activation, which is interesting. However, this appears to mostly be inferred from single knockouts. Double knockouts, eg, Nlrp3-/-Mefv-/- Mefv-/-, are needed to make this claim; otherwise, redundancy might be possible.

We thank the reviewer for raising this point. The *Nlrp3*$^{-/-}$*Mefv*$^{-/-}$ double knockout mice were presented in Figures 1, 5, and 7. For example, in Figure 7L, the individual loss of *Nlrp3*$^{-/-}$ and *Mefv*$^{-/-}$ in neutrophils exposed to *C. posadasii* Silveira FKS partially inhibited the release of IL-1β, whereas loss of both NLRP3 and pyrin in neutrophils from *Nlrp3*$^{-/-}$*Mefv*$^{-/-}$ mice completely inhibited IL-1β release. These data suggest that both NLRP3 and pyrin inflammasomes are activated in neutrophils upon exposure to spherules and are important for maximal inflammasome activation and IL-1β secretion.

7. In Figure 7, the interpretation of the survival and weight loss experiments is overstated. WT mice only reached the >20% weight loss endpoint by day 11, which generally indicates a relatively slow disease course. Casp1/11^{-}*/*$^{-}$ *mice showed only modestly delayed weight loss, with no difference in lung fungal burden. So the statement that caspase-1/11 'protects against disease severity' is too strong. A more cautious interpretation is required.*

We thank the reviewer for this comment and apologise for the misstatement when referring the KO mice. Wording in the results have been edited to correctly reflect the role of Caspase-1 protein in weight loss and disease-free survival of *Coccidioides*-infected mice. We have

corrected the subtitle to this: "*Activation of Caspase-1/11 exacerbates coccidioidomycosis in mice*".

8. For experiments involving spherule stimulation, what is the percentage contamination of arthroconidia and hyphae?

Contamination by arthroconidia and hyphae is difficult to accurately quantify using the described methods and Incucyte SX5 microscope due to the size and irregular morphology of arthroconidia and hyphae, but it appears negligible, and also greatly reduced under spherulation conditions (Figure 4C and ***Supplementary Movie 2***).

9. The authors use "FunGalFinder" as an analytic tool to detect Cocci spherules. However, the reference cited (53) describes Fiji, not this macro. Has this tool been published before, demonstrating its sensitivity and specificity? If this is the first publication of this tool, then there should be additional supplemental data that demonstrates the performance characteristics of this tool.

We thank the reviewer for this comment and apologise for the confusion. FunGal Finder (FGF) is a novel, custom-scripted code using Fiji and CellPose as a macro for accurate spherule segmentation. The related reference has been corrected in the text. The ***new Supplementary Figure 1*** now illustrates the generation of spherule masks by FGF.

Supplementary Fig. 1

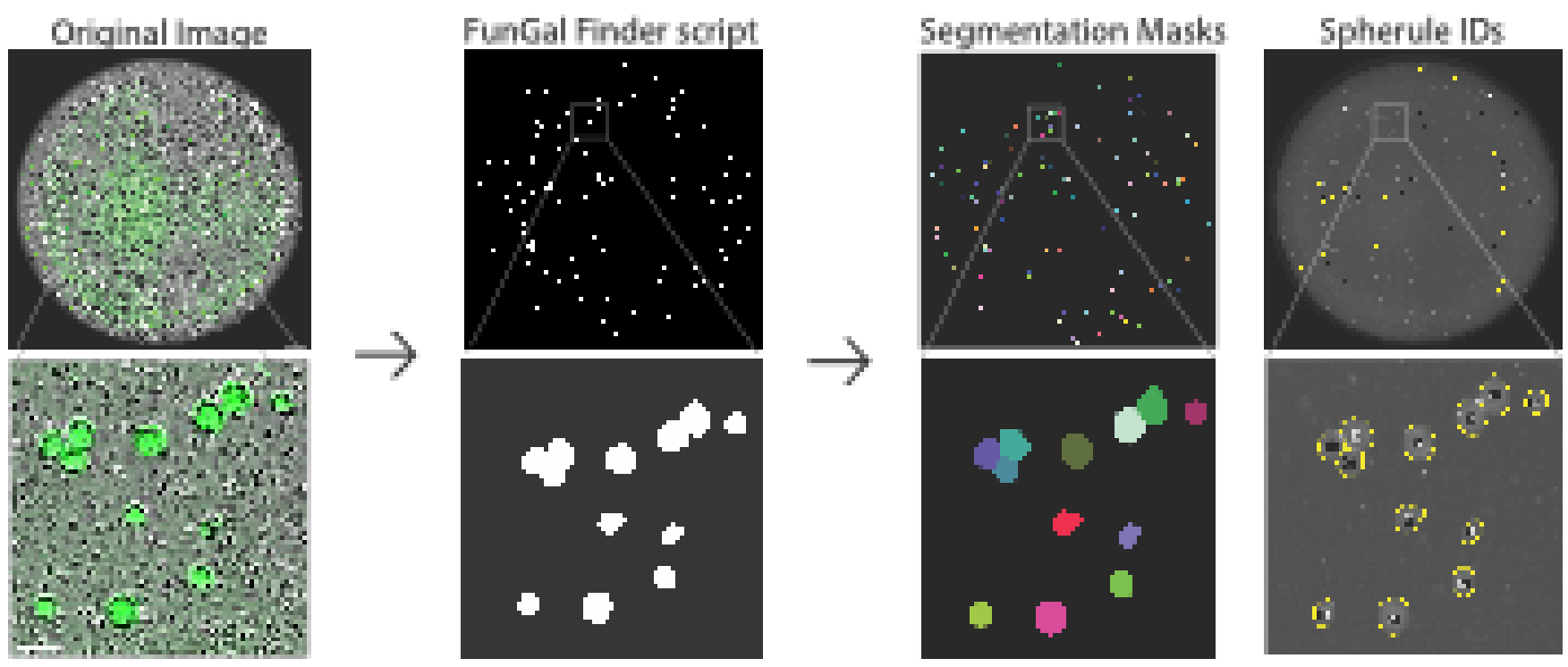

Minor

1. Scale bars need to be added to all microscopy panels.

We thank the reviewer for this comment. The figures have been edited to include scale bars in all microscopy panels.

2. At times, the term 'fixed-spherules' is used rather than formalin-killed spherules or FKA. How are these different? Abbreviations should be standardized throughout the manuscript, as it is confusing to the reader.

We thank the reviewer for this comment and apologise for the confusion. 'Fixed spherules' are 'formalin-killed spherules' and were used interchangeably. 'FKA' are *Coccidioides* arthroconidia that are formalin-killed. The text has been edited to reduce ambiguity.

3. Was endotoxin testing conducted on formalin-killed arthroconidia and spherule stocks? Checks for endotoxin presence were not mentioned, but this would be useful information, given the inflammasome assay, as any trace LPS could impact IL-1βeta results.

We thank the reviewer for this suggestion. As a fungus, *Coccidioides* does not express LPS/endotoxin. However, we have now performed endotoxin testing on the FKA and FKS stocks showing that LPS was not present.

4. Some experiments are lacking positive controls. In the case of ferroptosis experiments, there is no positive control for inhibitor activity that would give a known ferroptosis stimulus (eg: RSL3 and erastin). Again, in the z-VAD-FMK experiments, there is no positive control for use with the caspase inhibitor that would demonstrate induction of apoptosis, such as TNFalpha.

Monocyte-macrophages are relatively resistant to ferroptotic cell death (PMID:38589531) but appear capable of ferroptotic lipid peroxidation. We have now performed additional experiments that show similar findings for 2 different ferroptosis inhibitors: Fer-1 and Lip-1 (***Response_to_Reviewer_Figure 4***). These data support the role of ferroptotic lipid peroxidation in damaging arthroconidia. This is an exciting but complex field with many opportunities for further research. However, this is well beyond the scope of this manuscript.

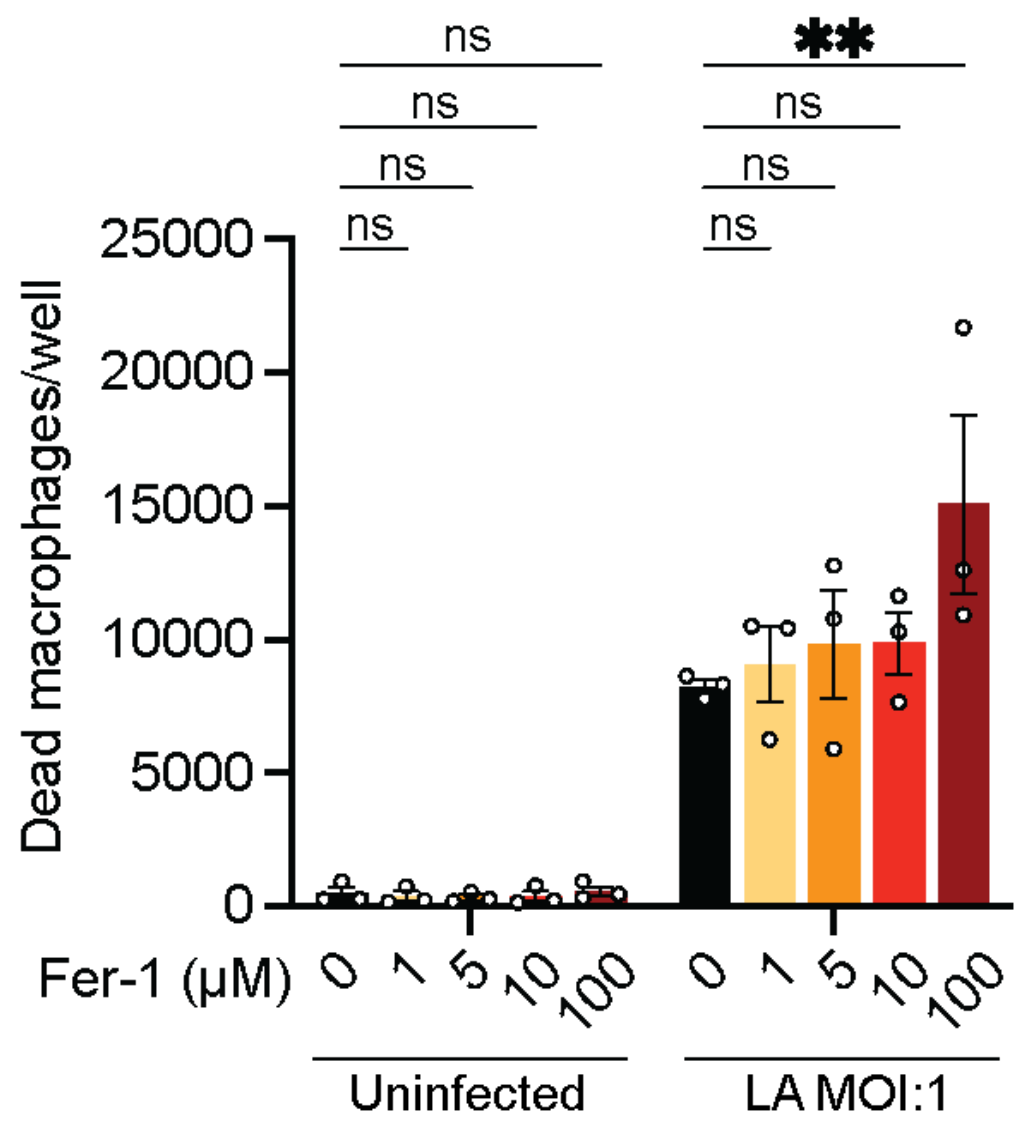

Response_to_Reviewer_Figure 5: Cell viability by live-cell imaging and automated image analysis (whole well scans) of WT BMDMs infected with LA at MOI:1, and treated with ferrostatin-1 (Fer-1) at 1, 5, 10, and 100 µM. Mean ± SEM, n = 3, $**P < 0.005$, ns: not significant, WT vs knockouts, two-way ANOVA.

To investigate the effects of caspase inhibition in apoptotic BMDM as a positive control, we first obtained new stocks of Z-VAD(OMe)-FMK and performed a dose titration together with a combination of Bcl-2 family antagonists (S63845+ABT-199+A-1331852) as a positive control (***Response_to_Reviewer_Figure 1***, ***Response_to_Reviewer_Figure 2***). Additionally, we compared the response of BMDM to Q-VD-OPh. These data revealed more complex effects of caspase inhibition in uninfected BMDM, including notable increases in cell death, likely attributable to effective inhibition of Caspase-8 and subsequent activation of RIPK3-MLKL. This

was confirmed using BMDM deficient in MLKL. BMDM deficient in Caspase-1/11 were also partially resistant to Z-VAD(OMe)-FMK-induced cell death.

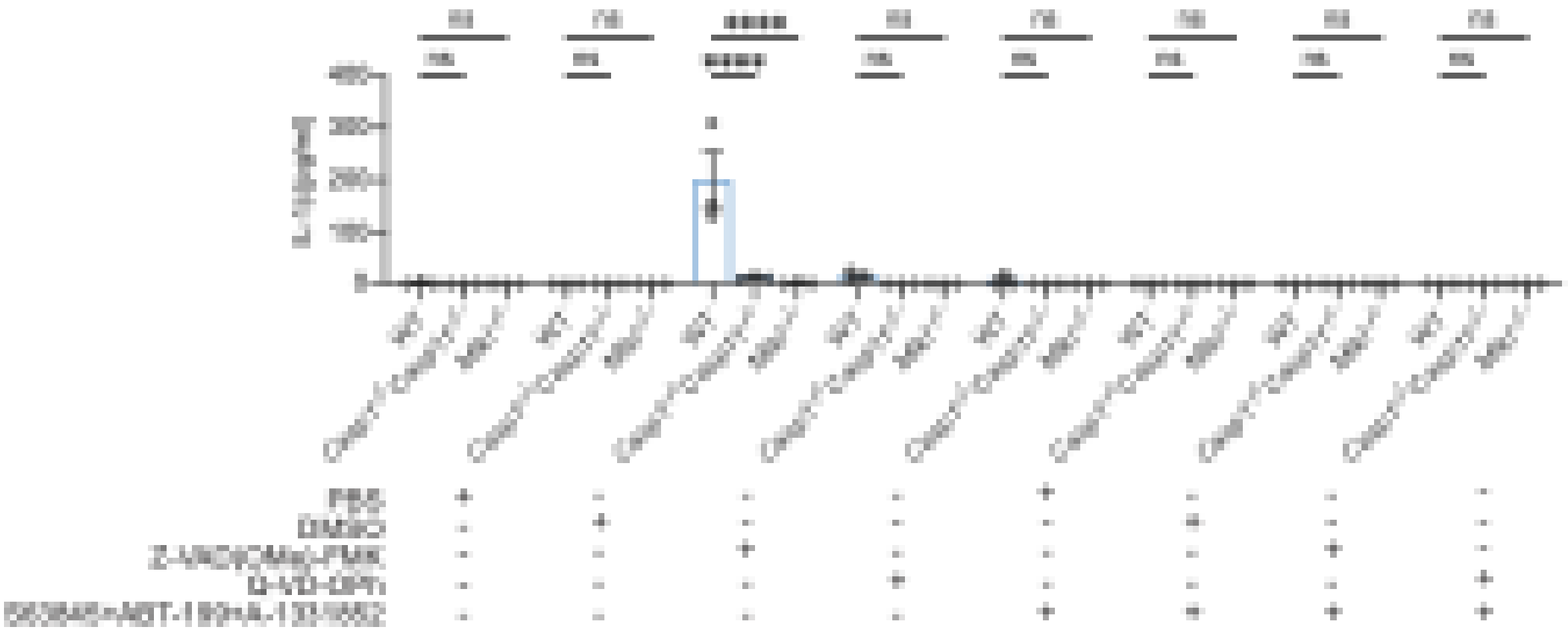

Response_to_Reviewer_Figure 6: IL-1β release from WT, *Casp1*$^{-/-}$*Casp11*$^{-/-}$, and *Mlkl*$^{-/-}$ BMDMs treated with 50 µM Z-VAD(Ome)-FMK, 50 µM Q-VD-OPh, 10 µM S63845, 10 µM ABT-199, 10 µM A-1331852, or DMSO. Mean ± SEM, n = 3, ****P < 0.0001, ns: not significant, WT vs knockouts, two-way ANOVA.

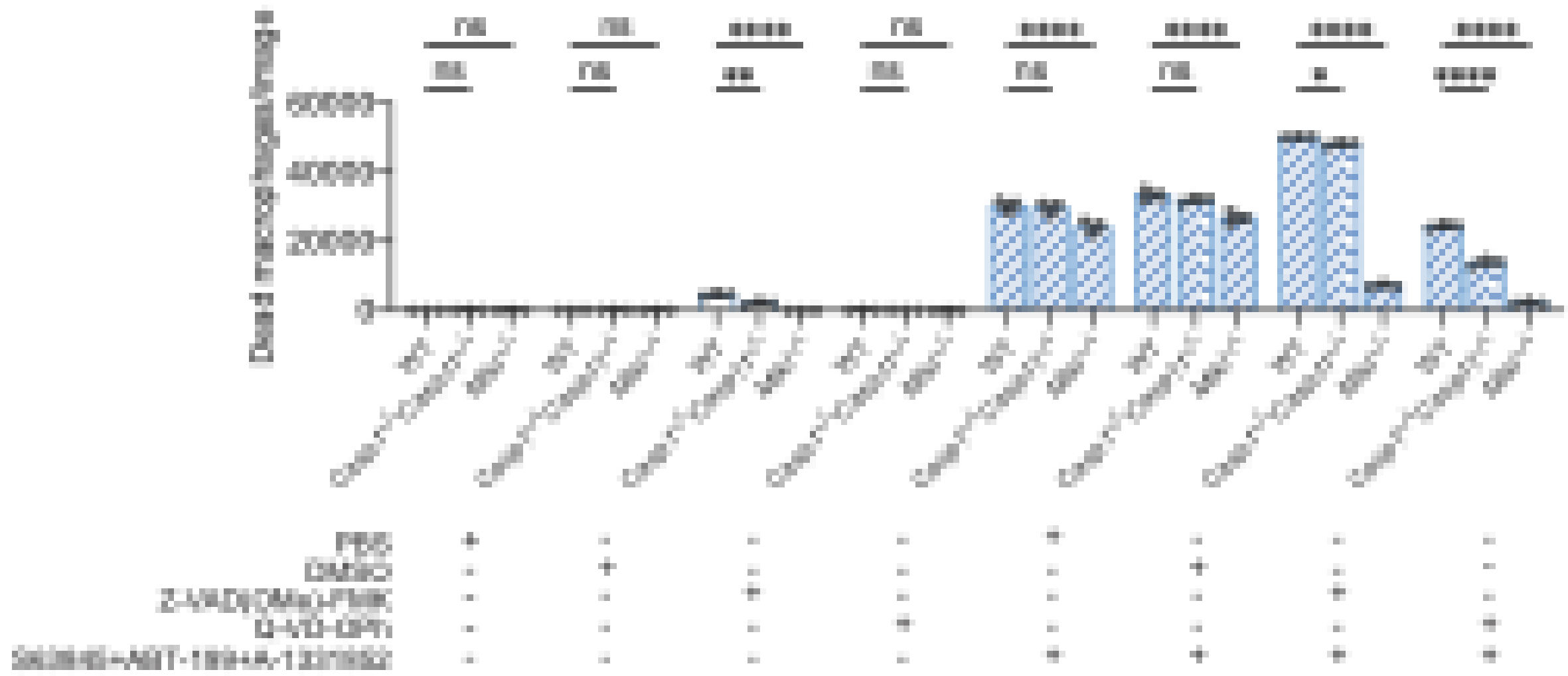

Response_to_Reviewer_Figure 7: Cell viability by live-cell imaging and automated image analysis (whole well scans) of WT, *Casp1*$^{-/-}$*Casp11*$^{-/-}$, and *Mlkl*$^{-/-}$ BMDMs treated with 50 µM Z-VAD(Ome)-FMK, 50 µM Q-VD-OPh, 10 µM S63845, 10 µM ABT-199, 10 µM A-1331852, or DMSO. Mean ± SEM, n = 3, *P < 0.05, **P < 0.005, ****P < 0.0001, ns: not significant, WT vs knockouts, two-way ANOVA.

When examining the viability of the positive control samples treated with Bcl-2 family antagonists (S63845+ABT-199+A-1331852), similar effects could be seen except that BMDM treated with Q-VD-OPh also engaged MLKL to kill BMDM, but to a lesser magnitude compared to BMDM treated with Z-VAD(OMe)-FMK. In these samples synchronized to undergo mitochondrial apoptosis by treatment with Bcl-2 family antagonists, the loss of MLKL abrogated cell death in the presence of caspase inhibitors but not in PBS or DMSO-treated controls.

When considering IL-1β production, treatment of BMDM with Z-VAD(OMe)-FMK induced low levels of IL-1β that were dependent on Caspase-1/11, and MLKL.

These are interesting observations that will require further biochemical study but are well beyond the scope and focus of this manuscript. We have removed supplementary Figure 1 referencing Z-VAD-FMK, instead focusing on the use of mouse genetic tools to mechanistically explore the role of inflammasomes and necroptotic machinery in response to *Coccidioides*.

5. MOIs are not justified consistently across experiments throughout the manuscript. A short explanation would enable easier reading.

We thank the reviewer for this comment and opportunity to improve the clarity on this point. We used distinct MOIs to interrogate different stages of host–pathogen interactions. Higher MOI (MOI:1) conditions were used to expose all macrophages to a single arthroconidia in culture at early timepoints to record the induction of cell death or inflammasome activation. For spherulation experiments over 3-4 days of culture, a low MOI:0.01 of live arthroconidia (LA) together with dead arthroconidia (FKA) was most conducive for spherulation while also allowing the majority of macrophages to survive the initial exposure to arthroconidia. Using a single MOI was not feasible, as high MOI conditions abrogated spherule development due to rapid macrophage cell death. Additional text has been added in the results section to provide clarity: "*High multiplicity of infection (MOI:1) induced robust early macrophage death and inflammasome activation (Fig. 1D), whereas low MOI (MOI:0.01) preserved macrophage viability and facilitated spherulation*."

6. IL-1β release is shown to require higher MOIs is repeated across conditions, but the physiological relevance is not discussed. Do these MOIs approximate what alveolar macrophages encounter in vivo, or are they supraphysiological?

We thank the reviewer for this comment. The infectious dose of inhaled *Coccidioides* arthroconidia required to establish an acute or chronic infection in the human host remains unclear. However, studies in mice have demonstrated that lethal infection can be established by ~100 arthroconidia. In these *ex vivo* BMDM assays where we are seeking to measure a response from every macrophage in culture, we have exposed macrophages to an MOI:1 to increase the chance that every macrophage encounters an arthroconidia, thereby amplifying IL-1β production. Our ***new data*** now demonstrate a role for alveolar macrophages in sensing arthroconidia and supporting spherulation, which is associated with IL-1β release ***(Figure 7H-J).***

Figure 7

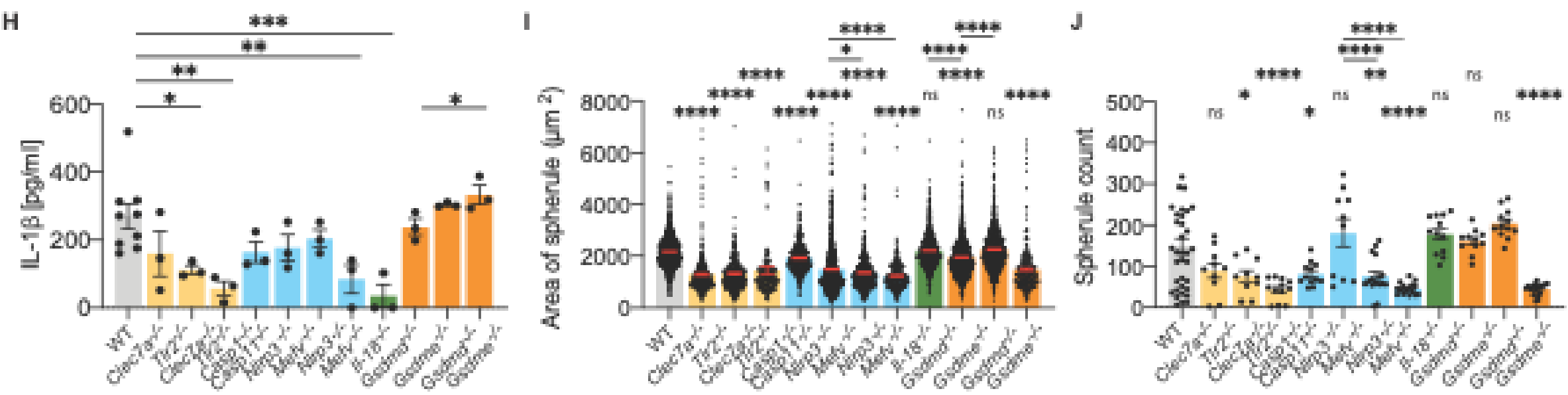

Reviewer #2 (Remarks to the Author):

I co-reviewed this manuscript with one of the reviewers who provided the listed reports. This is part of the Nature Communications initiative to facilitate training in peer review and to provide appropriate recognition for Early Career Researchers who co-review manuscripts.

We thank this reviewer's contribution to our manuscript.

Reviewer #3 (Remarks to the Author):

The authors devote considerable experimental effort to comprehensively analyze how different forms of the dimorphic fungus Coccidioides – arthroconidia (live and dead), spherules (intact and ruptured) - influence cell death and cytokine release of macrophages. The work is largely done in vitro with bone marrow derived macrophages (BMDM). These BMDM are from C57B/l6 mice and a large number of related transgenic strains (n = about 14 strains) with gene defects in inflammasome activation pathways, cell death pathways, TLRs, CLRs and inflammatory cytokines (IL-1B and IL-18). Additional work is done with sophisticated imaging to address how some of these genes influence the behavior of these fungal forms in the BMDM in vitro. At the end of these extensive in vitro studies, an in vivo experimental infection is performed to look at survival and lung fungal burden with two strains of knockout mice (Caspase 1 and Caspase 11) in an effort to establish a biological link with this disease. Regrettably, the depth of in vivo studies is limited and superficial with no cellular or molecular analysis. Thus, I do not find the in vivo mouse studies to be a compelling translation of in vitro findings or link to the biology of this disease. On balance then, the study offers a comprehensive survey of a large cohort of knockout mice with too little effort at the end of the story to validate in vitro findings at a cellular and molecular level. As one example, the investigators could interrogate alveolar macrophages in wild type and representative knockout mouse strains for important pathways, perhaps harboring different forms/stages of the fungus to validate the key in vitro observations they made using BMDMs.

We thank the reviewer for this comment. To extend the *in vivo* observations, we performed additional experiments using primary alveolar macrophages from 12 different genotypes including WT. This ***new data (Figure 7H-J)*** now illustrates the response of alveolar macrophages to *Coccidioides* infection by live arthroconidia (LA). These data include IL-1β secretion and ability to support spherule development. Again, we find a key role for Clec7a-TLR2, NLRP3-pyrin inflammasomes, GSDMD-GSDME, and MLKL in the development of spherules and IL-1β production.

Figure 7

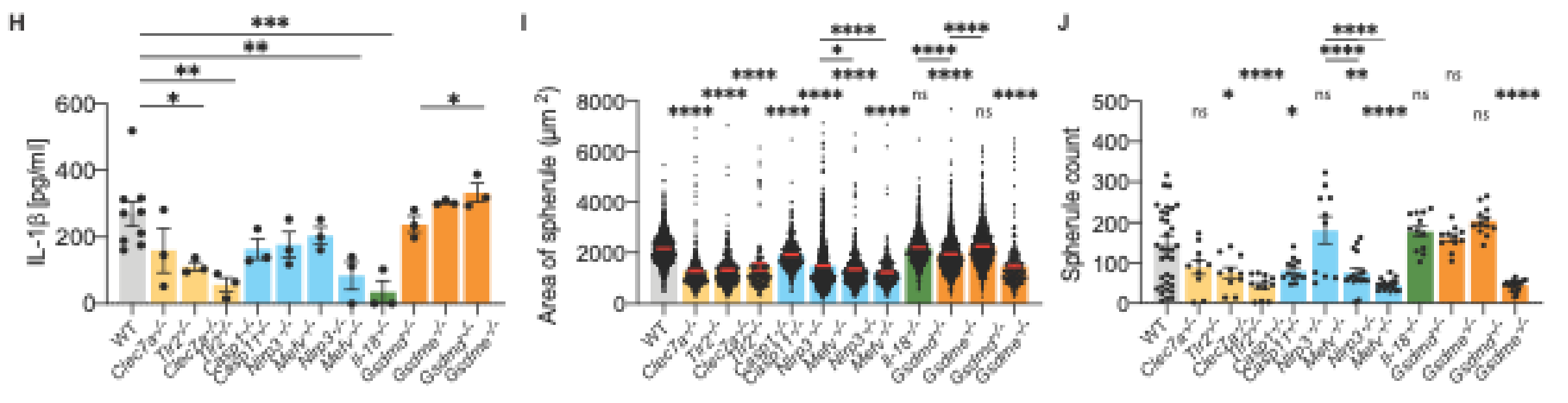

Furthermore, we have added ***new data (Figure 7G)*** to extend the analysis of our *in vivo* multiplexing immunofluorescence Orion experiments to compare the infiltration of neutrophils, NET formation, and accumulation of resident macrophages into ruptured spherules of WT versus *Casp1*$^{-/-}$*Casp11*$^{-/-}$ infected lungs, and have added corresponding additional text in the Results section: "*Quantification of the Ly6G*$^{+}$ *area showed reduced neutrophil infiltration into ruptured spherules in caspase-1/11–deficient lungs; however, neutrophils still formed NETs, as indicated by H3Cit*$^{+}$ *staining within ruptured spherules (Figure 7G). In contrast, CD11c and F4/80 staining demonstrated increased*

accumulation of resident macrophages within ruptured spherules in caspase-1/11–deficient mouse lungs (Figure 7G)".

Figure 7

G

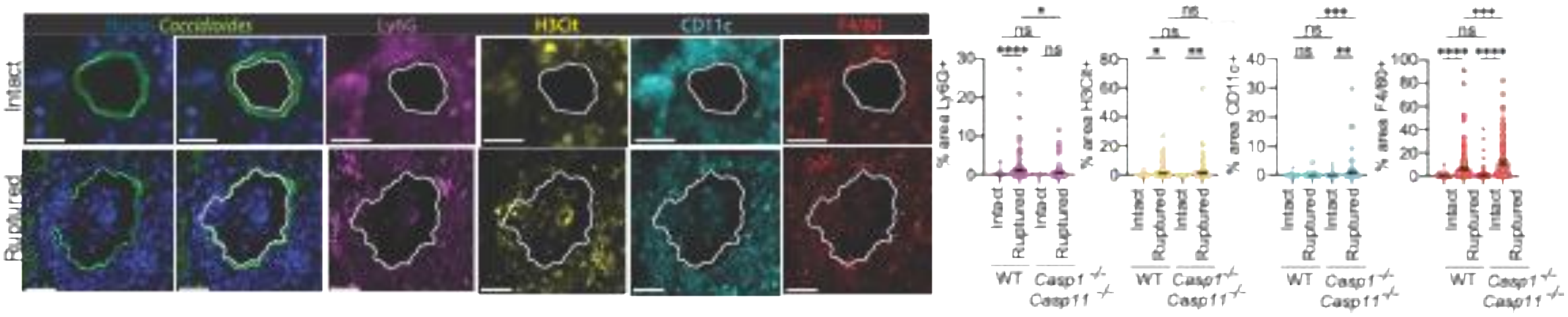

Reviewer #4 (Remarks to the Author):

In the manuscript "Dual Activation of NLRP3 and Pyrin Inflammasomes Mediates Host Response to a Human Fungal Pathogen," Yau et al. report the role of inflammasomes in Coccidioides infection. The authors investigated the interactions of Coccidioides at different developmental stages, including arthroconidia, spherules, and endospores, with ferroptosis and inflammasome pathways that control Coccidioides viability, IL-1β production, and macrophage death. They demonstrate the involvement of NLRP3 and pyrin inflammasomes, and inflammatory cell death signaling involving gasdermin D, and MLKL during the host response to Coccidioides. This manuscript has significant weaknesses, such as insufficient data and a lack of mechanistic insights. Specific comments are included below.

1. A significant weakness of the paper is that the conclusions are not fully supported by data, and in some instances, the data contradict the conclusions. It is concluded that the NLRP3 inflammasome protects from the dissemination of Coccidioides. But Coccidioides infection was examined in NLRP3 KO mice. Caspase-1/11 KO mice data are insufficient to conclude a role for NLRP3.

We thank the reviewer for this comment and apologise for the confusion and accidental reference to NLRP3. Wording in the results have been edited to more accurately reflect the role of Caspase-1 protein in *Coccidioides* infection-triggered weight loss of mice. To simplify our message, we have corrected the subtitle to this: "*Activation of Caspase-1/11 exacerbates coccidioidomycosis in mice*".

2. Weight loss and mortality were reduced in caspase-1/11 KO, which contradicts the conclusion that the caspase-1/11 signaling may be important for limiting Coccidioides disease severity.

We thank the reviewer for this comment and apologise for the misstatement when referring the KO mice. Wording in the results have been edited to more accurately reflect the role of Caspase-1 protein in weight loss of *Coccidioides*-infected mice.

3. Another weakness is the lack of mechanistic insights into the phenotypes observed. For instance, how do arthroconidia activate pyroptotic, necroptotic, and ferroptotic pathways?

We thank the reviewer for this comment and to clarify some of the mechanistic insights gleaned from these cellular studies in genetically-modified mice. We performed additional experiments in alveolar macrophages and BMDM to explore the nature of host-pathogen interactions ***(see new Figures 1G-H, 4F-H,and 7H-J).*** These findings help us to understand the types of fungal ligands that might be triggering innate immune pathways in neutrophils and macrophages, based on roles for TLR2/Dectin-1, MLKL, NLRP3/pyrin, Caspase-1/ASC, GSDMD/GSDME, and IL-18/IL-1β. Moreover, the current work has made significant progress by examining innate immune responses to each stage of the *Coccidioides* life cycle including arthroconidia, spherules, and endospores, and has done so in 3 different myeloid cell types including BMDM, alveolar macrophages, and neutrophils. Both live and fixed *Coccidioides* have been used to investigate host-pathogen interactions.

Figure 1

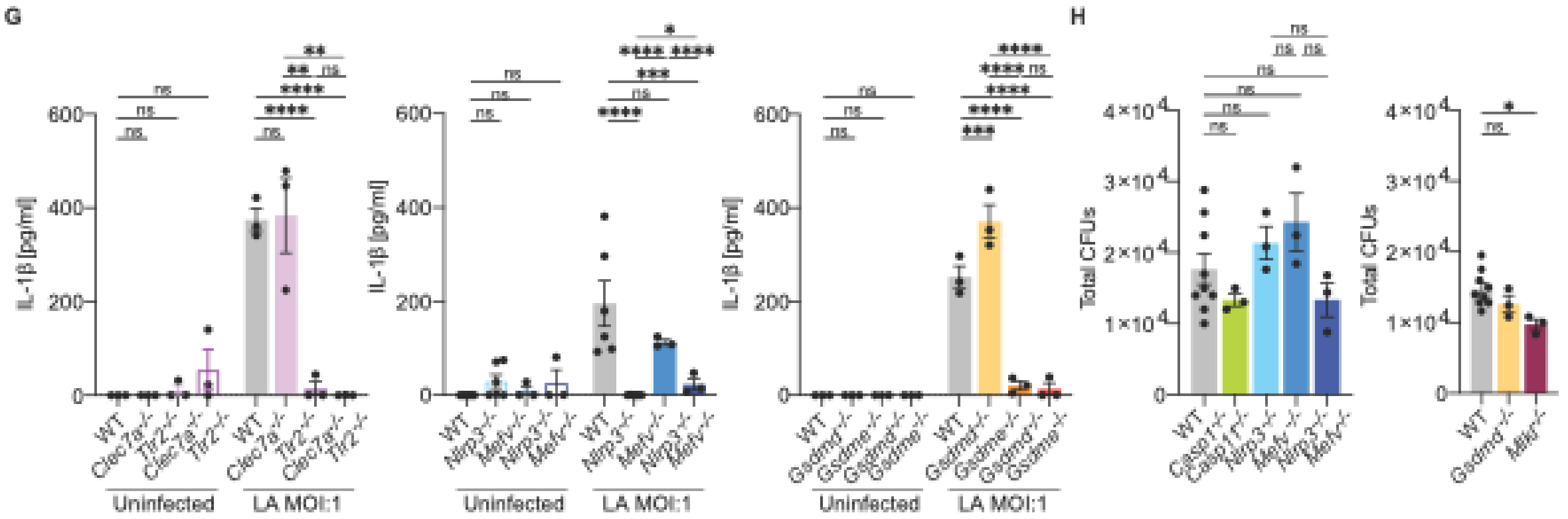

Figure 4

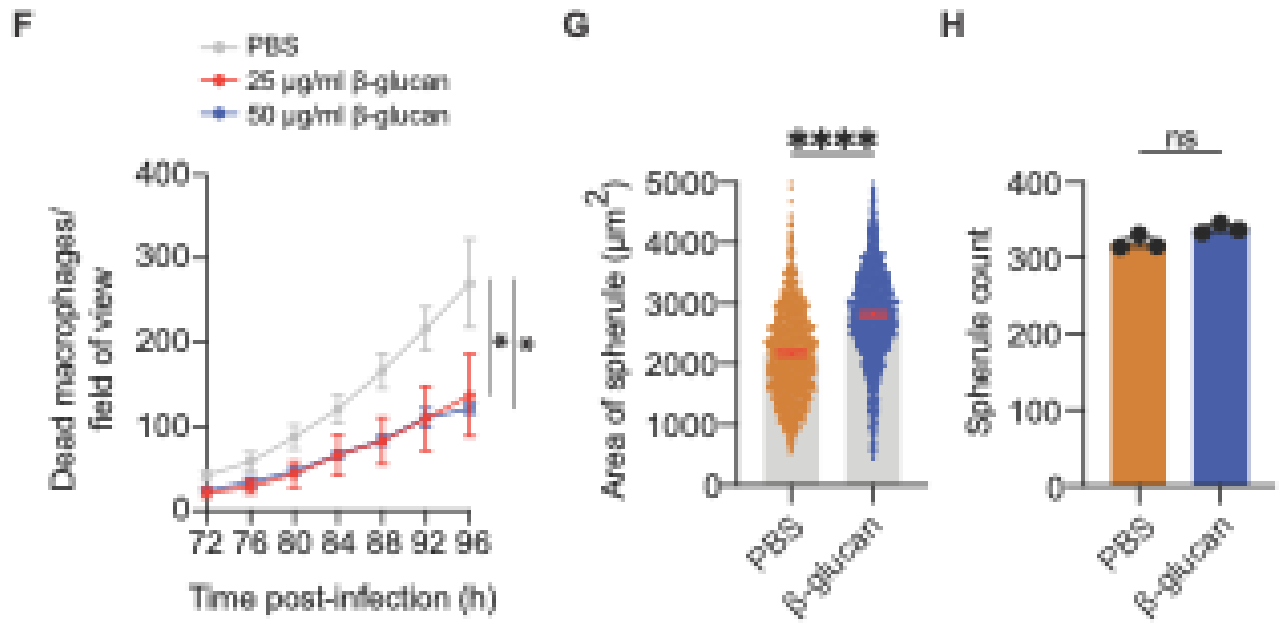

Figure 7

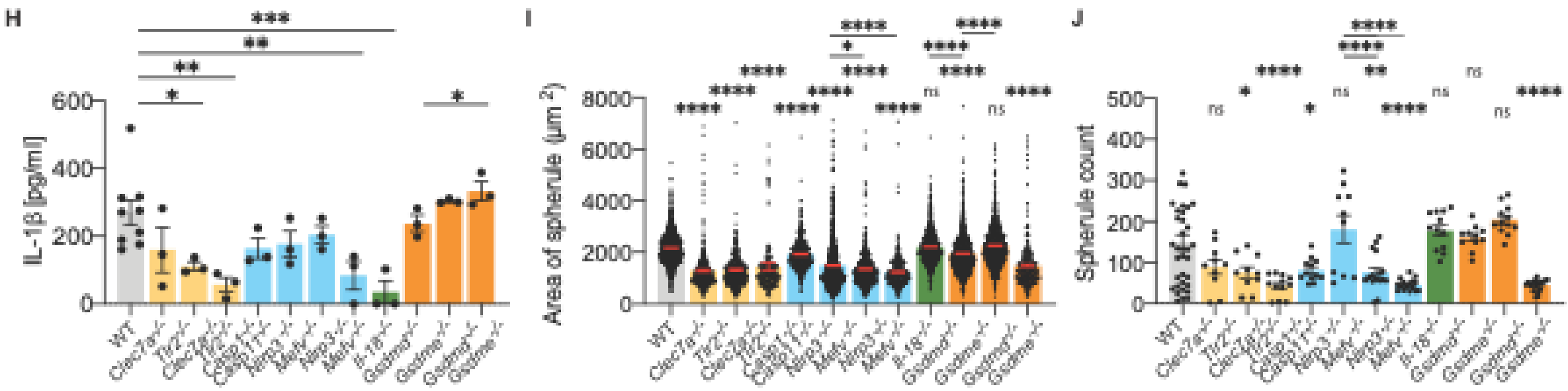

Specifically, we show that live arthroconidia can induce IL-1β secretion in a MOI-dependent manner (Figure 1). To support this claim, we added ***new data (Figure 1G)*** showcasing IL-1β secretion by BMDMs is dependent on TLR2, NLRP3, and GSDME activation. In parallel, arthroconidia triggered macrophage cell death via MLKL, suggesting that necroptotic signaling is also engaged (Figure 1), consistent with findings in neutrophils (Figure 7L). Together, these results indicate that arthroconidia activate multiple regulated cell death pathways in macrophages and neutrophils, coordinating inflammasome activation, necroptotic machinery, and cytokine release.

Ferroptotic lipid peroxidation is utilized by macrophages to limit arthroconidia viability, as inhibition of ferroptosis by Lip-1 increases the viability of arthroconidia and simultaneously increases macrophage cell death (Figure 2). Supplementing iron in macrophage cultures to augment iron-dependent lipid peroxidation reduces the viability of arthroconidia and reduces macrophage cell death. We have improved the text for clarity on these points.

Further work studying macrophage-*Coccidioides* interactions using genetically-modified *Coccidioides* lacking candidate virulence factors would help identify the specific fungal ligands that activate macrophage and neutrophil innate immune signaling pathways. However, gene targeting in *Coccidioides* is currently inefficient and technically challenging, and certainly well beyond the scope of the manuscript.

4. The rationale for specifically testing the role of pyrin (among other inflammasomes) in arthroconidia-induced macrophage death and IL-1β release is not clear, and the pyrin inflammasome part seems superficial.

We observed phenotypes that were ASC- and Caspase-1-dependent but only partially NLRP3-dependent. This likely excluded NLRP1a/b/c because this inflammasome is ASC-independent in mice (PMID:23219391). We also considered it less likely to be AIM2- or NLRC4-dependent given their known ligands (cytosolic dsDNA, flagellin, type III secretion systems). Our prior experience with mammalian mutations in Wdr1 causing autoinflammation via effects on actin depolymerization (PMID: 26008898) led us to hypothesize that a pyrin inflammasome would be activated due to the enormous disruption to the actin cytoskeleton in BMDM, neutrophils, and alveolar macrophages when encountering large fungal structures including arthroconidia, spherules, and endospores. This hypothesis appears correct, as numerous assays show dependencies on NLRP3 and pyrin inflammasomes, with additive effects upon genetic disruption of both NLRP3 and pyrin (Figure 1F, 5C, 5D, 7L, ***new Figure 1G***). Collectively, our data suggests both cooperative and specialized roles for NLRP3 and pyrin inflammasome complexes in the response of macrophages and neutrophils to *Coccidioides*. To further support the dual role NLRP3 and pyrin inflammasomes in *Coccidioides* infection, we have added ***new data (Figure 7H-J)*** which demonstrate that both NLRP3 and pyrin is activated upon sensing of ruptured spherules by alveolar macrophages in sensing arthroconidia and is required for optimal spherule formation and growth.

5. Did live arthroconidia (LA) induced macrophage death and IL-1β release require LPS priming? If not, how do live arthroconidia prime macrophages? via dectin-1?

Live arthroconidia (LA) can induce IL-1β secretion in a MOI-dependent manner (Figure 1). To support this claim, we have added ***new data (Figure 1G)*** showcasing LA-induced IL-1β secretion by BMDMs that is dependent on TLR2, NLRP3, and GSDME. We hypothesis that the TLR2 ligand is likely a *Coccidioides* lipopeptide or chitin.

6. Do arthroconidia induce the activation of GSDME/GSDMD/MLKL in macrophages?

We have also added ***new data (Figure 1H)*** which demonstrate LA triggers macrophage cell death in part via MLKL but not GSDMD or GSDME. Furthermore, existing data show that the reduction in the death of *Mlkl*$^{-/-}$ BMDM is correlated with a reduction in viable arthroconidia (***new data***, ***Figure 1G***).

7. Whether arthroconidia induced ferroptosis in infected cells remains unclear, as Lip-1 did not block macrophage death but increased it following LA infection. Therefore, more data demonstrating lipid peroxidation and ferroptosis is needed to support the conclusion.

We thank the reviewer for this comment. This finding was also a surprise to us, and was reflected in the subsection title: "*Ferroptotic lipid peroxidation kills arthroconidia without altering macrophage viability*". We also noted that "*Lip-1 did not block macrophage death but increased it*". We went on to say that: "*These data suggest that iron-dependent lipid peroxidation may damage arthroconidia as a consequence of ferroptosis in BMDM*". Here we used 'ferroptosis' as a reference to a signaling pathway rather than a form of cell death – instead we have now edited that text to refer ferroptotic lipid peroxidation. Wording in the abstract and the discussion have also now been edited to reflect the role of ferroptotic lipid peroxidation, rather than ferroptosis, in fungal control.

Additionally, to support the role of ferroptotic lipid peroxidation, we show that Fer-1 inhibition of ferroptotic signalling increases macrophage death similar to the other ferroptosis inhibitor Lip-1 (***Response_to_Reviewer_Figure 4***).

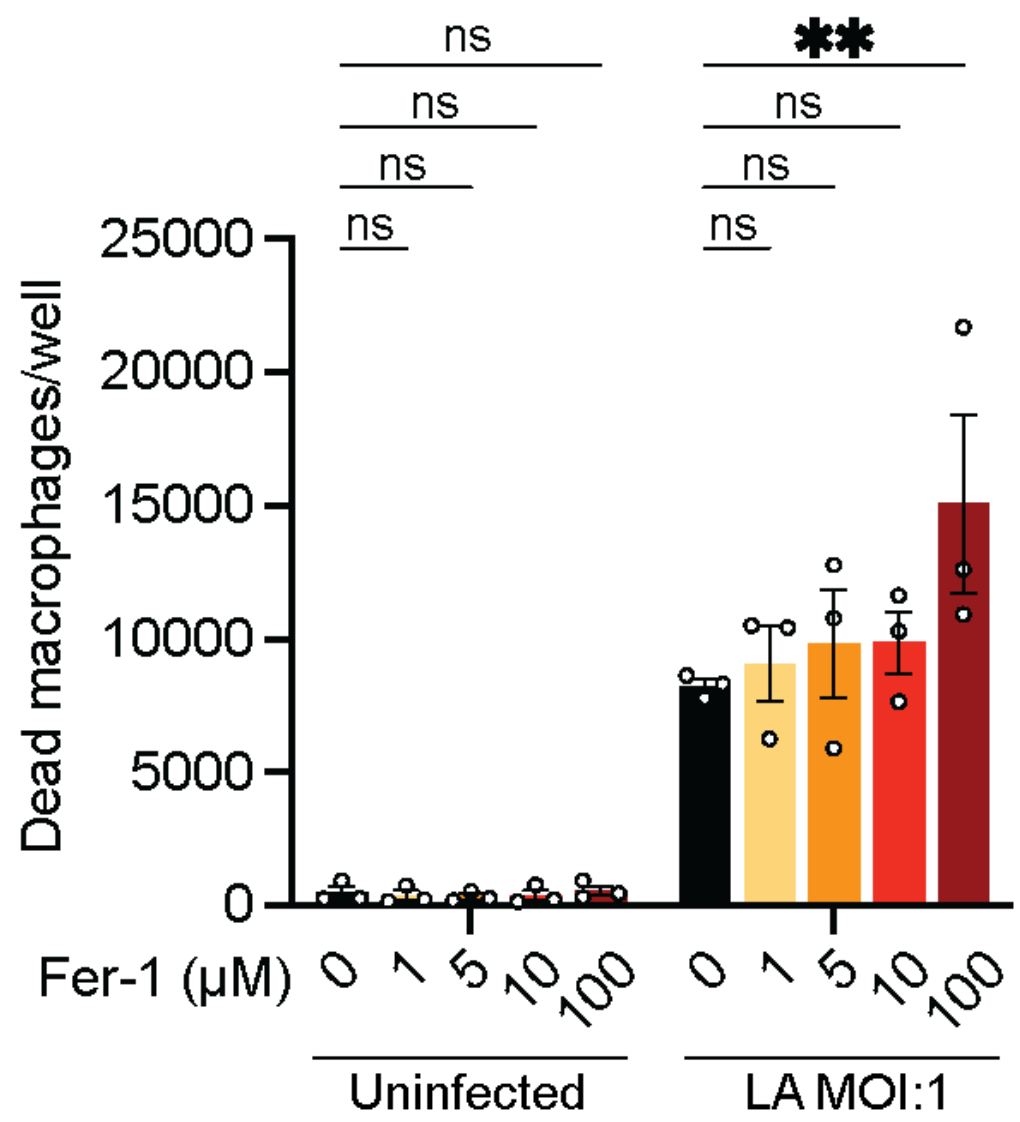

Response_to_Reviewer_Figure 8: Cell viability by live-cell imaging and automated image analysis (whole well scans) of WT BMDMs infected with LA at MOI:1, and treated with ferrostatin-1 (Fer-1) at 1, 5, 10, and 100 µM. Mean ± SEM, n = 3, $^{**}P < 0.005$, ns: not significant, WT vs knockouts, two-way ANOVA.

8. Confirmation of cell death with the LDH assay is recommended.

We thank the reviewer for this suggestion. We elected to use PI staining to provide single-cell resolution over a timecourse, lower background, and the ability to identify which cells are undergoing cell death—advantages not achievable with bulk LDH-release endpoint assays. This approach is consistent with methods used by numerous cell death laboratories.

9. Interestingly, killed arthroconidia-induced IL-1β release was Nlrp3 dependent, but live arthroconidia-induced cell death was Nlrp3 independent. This discrepancy should be addressed.

We thank the reviewer for this comment and apologise for the confusion. We have performed additional experiments to address cell death and IL-1β production. These data are included in

Figure 1. LA- and FKA-induced IL-1β release are NLRP3-dependent (Figure 1B, 1F). The text has also been updated to provide clarity on this question.

10. How do killed arthroconidia inhibit cell death response to live arthroconidia and promote spherule formation in macrophages?

This remains an interesting question how the addition of killed arthroconidia to live arthroconidia: (1) promotes spherule formation; and (2) inhibits macrophage cell death.

We hypothesize that: (1) viable macrophages are required to promote spherule formation; (2) phagocytosis of arthroconidia (by viable macrophages) generates an intracellular environment that promotes the transition of arthroconidia to spherules and not hyphae; and (3) the presence of killed arthroconidia may prime macrophages for innate immune responses to live arthroconidia by enhancing phagocytosis, fungal sensing receptors, cytokine production, and pro-survival protein expression. The reduction in macrophage death when killed arthroconidia are added to cultures do not appear to be dependent on Caspase-1, GSDMD-GSDME, or MLKL (Figure 1F). However, loss of TLR2, NLRP3-pyrin, or IL-18 all reduce the size of spherules. These findings indicate that macrophage-*Coccidioides* interactions influence spherule morphogenesis in part via autocrine and/or paracrine production of IL-18 (Figure 5F-5J). Resolving these complex changes that enable the transition of arthroconidia to spherules in the presence of macrophages is beyond the scope of this manuscript.

A recent preprint also supports these observations by noting that the presence of macrophages promotes the frequency of spherulation:
(https://www.biorxiv.org/content/10.1101/2025.10.05.680493v1).

11. Line 299-700: Figures were cited incorrectly.

We thank the reviewer for noting this error. The text has now been corrected.
